# The RNA landscape of the human placenta in health and disease

**DOI:** 10.1038/s41467-021-22695-y

**Published:** 2021-05-11

**Authors:** Sungsam Gong, Francesca Gaccioli, Justyna Dopierala, Ulla Sovio, Emma Cook, Pieter-Jan Volders, Lennart Martens, Paul D. W. Kirk, Sylvia Richardson, Gordon C. S. Smith, D. Stephen Charnock-Jones

**Affiliations:** 1 Department of Obstetrics and Gynaecology, University of Cambridge, NIHR Cambridge Biomedical Research Centre, Cambridge, UK; 2 Centre for Trophoblast Research (CTR), Department of Physiology, Development and Neuroscience, University of Cambridge, Cambridge, UK; 3 Computational Omics and Systems Biology Group, Department of Biochemistry, Ghent University, Ghent, Belgium; 4 MRC Biostatistics Unit, Cambridge Institute of Public Health, University of Cambridge, Cambridge, UK; 5 Cambridge Institute of Therapeutic Immunology & Infectious Disease, Jeffrey Cheah Biomedical Centre, University of Cambridge, Cambridge, UK; 6 Present Address: Functional Genomics, GlaxoSmithKline Limited, Stevenage, Hertfordshire UK

**Keywords:** Data publication and archiving, Gene expression, Endocrine reproductive disorders

## Abstract

The placenta is the interface between mother and fetus and inadequate function contributes to short and long-term ill-health. The placenta is absent from most large-scale RNA-Seq datasets. We therefore analyze long and small RNAs (~101 and 20 million reads per sample respectively) from 302 human placentas, including 94 cases of preeclampsia (PE) and 56 cases of fetal growth restriction (FGR). The placental transcriptome has the seventh lowest complexity of 50 human tissues: 271 genes account for 50% of all reads. We identify multiple circular RNAs and validate 6 of these by Sanger sequencing across the back-splice junction. Using large-scale mass spectrometry datasets, we find strong evidence of peptides produced by translation of two circular RNAs. We also identify novel piRNAs which are clustered on Chr1 and Chr14. PE and FGR are associated with multiple and overlapping differences in mRNA, lincRNA and circRNA but fewer consistent differences in small RNAs. Of the three protein coding genes differentially expressed in both PE and FGR, one encodes a secreted protein FSTL3 (follistatin-like 3). Elevated serum levels of FSTL3 in pregnant women are predictive of subsequent PE and FGR. To aid visualization of our placenta transcriptome data, we develop a web application (https://www.obgyn.cam.ac.uk/placentome/).

## Abstract (web summary)

Placental dysfunction can have catastrophic or barely discernible effects ranging from miscarriage to apparently normal birth. Here the authors present a comprehensive analysis of the human placental transcriptome and identify circular RNAs and piRNAs.

## Introduction

During mammalian development, the placenta is the first organ to form and is responsible for anchoring the embryo to the uterus and mediating nutrient and gas exchange with the mother. The placenta sustains the fetus throughout pregnancy and defects in placentation are at the root of many pregnancy complications. Yet despite its significance for evolution, development, and reproductive health, the placenta is understudied and is commonly omitted from large-scale “-omic” analyses. For example, of the 17,382 samples in the Genotype-Tissue Expression (GTEx) project^1^, a tissue-wide gene expression study, none are placental. However, placental dysfunction underlies a large proportion of maternal and perinatal morbidity and mortality. Worldwide, the burden of mortality due to maternal and perinatal death is equivalent to about half the total burden due to cancer^2^.

RNAs carry out numerous functions in addition to coding for proteins. For example, they play multiple roles in both the nucleus and cytoplasm to regulate transcription. They control nuclear architecture and modulate mRNA stability, translation, and post-translational modification^3,4^. Therefore, knowing the repertoire of RNAs present, informs our understanding of cell and tissue function.

Previous placental transcriptome analyses focused on differences associated with maternal and fetal conditions^5–7^. Initial studies used microarray analyses, followed more recently by RNA-Seq^8–11^. However, these studies are limited in terms of the number of placental biopsies and the depth of sequencing coverage (Supplementary Data 1). In addition, most of the early RNA-Seq studies used oligo-dT primed cDNA synthesis, which prevents analysis of non-adenylated RNAs and selected against small transcripts such as micro-RNA (miRNA) and PIWI-interacting RNAs (piRNAs).

Here we report high-quality RNA-Seq data and provide a comprehensive analysis of the human placenta transcriptome. We isolated total RNA, fractionated by size, and sequenced rRNA-depleted long and small RNAs from 302 human placentas (Supplementary Fig. 1). We first characterize the various types of transcript in the placenta and analyze the transcriptome composition and complexity compared with other human tissues. We then reconstructed the placental transcriptome and report previously unrecognized coding and non-coding transcripts. We show there are multiple abundant circular RNAs (circRNAs) and provide an analysis of dysregulated transcripts in cases of preeclampsia (PE) and fetal growth restriction (FGR). Finally, we demonstrate that maternal serum levels of a protein, follistatin-like 3 (FSTL3), encoded by one of the differentially expressed mRNAs, is predictive of PE and FGR.

## Results

### High-quality placenta RNA-Seq data

We generated 324 total RNA-Seq datasets from 302 placental biopsies of the Pregnancy Outcome Prediction (POP) study cohort^12–14^ and obtained a total of ~33 billion reads (~101 million reads per sample, Supplementary Data 2 and Supplementary Fig. 1). We also generated 328 small RNA-Seq datasets, producing ~6.6 billion reads in total (~20 million per sample, Supplementary Data 3). We identified the presence of decidual contamination in three samples that were excluded from the analysis^15^ (see methods). To aid dissemination and visualization of our placenta transcriptome data, we developed a web application (https://www.obgyn.cam.ac.uk/placentome/) where users can browse: (1) the abundance of long, small and circular transcripts, (2) genes expressed in the placenta in comparison with 49 somatic tissues (GTEx dataset), (3) previously unrecognized transcripts, and (4) differentially regulated transcripts in complicated pregnancies. A screen shot of an example of this is shown in Supplementary Fig. 2.

### Relative abundance and complexity of RNA populations

**Fig. 1:**
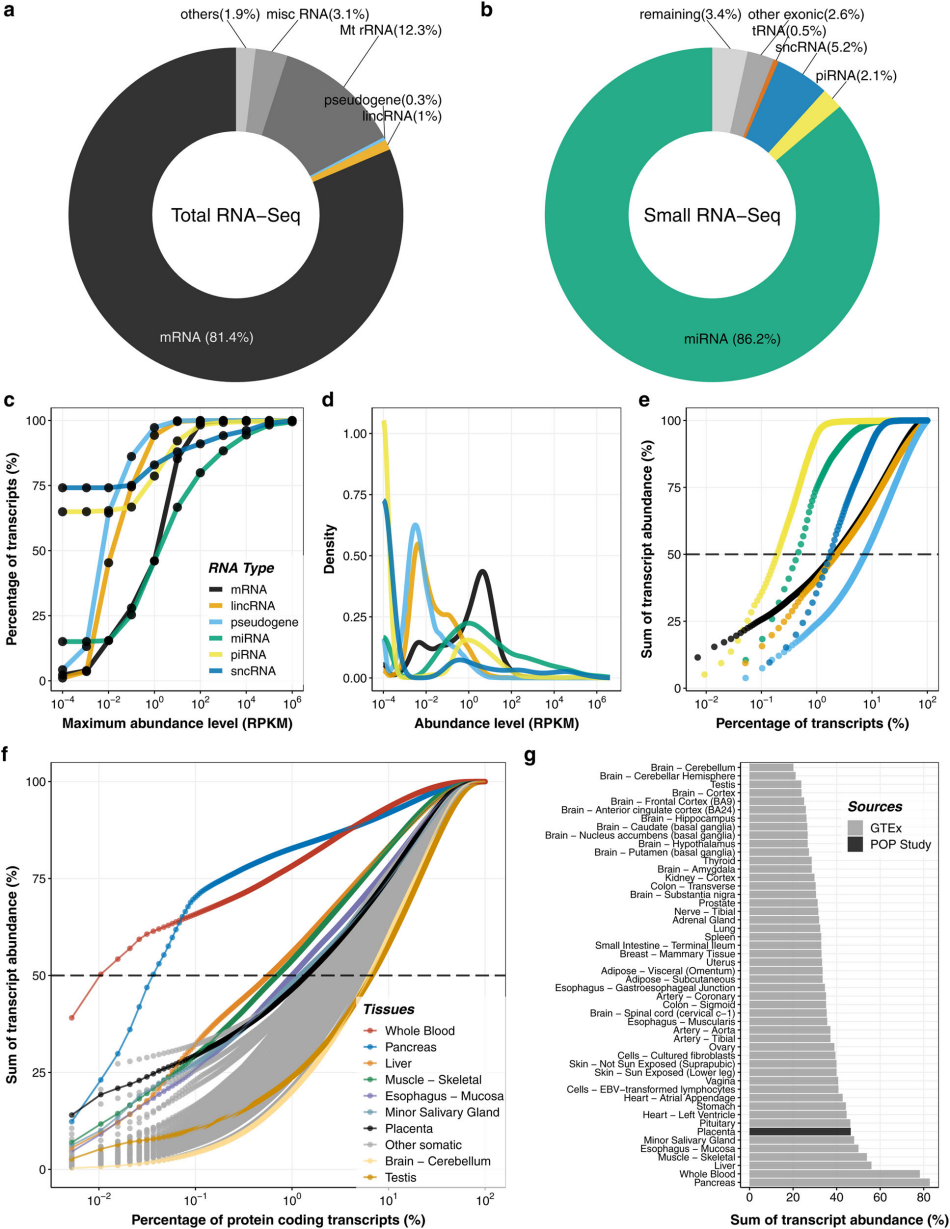
Complexity of RNA transcripts in the placenta. After sequencing and alignment to the human reference genome various RNA biotypes were identified in the placenta. The proportions mapped reads to various types of long (**a**) and small (**b**) RNAs are shown. In (**a**), definitions of RNA types were from the biotypes of Ensembl as follows: mRNA (protein-coding messenger RNA), lincRNA, pseudogenes (processed pseudogene, unprocessed pseudogene, transcribed unprocessed pseudogene, transcribed processed pseudogenes, or pseudogenes), Mt rRNA (mitochondrial ribosomal RNA), misc RNA (non-coding RNA that cannot be classified), others (other remaining biotypes such as antisense, snRNA, snoRNA, processed transcripts). In (**b**), ‘other exonic’ refers to reads mapped to any exonic regions except miRNA, piRNA, tRNA, and sncRNA and ‘remaining’ refers to mapped reads except miRNA, piRNA, tRNA, scRNA, and ‘other exonic’. sncRNAs include the following three types of RNAs: snoRNA (small nucleolar RNA), snRNA (small nuclear RNA), and sRNA (small RNA). RNAs are plotted as the percentage of quantified transcripts against the expression level (**c**) and the frequency of transcripts (density) against expression level (**d**). In (**c**–**d**), the RPKM values have a pseudo-count (0.0001) added to allow plotting on a logarithmic scale. For the density plot (**d**), the probability density functions were estimated using kernel density estimation, where the area under the curve equals one. **e** The placental transcriptome is represented as the cumulative percentage of various RNA biotypes in the current study. **f** The total mRNAs pool is represented as the cumulative percentage of protein-coding transcripts in 50 tissues, including the placenta. In (**e**–**f**), each point represents a transcript (with RPKM > 0.1) and the dashed line represents the TA50, i.e., the percentage of transcripts required to reach half of the total transcript abundance. To reach TA50 in (**f**), the following numbers of protein-coding RNAs are needed: 2 (blood), 7 (pancreas), 110 (liver), 135 (muscle – skeletal), 191 (esophagus – mucosa), 232 (minor salivary gland), and 271 (placenta). **g** For each tissue, the bar chart shows the contribution of the most abundant 1% of mRNAs to the total pool of protein-coding transcripts.

We analyzed long (>200nt) and small RNAs and a large majority of the mapped reads corresponded to messenger RNAs (mRNAs) and miRNAs, respectively (Fig. 1a–b). We found that the majority of annotated pseudogenes, small non-coding RNAs (sncRNAs), long intergenic non-coding RNAs (lincRNAs), and piRNAs were not expressed or were expressed very weakly (<0.1 RPKM, Read Per Kilobase of exon model per Million mapped reads). By contrast, most protein-coding transcripts and mature miRNAs were present above this threshold. For example, 86, 75, 74, 67% of pseudogenes, sncRNAs, lincRNAs, and piRNAs, respectively were either not detected or were detected at <0.1 RPKM, whereas only 25 and 28% of miRNA and protein-coding RNAs were below this threshold (Fig. 1c and Supplementary Data 4). Within the different RNA classes the populations were skewed (Fig. 1d). For example, three-quarters (75%) of annotated sncRNAs were undetectable (i.e., RPKM = 0; Fig. 1c), although 5.2% of reads were mapped to that RNA biotype (second largest proportion behind the miRNAs (Fig. 1b)). Similarly, two thirds (65%) of annotated piRNAs were undetectable in the placenta (i.e., RPKM = 0) but 1.7% were expressed at RPKM > 100. For the miRNAs, 15% were undetectable but 20%, 12 and 6% were expressed at RPKM > 100, >1000 and >10,000, respectively. The majority (10,506, 53%) of coding mRNAs were present at 1–100 RPKM.

We ranked transcripts by their abundance and determined their contribution to the total for that transcript biotype in the placenta (Fig. 1e). We found that the percentage of transcripts required to reach half of the total transcriptome abundance (TA50) varied substantially depending on the RNA biotypes: piRNAs 0.2% (*n* = 21), miRNAs 0.5% (n = 9), sncRNAs 1.8% (*n* = 13), lincRNAs 2.3% (*n* = 46), protein-coding transcripts 2% (*n* = 295), and pseudogene transcripts 7.5% (*n* = 146). We then focused on protein-coding mRNAs and compared the placental TA50 to other somatic tissues in the GTEx dataset (v8.p2)^1^ as an indicator of the complexity of this transcript biotype. The placenta is ranked 7th out of 50 tissues studied, whereas blood (ranked 1, i.e., the tissue with the least complex pool of protein-coding transcripts) required only two hemoglobin mRNAs (*HBB* and *HBA2*). The testis and the brain (cerebellum) had the most complex pool of protein-coding transcripts with 1289 and 1225 annotated mRNAs, respectively, required to reach TA50 (Fig. 1f–g).

### Transcripts enriched in the placenta

We used the *Tau* score (with some additional filtering conditions, see Methods) to define the tissue enriched transcripts. We found 1389 such protein-coding genes expressed in the placenta and the 49 somatic tissues in the GTEx dataset (Supplementary Fig. 3 and Supplementary Data 5). There were 71 placenta enriched protein-coding transcripts which is significantly more than expected by chance (observed/expected 1.48, chi-squared *P* < 2.2 × 10^−16^), and the placenta is ranked 4^th^ behind the testis, liver, and skin (671, 143, and 77 genes respectively). This includes several pregnancy-related genes, of which *CSH1* and *CSH2* (Chorionic Somatomammotropin Hormone 1 and 2, respectively) were the most abundant, and all the members of the *PSG* (pregnancy-specific glycoprotein) family, except *PSG10P* (a pseudo-gene). In addition, several groups of closely related genes also met the criteria to be defined as placenta enriched, e.g., galectins (*LGALS13*, also known as pregnancy protein 13, *PP13*), *LGALS14*, and *LGALS16*), two members of the melanoma antigen family (*MAGEA8* and *MAGEA10*), three chorionic gonadotropin subunit beta genes (*CGB3, CGB5*, and *CGB8*), two members of X antigen family (*XAGE2* and *XAGE3*) and four endogenous retrovirus genes (*ERVW-1*, *ERVFRD-1*, *ERVV-1,* and *ERVV-2*). Using the same criteria we also identified 1,200 tissue enriched long non-coding genes, 74 of which were specifically enriched in the placenta. The placenta is ranked second, (observed/expected 1.54, chi-squared *P* < 2.2 × 10^−16^), behind the testis (first), followed by the liver (third) (Supplementary Fig. 3 and Supplementary Data 6). Interestingly, we found one of the placenta enriched lincRNAs was encoded by endogenous retrovirus (*ERVH48-1*). We inspected all the endogenous retrovirus genes and identified two additional protein-coding endogenous retrovirus genes (*ERV3-1* and *ERVMER34-1*) as placenta enriched using slightly less stringent criteria (Supplementary Fig. 3 and Supplementary Data 7). Of note, the mRNAs encoding the major histones are not adenylated^16,17^ and thus are greatly underrepresented in the GTEx data which was generated using poly-A^+^ selected RNA.

### Highly abundant circRNAs and small RNAs

circRNAs is generated by back-splicing and lacks a poly-adenylated tail. These can be identified by RNA-Seq using the non-oligo-dT based methods employed in the present study. Such Ribo-Zero methods have been described as the “gold-standard” for the detection of circRNAs^16^. Using RNA-Seq data generated from both non-oligo-dT (discovery datasets) and oligo-dT primed (negative control) libraries, we found 3304 predicted circRNAs present in at least 30% of the POP study cohort (POPS30, i.e., ≥89 out of 295 samples) from non-oligo-dT libraries. 25 circRNAs were also detected in oligo-dT libraries (Supplementary Data 8 and 9) and we excluded these from the analysis as they were likely to be false positives. Of the remaining 3279 circRNAs, 169 were detected in every placental sample. We investigated genes hosting multiple circRNAs and found that there were 16 genes harboring at least 10 circRNAs, including *PAPPA* and *PAPPA2* which host 33 and 26 circRNAs, respectively (Supplementary Data 10).

**Fig. 2:**
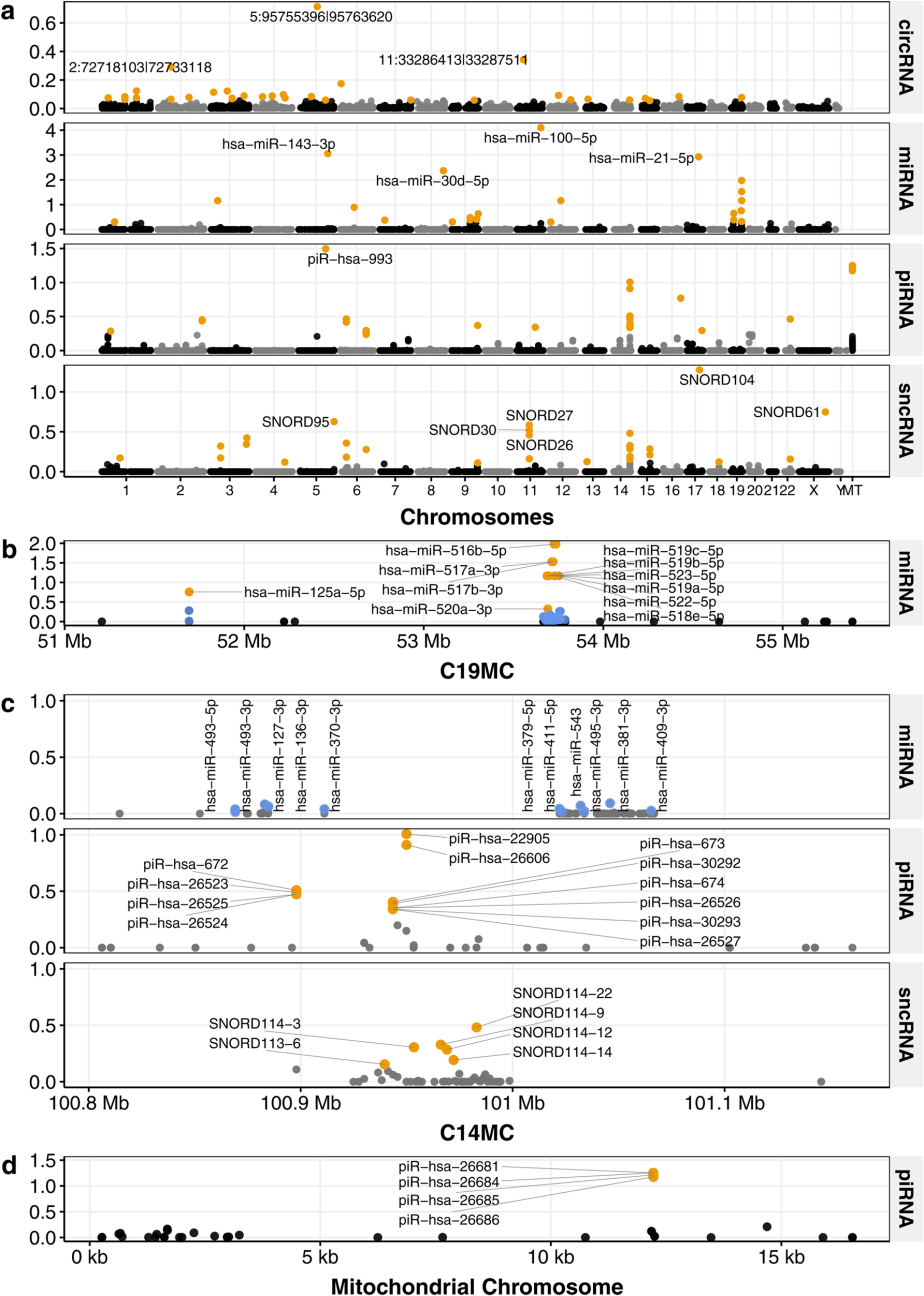
Manhattan plots showing relative abundance of small RNAs. The relative abundance of individual transcripts, represented by dots, are shown for: **a** circRNAs, miRNAs, and piRNAs across all chromosomes; **b** clusters of miRNA on chromosome 19 (C19MC); **c** a cluster of miRNA, piRNA, and sncRNA on chromosome 14 (C14MC); **d** a mitochondrial cluster of piRNA. Transcripts within the most abundant 1% of circRNAs and miRNA, and within the most abundant 0.1% of piRNAs are colored orange. The relative abundance of piRNAs and miRNAs are calculated from the mean RPKM values divided by 10^6^. In the C19MC (**b**) and C14MC (**c**), miRNAs are colored blue if they are between the most abundant 1 and 5% range. The circRNAs are based on 3,279 circular RNAs predicted to be present in at least 30% of the cohort (POPS30) and their relative abundances are calculated from the normalized back-spliced read counts divided by 10^4^. See Supplementary Data 8, 11, 13, and 14 for abundance details of circRNAs, mature miRNAs, piRNAs, and sncRNAs, respectively.

We further studied highly expressed small RNAs and investigated their genomic loci. We found miR-100-5p (chr11:122,152,275-122,152,296; reverse-strand) is the most abundant mature miRNA in the placenta, followed by miR-143-3p, miR-21-5p, and miR-30d-5p. We observed marked skewing of the population of miRNAs with the 20 most abundant mature miRNAs (i.e., top 1%) accounting for ~75% of the total population of miRNAs (Fig. 2a and Supplementary Data 11). Furthermore, ten of these are located within a 66Kb region on chromosome 19 (chr19:53,686,484-53,752,432; forward-strand, Fig. 2b). This miRNA cluster is known as the chromosome 19 microRNA cluster (C19MC, chr19:53,641,443-53,780,750) where 46 genes encode 59 mature miRNAs^18^. In our data, 33 and 10 mature miRNAs of the C19MC were within the most abundant 5 and 1% mature miRNAs (Fig. 2b) and the expression level was negatively correlated with that of 27 reported target mRNAs based on miRTarbase^19^ (*r* = −0.5, *P* = 2.3 × 10^−3^; Supplementary Fig. 4a; Supplementary Data 12). Gene Ontology (GO) analysis of the 27 binding targets showed that GO terms associated with DNA binding, protein phosphorylation, regulation of growth, regulation of the apoptotic process, and response to cytokine and oxidative stress were significantly overrepresented (Supplementary Fig. 4b). Another miRNA cluster has been described in chromosome 14 (C14MC) and it is embedded between the paternally imprinted *DLK1* and *DIO3* genes. However, we found that these miRNAs were much less abundant compared with those from the C19MC—only 11 miRNAs were among the most abundant 5% mature miRNAs and none were among the top 1% (Fig. 2c).

For piRNAs (Supplementary Data 13), we identified piR-hsa-993 (also known as piR-30840, chr5:138,561,046-138,561,073) as the most abundant piRNA (Fig. 2a), followed by piR-hsa-26681, piR-hsa-26684, piR-hsa-26685, and piR-hsa-26686 which are all mitochondrial piRNAs (chrM:12,207-12,237 bp, Fig. 2d). Interestingly, we found two additional piRNA clusters in chromosome 14 (chr14:100,897,922-100,983,942; forward-strand) and chromosome 1 (chr1:30,935,742-30,968,195; reverse-strand) with 21 and 7 piRNAs, respectively, which are within the most abundant 1% of piRNAs (Fig. 2d). The chromosome 14 piRNA cluster is within the C14MC and it spans across two lincRNAs (AL117190.1 and MEG8 (maternally expressed 8, small nucleolar RNA host gene)) and 10 snoRNAs (small nucleolar RNA) from SNORD113 and SNORD114 (Fig. 2c). The region of chromosome 1 with the piRNA cluster also hosts the protein-coding gene PUM1 (Pumilio RNA binding family member 1).

For sncRNAs (sncRNA; Supplementary Data 14), which include snoRNA (small nucleolar RNA), snRNA (small nuclear RNA), and sRNA (small RNA), SNORD104 and SNORD61 are the two most abundant sncRNAs (Fig. 2a) and both account 15% of total abundance of sncRNAs. We found two highly abundant sncRNA clusters in chromosome 11 and 14 where four and six snoRNAs, respectively, are within the most abundant 1% (Fig. 2a). The chromosome 11 snoRNA cluster is embedded in the intron of *SNHG1* (Small Nucleolar RNA Host Gene 1) and the chromosome 14 snoRNA cluster is within the C14MC (Fig. 2c)—the latter was reported to be maternally imprinted.

### Novel miRNAs

We analyzed the small RNA-Seq datasets using miRDeep2 and identified 4,351 novel miRNAs (i.e., not reported in mirBase). We used two additional tools, *sRNAbench*^20^ and *miRge2.0*^21^, and found 158 and 1,161 novel miRNAs, respectively. From the miRNAs predicted by miRDeep2, we selected those supported either by *sRNAbench* or *miRge2.0* for which the genomic features overlap by at least 30% (see “Methods” for details). With this approach, we finally selected a total of 141 novel miRNAs (Supplementary Data 15), 45 of which were supported by miRCarta (v1.1), a database of predicted miRNAs. Of note, there was little agreement between *sRNAbench* and *miRge2.0*—only one was predicted by both tools (but this one was not predicted by miRDeep2). Imposing a requirement that the novel miRNA be present in 30% or 100% of samples reduced the number to 119 and 9 respectively (Supplementary Figure 5c). There was a large number of high-depth (i.e., >10×) mapped reads which did not overlap with miRNAs (both known and novel), piRNAs or exonic boundaries of currently available gene annotations (Fig. 1b). However, while these ‘remaining’ reads can be assembled into 18,511 loci (which we termed ‘novel small RNA; Supplementary Data 16 and Supplementary Fig. 6) they are not uniformly present in the sample set. The number dropped rapidly to 381 and 24 at sample frequency thresholds of 30% and 100%, respectively (Supplementary Fig. 5c).

### Transcriptome reconstruction and unrecognized transcripts

We reconstructed the placental transcriptome using 295 total RNA-Seq datasets from 302 placentas (Supplementary Figure 1). Using StringTie^22^, a transcriptome assembly tool, and Ensembl as a reference transcript annotation, 380,807 placental transcripts (90,204 loci) were reconstructed, of which 262,030 transcripts (61,499 loci) were present at least at 0.1 RPKM (Supplementary Fig. 5). However, the number of potentially novel transcripts (i.e., a ‘potentially novel isoform’, ‘within a reference intron’, and ‘unknown intergenic’ transcripts) drops rapidly as the minimum sample frequency threshold is raised (Supplementary Fig. 5b). The most striking example is ‘unknown intergenic transcripts’, none of which were supported by more than one sample and are unlikely to be functionally relevant. We compared our placental transcriptome (assembled using Cuffcompare) with three alternative meta-assemblers: Gffcompare (https://github.com/gpertea/gffcompare), StringTie-merge, and TACO^23^. The performance of Cuffcompare^24^ is very similar to that of Gffcompare and TACO outperformed StringTie-merge as has been previously described^23^ (Supplementary Note; Supplementary Fig. 5).

We compared our reconstructed placental transcriptome with a database of tissue-wide reconstructed transcriptomes based on the 9,795 RNA-Seq datasets from GTEx^1^ (CHESS^25^). Among transcripts expressed in ≥90% of the samples and RPKM ≥ 0.1 (i.e., with high confidence), there were 22 and 181 reconstructed placental transcripts classified as ‘within a reference intron’ and ‘potentially novel isoform’, respectively. The 22 ‘within a reference intron’ transcripts were all single-exon transcripts of which 13 transcripts did not overlap with any transcript in the CHESS database. This suggests they may not be present in the 31 non-placental human tissues used to construct the CHESS database (Supplementary Data 17). Of the 181 reconstructed placental transcripts in the ‘potentially novel isoform’ category, 141 completely matched the intron chains annotated in the CHESS database, suggesting they are also present in other tissues. However, interestingly, there were 25 transcripts still classified as ‘potentially novel isoform’ (i.e., matched at least one junction), suggesting they are not recognized in the 31 tissues on which the CHESS database is built (Supplementary Data 17).

We identified two novel leptin transcript variants present in >10% of the samples analyzed and both of these had an altered exon splice site leading to the loss of glutamine at position 49 of leptin (Supplementary Note; Supplementary Fig. 7, Supplementary Data 18–19). These transcripts were more commonly found in placentas from pregnancies complicated by PE. For example, TCONS_00329506 is present in 41 samples and it is 1.4 times more frequent in samples from pregnancies affected by PE (56%) than healthy controls (39%, *P* = 0.0011, chi-squared test).

### Placental circRNAs and their possible function

The placenta contains multiple abundant and common circRNAs. In our study, 3,279 and 679 circRNAs are present in 30% and 90% of the samples respectively. The majority of back-spliced sites are exonic (Supplementary Figure 8a), so we determined whether highly expressed genes, such as *PAPPA* and *PAPPA2*, host more circRNAs. There was a very weak correlation (*r* = 0.059, *P* < 8 × 10^−4^, Supplementary Fig. 8b) between the number of back-spliced reads (i.e., abundance level of circRNAs) and the abundance (i.e., RPKM) of their host genes. In fact, the relationship between the ratio (back-splice:linear-splice) and host gene mRNA abundance is negatively correlated (*r* = −0.185, *P* < 1.5 × 10^−16^, Supplementary Fig. 8c). This suggests those genes hosting many circRNAs are not simply highly ranked due to their transcript abundance and that these circRNAs are not artefacts of the alignment of spliced-reads.

**Fig. 3:**
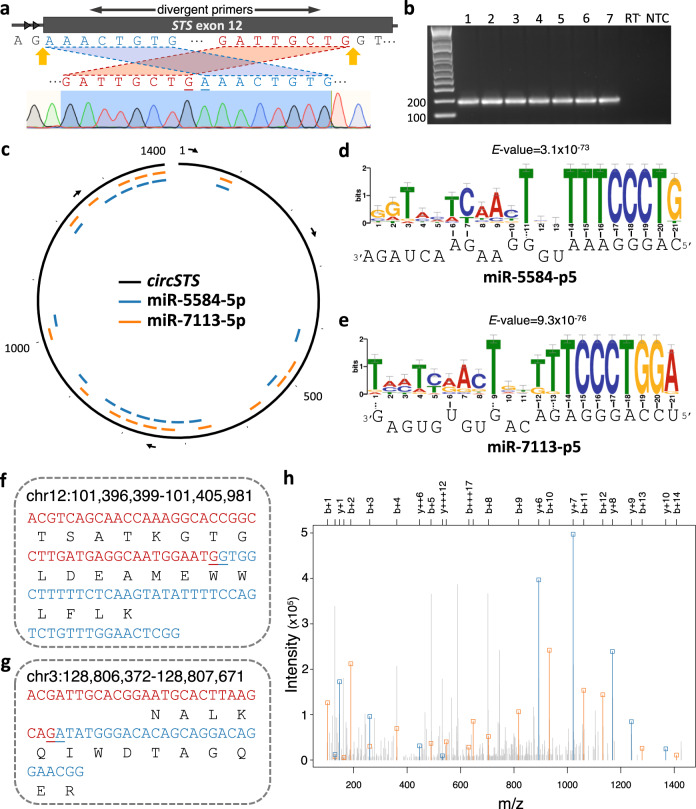
circSTS as a putative miRNA sponge and evidence of peptides translated from circRNAs. **a** The back-spliced positions (indicated in yellow arrows) are within the 12th exon (thick rectangle) of a lincRNA (ENST00000658154) encoded by *STS*. Back-splicing was assayed with divergent primers (top) and confirmed by Sanger sequencing (bottom). The first and last 8-bases, colored in blue (5’) and red (3’) respectively, are flanked by AG/GT (intronic acceptor/donor sites). **b** The expected size of the back-spliced PCR product from circSTS (170 bp) was validated by qPCR from seven placental samples. RT^-^: no reverse transcriptase; NTC: no template control. Source data are provided as a Source Data file. **c** circSTS (chrX:7,514,882-7,516,290) is represented as a black open circle with its putative binding sites for miR-5584-5p (blue) and miR-7113-5p (orange). Its relative base positions are marked for every 100th-base position and 1st, 500th, 1000th, and 1400th positions are numbered. The arrows indicate 5′-to-3′ direction. Sequence motif analyses of miR-5584-5p and miR-7113-p5 are shown in (**d**) and (**e**) respectively, with the corresponding sequence miRNAs shown at the bottom. The size of the sequence logo represents how well the binding base is conserved across the 16 predicted binding regions represented in (**c**). **f**–**g** Peptidomic evidence for translation of two circRNAs. The putative peptides are in black and the bases on either side of the BSJ are colored in red (5′) and blue (3′). The peptides were translated across the BSJ (indicated by underlines) of circRNAs. **h** Mass spectrum corresponding to the peptide sequence of (**f**). On the *x*-axis, the mass/charge ratio of the peptide fragments is shown, with the intensity on the *y*-axis. Peaks that can be attributed to b and *y*-ions (i.e., including the N- and C- termini respectively) of the peptide are indicated with a square and colored in red and blue respectively.

We compared circRNAs in our placental samples with those reported in 20 human tissues (described by Maass et al.^26^ and circBase^27^) and to cancer-based circRNAs (MiOncoCirc^28^). We found 35 circRNAs present in at least 90% of our placental samples, which were not reported by Maass et al. or in circBase (Supplementary Fig. 9a). When we compared the POPS90 dataset with those reported in placental and decidual samples from the Maass et al., there were 563 circRNAs uniquely identified in POPS90 (Supplementary Fig. 9b). There were 172 and 16 circRNAs in the POPS30 and POPS90 datasets respectively (Supplementary Fig. 9c), that were not reported by Maass et al., circBase, and MiOncoCirc. Interestingly, *PAPPA2* hosted the most unreported placental circRNAs (Supplementary Data 20). We experimentally validated six of our predicted circRNAs including one novel and five previously reported (Fig. 3, Supplementary Data 21 and Supplementary Fig. 10).

CDR1as (also known as CiRS-7, chrX:140,783,175-140,784,659) is a circRNA encoded within the antisense strand of the human cerebellar degeneration related protein 1 (*CDR1*) gene and acts as a miRNA sponge which inhibits the actions of miR-7^29,30^. This circRNA harbors 74 miR-7 seed matches of which 63 are conserved in at least one other species^30^. We found CDR1as in 173 of the 295 placental RNA datasets (59%) and it had the highest ratio of circular junction reads to non-circular reads in the placenta (0.93, Supplementary Data 8). We validated the presence of this circRNA in our placental samples by RT-qPCR and confirmed the identity of the back-spliced junction using Sanger sequencing (Supplementary Fig. 10). We used miRanda^31^, and found that CDR1as harbors 67 candidate target sites for miR-7-5p, equivalent to 45 seed matches per kilobase (kb) of CDR1as (Supplementary Note). This was the greatest number of matches we found in miRBase^32^. Using the same parameters and our placental data, we sought other circRNAs harboring multiple target sites for miRNAs. We identified a circRNA (chrX:7,514,882-7,516,290) that has 16 candidate miRNA-binding sites (11 seed matches per kb). This circRNA was detected in 82% of the placental RNA-Seq datasets with a ratio of circular junction read of 0.64. It shares the same exonic start position (chrX:7,514,882) as a lincRNA (ENST00000658154) which is transcribed from the steroid sulfatase (*STS*) gene locus (Fig. 3a). We found that the peaks in the RNA-Seq coverage graph corresponded to the location of back-spice junction (Supplementary Fig. 11). We validated the back-splicing by RT-PCR and Sanger sequencing (Fig. 3a and b). This circRNA (we termed circSTS) is also described in circBase^27^ (http://www.circbase.org/cgi-bin/singlerecord.cgi?id=hsa_circ_0140572) and has been described by Rybak-Wolf et al.^33^ in the mammalian brain. circSTS is predicted to have 16 candidate binding sites for both miR-5584-5p and miR-7113-5p (Fig. 3c), which were rarely detected in our data (<1 read per sample). These two miRNAs share a common 5′-6mer sequence, CAGGGA, and this is complementary to circSTS which is predicted to have a unique 8mer sequence motif ‘TTTCCCTG’ (Fig. 3d and e). These miRNAs have mean predicted binding energies of −19.2 and −25.6 kCal/Mol respectively and these are similar or stronger than that for the predicted binding of MiR-7-5p to CiRS-7 (−19.8 kCal/Mol). We sought additional circRNAs containing more than 10 seed matches per-kb and ranked them based on the number of target sites. The third ranked circRNA (chr19:39,453,356-39,453,521) had only three target sites and a circular junction read to non-circular junction ratio in the placenta of 0.04 (Supplementary Data 8).

Novel protein-coding regions have been identified in translated pseudogenes, ncRNAs, and upstream open reading frames (ORFs)^34^. Since circRNAs have been reported to harbor coding ORFs^35^, we also investigated the possibility that the placental circRNAs could be translated into novel proteins. We used two publicly available tandem mass spectrometry datasets from the draft map of the human proteome^34^ and the proteome and transcriptome abundance atlas^36^. We searched for matches to predicted peptides that span the back-splice junction of the placental circRNAs (see “Methods” for detail) and found peptides uniquely matched to two circRNAs (Fig. 3f-g): (i) chr12:101,396,399-101,405,981 (hsa_circ_0027902) and (ii) chr3:128,806,372-128,807,671 (hsa_circ_0008923). For the peptides shown in Fig. 3f and g there were three and six peptide-spectrum matches (PSMs), respectively. In addition, a shorter peptide (GTGLDEAMEWWLFLK) that was internal to the peptide shown in Fig. 3f also matched the circRNA chr12:101,396,399-101,405,981. This had a higher PSM (*n* = 26) (Supplementary Data 22).

### Network analysis of miRNA and mRNA co-expression

**Fig. 4:**
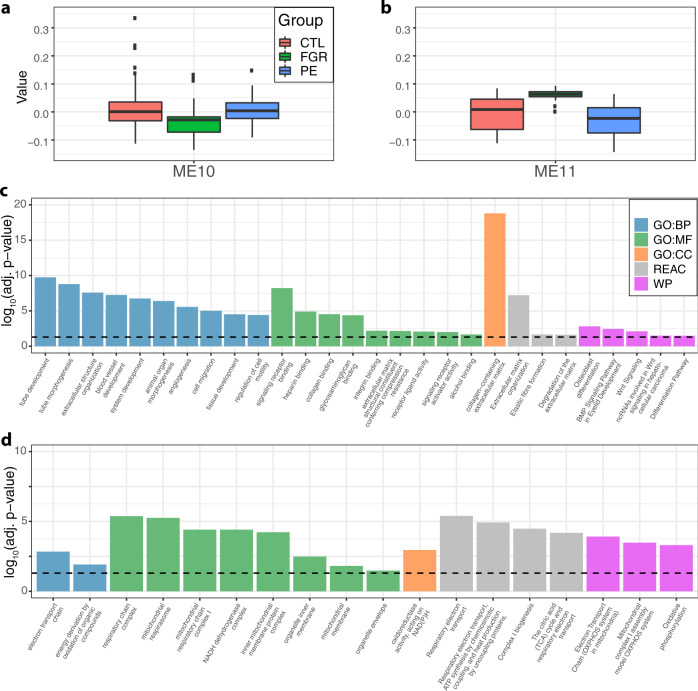
Characterization of miRNA-enriched WGCNA network modules 10 and 11. The eigengene summarizing the transcripts in (**a**) module 10 was associated with FGR (*q*-value = 5.8 × 10^−5^); and (**b**) module 11 was associated with both FGR (*q*-value = 1.8 × 10^−7^; 52 cases and 148 controls) and PE (*q*-value = 1.1 × 10^−3^; 88 cases and 148 controls). The *q*-values were calculated using the ‘qvalue’ Bioconductor package to control the local false discovery rate at the 0.05 level. Significantly overrepresented Gene Ontology (GO, biological process BP; molecular function MF; and cellular component CC), Reactome (REAC), and WikiPathways (WP) terms are shown for (c) module 10; and (d) module 11. For clarity, only GO terms with a shortest root-to-node path of length 4 are shown, and at most, the top 10 hits within each sub-ontology are shown. The enrichment analyses were performed using the R interface to g:Profiler, and adopting the g:SCS correction to control for multiple testing with threshold *P* = 0.05. All statistical tests were two-sided. Full results are provided in Supplementary Data 28–74. In (**a** and **b**), the boxes show the median and the lower and upper quartiles. The whiskers extend from the lowest observed point still within 1.5 times IQR (the interquartile range) of the lower quartile, to the highest observed point still within 1.5 times IQR of the upper quartile. All points beyond the whiskers are plotted as outliers.

To systematically investigate miRNA-mRNA interactions, we performed Weighted Gene Co-expression Network Analysis (WGCNA)^37^, which has previously been used to identify miRNA and mRNA co-expression networks^38^. We identified 22 network modules of highly correlated transcripts, of which two (module 10, comprising 397 mRNA and 40 miRNA; and module 11, comprising 77 mRNA and 357 miRNA) were significantly enriched for miRNAs (Fisher’s exact test, Bonferroni-adjusted *P* < 0.05). Using eigengene network analysis, we found that the eigengenes for both modules were significantly associated with FGR, and that module 11 eigengene was also associated with PE (Fig. 4a and b; see also Supplementary Data 28–74, which shows that several other module eigengenes are also associated with PE and/or FGR). GO, Reactome, and WikiPathways enrichment analyses identified overrepresented terms associated with tube formation and angiogenesis for module 10 (Fig. 4c); and with the mitochondria and respiratory chain for module 11 (Fig. 4d). Module 10 was also enriched for known targets of the miRNAs it contained (11/397 mRNA, Bonferroni-adjusted *P* = 0.0053). These targets had overrepresented GO terms associated with cell surface receptor signaling and regulation of cell communication (Supplementary Data 28–74).

### Correlations between placental mRNAs and serum metabolites

We recently reported a strong association between the ratio of four circulating maternal metabolites and FGR^39^. We therefore calculated the Spearman correlations between these four metabolites in the samples collected at 36 weeks of gestation (wkGA) and the 24,611 transcripts described in this study. We found that 60 transcripts had correlations with at least one of the four metabolites with Spearman’s *rho* ≤ −0.4 or ≥0.4 (Supplementary Fig. 12 and Supplementary Data 23). For 1-(1-enyl-stearoyl)-2-oleoyl-GPC and 1,5-anhydroglucitol there were very few transcripts for which *P*_adj_ ≤ 0.05 (4 and 0 respectively). For 5alpha-androstan-3alpha,17alpha-diol disulfate and N1,N12-diacetylspermine there were 34 and 54 transcripts respectively. Analysis of the GO terms associated with these transcripts did not show any significant enrichment.

For completeness we also correlated all 1185 metabolites for which we have data and the 24,611 transcripts. We identified 213 transcripts and 54 metabolites for which there was at least one correlation where *rho* ≤ −0.5 or ≥0.5. The heatmap showing the clustering of these is shown in Supplementary Fig. 13 with the correlations and adjusted *P* values in Supplementary Data 24–25. Cluster 3 had multiple highly significant correlations (203, with an absolute value of *rho* ≥ 0.5 and *P* < 1.8 × 10^−6^) including a group of proline-related metabolites (4-hydroxyproline, prolyl-hydroxyproline, and 4-hydroxyglutamate). There are 22 transcripts with highly significant positive correlations to these metabolites (*P*_adj_ < 0.0001) and 11 of these are annotated with GO terms. The GO biological process term “renal system vasculature morphogenesis” (GO:0061438) was enriched (<100 fold, *P*_adj_ 0.045). Transcripts with strong negative correlations did not show any enrichment.

### Differential expression in complicated pregnancies

Having observed potentially novel reconstructed transcript isoforms as well as circRNAs and small RNAs, we investigated transcripts dysregulated in the placenta samples from pregnancies affected by PE (*n* = 82) and FGR (*n* = 40) compared to their matched controls (see “Methods”). Using DESeq2 with a *P*_adj_ value cut-off <0.05, we found 4.4% of transcripts differentially regulated in PE, and 1.5% in FGR (Table 1). Most differentially regulated transcripts were either protein-coding or lincRNAs, but there were also 43 novel small-RNAs, 12 miRNAs, and 2 circRNAs. Of those, the circRNA “chr19:53,687,886-53,694,145” was present in all the RNA-Seq samples and it overlaps with following two miRNA genes: *MIR1283-1* (chr19:53,688,481-53,688,567) and *MIR520A* (chr19:53,690,881-53,690,965). This circRNA was validated by qPCR assay and its back-spliced junction was confirmed by Sanger sequencing (Supplementary Fig. 10).

**Table 1:** Differentially regulated placental transcripts in preeclampsia and fetal growth restriction.

Type of transcript	Number of available transcripts		Number of differentially regulated transcripts										
	Annotated or predicted	Filtered^a^	*P* value approach^b^			Fold change approach^c^							
						Top 5%				Top 3%			
			PE	FGR	Shared	PE & FGR each	Shared by PE and FGR			PE & FGR each	Shared by PE and FGR		
							Same direction	Opposite direction	*P* value^d^		Same direction	Opposite direction	*P* value^d^
Protein-coding	19,808	15,257	785	32	3	250	92	3	30.9 × 10^−62^	150	57	1	20.2 × 10^−52^
Novel protein-coding	2783	2774	157	1	0	45	19	0	60.8 × 10^−16^	27	11	0	70.3 × 10^−12^
lincRNA	7672	1741	28	2	0	29	11	1	80.3 × 10^−9^	17	4	0	80.3 × 10^−4^
Novel lincRNA	133	131	6	0	0	7	3	0	30.0 × 10^−3^	4	2	0	40.1 × 10^−3^
circRNA	3279	1879	2	0	0	31	9	0	20.6 × 10^−6^	18	6	0	20.2 × 10^−6^
miRNA	2588	615	12	0	0	10	2	0	00.075	6	0	0	>0.99
Novel miRNA	141	26	0	0	0	1	0	0	>0.99	1	0	0	>0.99
piRNA	32,235	286	0	0	0	5	1	2	00.24	3	0	1	>0.99
sncRNA	2874	177	2	0	0	3	0	0	>0.99	2	0	0	>0.99
Novel small-RNA	18,511	706	43	0	0	12	1	0	00.42	7	1	0	00.16

^a^Tanscripts passing following filters: (1) RPM (Reads Per Million mapped reads) ≥0.2 for non-circRNA; count ≥ 5 for circRNA, (2) present in >10% of the cohort, (3) found in both the FGR and PE cohorts, and (4) genes not on chrY and chrMT.

^b^*P* values were adjusted (Benjamini–Hochberg correction method at false discovery rate (FDR) ≤ 5%) based on the original two-sided *P* values reported from DESeq2.

^c^Fold changes were obtained from bootstrap sampling of cases and controls. The most abundant transcripts were used: the 5000 most abundant protein-coding transcripts were selected from 15,257 transcripts passing the filters described above and the same fraction (i.e., 5000/15257) was selected from the other transcript types.

^d^Based on the Fisher’s exact test (two-sided).

Lists of differentially regulated transcripts in this table are available at https://www.obgyn.cam.ac.uk/placentome/.

Using a conventional *P* value-based approach, only three transcripts, all of which were protein-coding (*FSTL3*, *PNCK*, and *DIO2)* were dysregulated in both PE and FGR. However, to further investigate the differentially regulated transcripts, we repeated the comparison using an alternative approach to calculate the fold change. We bootstrap sampled the data set to generate 82 and 40 pairs (equal to the number of original PE or FGR pairs) 10,000 times with replacement (see “Methods” for details). We ranked transcripts by their average fold change relative to the controls and selected highly ranked transcripts in PE and FGR. Finally, we counted how many were shared between the two conditions. PE and FGR are both heterogeneous conditions and this approach allows transcripts of interest to be identified without penalizing those which have greater variability—i.e., those that might differ between currently unknown subtypes of the conditions. In addition to protein-coding transcripts, we found that long non-coding and circRNA transcripts were dysregulated in PE and FGR (Table 1), which suggests similarities in the placental dysfunction underlying both conditions. However, using the same criteria only a few small-RNA transcripts were shared between the two conditions. We performed Gene Ontology analysis using the 92 protein-coding transcripts which changed in the same direction in PE and FGR (Supplementary Data 26). The majority of overrepresented GO terms are associated with endocrine regulation, such as hormone response, transport and secretion, response to reactive oxygen species and metal ion, and regulation of receptor protein kinase signaling pathway.

We further analyzed differentially regulated protein-coding transcripts by sub-dividing FGR cases according to the following clinical characteristics (defined in detail in the “Methods” section): (1) FGR infant whose mother experienced any hypertensive disorder, (2) FGR infant from a normotensive mother who showed abnormal (low) PAPP-A (Pregnancy Associated Plasma Protein-A) level, (3) FGR infant showing abnormal fetal growth velocity from a normotensive mother, (4) FGR infant from a normotensive mother who showed abnormal uterine blood flow, and (5) FGR infant from a normotensive mother who showed abnormal umbilical blood flow. We found most of the dysregulated transcripts were unique to each FGR sub-category and there were no commonly dysregulated transcripts shared among the five sub-categories based on the *P* value approach (Supplementary Fig. 14; Supplementary Data 27).

**Fig. 5:**
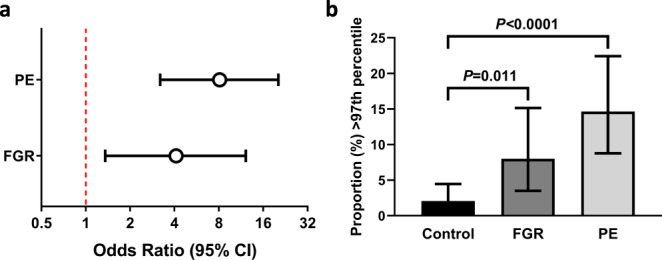
Circulating maternal levels of FSTL3 at 36 weeks of gestation and adverse pregnancy outcome. **a** The association between elevated serum FSTL3 (>97th percentile) and PE or FGR (see “Methods” for definitions), expressed as odds ratios and 95% confidence intervals (95% CI); and **b** the proportion of samples above 97th percentile (95% CI). This analysis is based on 495 samples (289 controls and 206 cases of which 106 had PE only, 90 had FGR only and 10 had both PE and FGR). The *P* values were calculated using Fisher’s exact test (two-sided) based on the dichotomized percentile matrix. The exact P value between Control and PE is 4.6 × 10^−6^ (**b**).

Of the three transcripts detected as differentially expressed in both PE and FGR (all categories) using both the *P* value and fold-change approaches, only *FSTL3* encodes a secreted protein. We therefore measured circulating FSTL3 in maternal serum collected at 36 weeks of gestation (case-cohort design, *n* = 495). In the random sub-cohort (*n* = 334), the median [IQR] concentration of FSTL3 at 36 wkGA was 19.4 ng/ml [14.5–25.6]. Multiples of the median (MoM) of the concentrations were calculated and subsequently corrected for maternal and sample characteristics (see “Methods”). Corrected maternal FSTL3 levels >97th percentile (3.11 MoMs) were associated with an increased risk of PE (OR = 8.1 [95%CI 3.2–20.3]) and FGR (OR = 4.1 [95%CI 1.4–12.2], Fig. 5a). The proportion of samples with FSTL3 levels >97th percentile was 2.1% in controls, 8.0% in FGR, and 14.7% in PE (Fig. 5b).

## Discussion

We have comprehensively analyzed the placental transcriptome using high coverage RNA-Seq of rRNA-depleted samples (~100 M reads in 302 placental samples). We have also analyzed the small RNA population in a similar number of samples.

The placental transcriptome is skewed with several transcripts having very high abundance. Nine transcripts are present at >2000 TPM (Transcript Per Million mapped reads) and seven of these are placental specific. Many of these encode secreted proteins which are released into the maternal circulation. For example, CSH1 (Chorionic Somatomammotropin Hormone 1, or placental lactogen, TPM > 70,000), all the protein-coding members of the PSG family, PAPPA, PAPPA2, and the hormones Kisspeptin and CRH. This preponderance of extremely abundant transcripts encoding secreted proteins may be a reflection of the conflict between the maternal and fetal genomes and the divergent priorities of mother and fetus. Mothers can conserve resources for the growth of later offspring or invest more in the current pregnancy, in contrast the fetus will maximize its own growth and development^40^. An additional consequence of the high levels of protein translation and secretion is that the placenta, like the pancreas, is susceptible to endoplasmic reticulum stress and this is implicated in adverse pregnancy outcome^41–44^.

There is also pronounced skewing in the relative abundance of the annotated mi- and pi- RNAs. piRNAs primarily function to protect the genome of germ cells from the mobilization of transposable elements which are silenced by both transcriptional and post-transcriptional gene silencing. However, there is growing interest in their possible function in somatic cells and cancer^45^. By definition piRNAs require PIWI family members to act^46^ and it has been suggested that the piRNA databases contain a low level of “contaminating” RNAs that are not true piRNAs^47^. A proportion of these RNAs are fragments of other ncRNA, particularly tRNAs^48^. The sequencing of tRNAs requires specialized methods and analyses^49,50^ due to their numerous posttranscriptional modifications, strong secondary structure, and high copy number. Although some recent studies report quantification of tRNAs from small RNA-Seq data^51,52^, they are poorly sequenced and generally not reported in papers describing RNA-Seq data. Nonetheless, this would be an area worthy of future study. However, fragments from tRNAs can act directly to inhibit translation and are implicated in multiple diseases^53,54^. They have recently been catalogued and are available in an accessible database (MINTbase^55^). We noted that a small number of annotated piRNAs were extremely abundant in the placenta (8 piRNAs >20,000 RPM, reads per million mapped reads) and several of these are encoded by the mitochondrial genome. The most abundant annotated placental mitochondrial piRNAs (piR-hsa-26684, piR-hsa-26685, piR-hsa-26686, >36,000 RPM) all overlap with a mitochondrially encoded tRNA. These are well described in MINTbase and have been detected in many hundreds of the samples in the Cancer Genome Atlas datasets (TCGA) with abundances of between 100 and 500 RPM. tRNA fragments play a role in regulating genes in the Wnt signaling pathway in cancer cells^56^. Somatic PIWIL4 (also known as HIWI2) preferentially binds piRNA-like tRNA-derived fragments^57^ and as PIWIL4 mRNA is present in the placenta the actions of some of the piRNA-like RNAs may be mediated by PIWIL4. However, it is likely that these piRNA-like tRNA fragments have several different mechanisms of action^58,59^. As tRNA fragments are released from the placenta in microvesicles it is possible that they could also have maternal effects^60^.

circRNAs have a variety of functions but these have only been investigated in detail in a few cases as described in a recent review^61^. The best-characterized function is their action as miRNA sponges^29,30^. Using stringent criteria that identify a known miRNA sponge, we identified an additional circRNA (circSTS; chrX:7,514,882-7,516,290) that is a candidate for functioning as an miRNA sponge. While the frequency and thermodynamic characteristics of the predicted binding sites suggest that miR5584-5p and miR-7113-5p function could be modified by interaction with circSTS these predicted interactions should be treated with caution. The possible interactions of these miRNAs depend on the complex stoichiometry of all their possible binding partners. Furthermore, the functional consequences of miRNA-circRNA interaction remain poorly understood even in cases where there is compelling evidence for binding, such as for CDR1as. Recent evidence suggests that CDR1as might stabilize rather than titrate miR-7-5p^62^ and that correlations between circRNA and mRNA level, which might be interpreted as evidence of a functional circRNA sponge, can be explained by the cellular composition of a tissue sample^63^.

CircRNAs have been reported to harbor coding ORFs^35^ and we found strong evidence for translation of these in the placenta. Other known functions include acting as protein sponges; forming scaffolds to mediate complex formation between enzymes and substrates; recruiting proteins to specific locations (on DNA for example) and enhancing protein function^61^. However, the majority of the placental circRNAs we identified have no recognizable function. Nonetheless, given that some are present in almost every sample we analyzed and are more abundant than the linear transcript containing the same exons there is considerable scope for the discovery of novel regulatory mechanisms. Furthermore, circRNAs have been catalogued in cancer and the authors suggested this was a “valuable resource for the development of diagnostic or therapeutic targets”^28^.

PE and FGR can both be manifestations of placental dysfunction and have some shared placental histopathological abnormalities^64,65^. However, there is no clear mechanistic understanding of why each condition is manifested in isolation or in combination. A distinction is often made between early- and late-onset PE and this is attributed to either placental or maternal factors. However, it seems more likely that it is a matter of degree and timing of the placental insult and maternal sensitivity with genetics and the environment influencing each of these^44,66^. When we used a bootstrapped fold-change analysis to allow for heterogeneity within the diagnostic groups, we found highly significant overlaps between the transcripts dysregulated in PE and FGR for all the classes of long RNAs we investigated. However, the majority of these were missed using a conventional *P* value-based approach. These findings underline the fact that both conditions may be the end result of multiple different pathways. The bootstrapping analysis indicates likely overlap in these pathways but even larger-scale analysis of optimally phenotyped samples and/or analysis using other “omic” methods may be required to understand the common and divergent pathways leading to these outcomes.

*FSTL3* mRNA is present in many tissues and the placental level is ranked 13th of the 50 tissues examined here. Thus, tissues other than the placenta will likely contribute to the serum FSTL3. However, the levels we report in pregnant women at 36 wkGA (median [IQR] 19.4 ng/ml; [14.5–25.6]) were approximately three times greater than reported in non-pregnant subjects (6.1 ng/ml, 5.3–7.2)^67^. FSTL3 forms high-affinity inhibitory complexes with TGF-*β* family members, most notably activin A and myostatin (also known as GDF8). Administration of lipopolysaccharide (an activator of the innate immune system) increased the circulating levels of FSTL3 of non-pregnant subjects approximately fourfold^68^. Interestingly, 15% of women with PE and 8% of women with FGR had FSTL3 levels in excess of 61.4 ng/ml (97th percentile, uncorrected value). These observations are consistent with the hypothesis that PE is associated with maternal systemic inflammation in a subset of cases^44^.

The investigation of possible correlations between circulating metabolites and placental transcripts indicated that there are some strong and highly significant relationships. However, only one subset of transcripts and metabolites showed enrichment of a GO biological process. This was related to kidney vascular morphogenesis and the two transcripts associated with this term (*NOTCH3* and *PDGFRB*) together with *HIF3A* (also present in the 22 highly correlated transcript set) may play a role in regulating placental blood vessel growth and/or maintenance. We have previously reported that elevation of serum level of 4-hydroxyglutamate is associated with PE^39^ but the functional relationships between the correlated metabolites and transcripts are not known and is an area for further study.

One of the strengths of this study is that we sampled more than 300 well-phenotyped placentas and sequenced to high depth. As we analyzed strand-specific total (not poly-A^+^ selected) RNA we were able to characterize a large number of circRNAs in the placenta. There are several gene clusters on chromosome 19 with largely or wholly placental-specific expression—the *PSG* family and the C19MC. Both these loci are undergoing rapid evolution, the *PSG*s appear only to be present in mammals with hemochorial placentation and the C19MC is restricted to primates^69^ and are imprinted with expression only from the paternal allele. Other genes of key reproductive relevance—the killer immunoglobulin receptor (*KIR*) genes which in part mediate the interaction between uterine natural killer cells and trophoblast cells are also clustered on chromosome 19. These two also are evolving rapidly^70^. We identified an additional cluster of circRNAs on this chromosome although the function of these is largely unknown. This comprehensive analysis revealed the marked skewing in the abundance of placental-specific transcripts encoding secreted proteins and piRNAs. Thus, the human placental transcriptome has several unique features which may reflect the diversity and rapid evolution of the placenta.

## Methods

### Placental samples

All the samples were obtained from the POP study, a prospective cohort study of nulliparous women attending the Rosie Hospital, Cambridge (UK) for their dating ultrasound scan between 14 January 2008 and 31 July 2012. The study has been previously described in detail^12–14^. Ethical approval for the study was given by the Cambridgeshire 2 Research Ethics Committee (reference number 07/H0308/163) of the NHS Health Research Authority and all participants provided written informed consent. Cases of PE were defined on the basis of the 2013 ACOG criteria^71^ and cases of FGR had a customized birth weight <5th percentile^72^. Controls (CTL) were defined as pregnancies resulting in a live-born infant with a birth weight percentile in the normal range (20–80th percentile^72^) with no evidence of slowing in fetal growth trajectories, and with no evidence of hypertension at booking and during pregnancy, PE, hemolysis/elevated liver enzymes/low platelet (HELLP) syndrome, gestational diabetes or diabetes mellitus type I or type II or other obstetric complications. A total of 302 unique placental samples were considered in this study, among which there were 94 PE, 56 FGR, and 155 control samples (three samples were classified as both PE and FGR, Supplementary Fig. 1).

### RNA extraction and library preparation

Placental biopsies were collected within 30 min of birth and flash frozen in RNA*later* (ThermoFisher). For each biopsy, total placental RNA was extracted from ~5 mg of tissue using the “mirVana miRNA Isolation Kit” (Ambion) followed by DNase treatment (“DNA-free DNA Removal Kit”, Ambion). RNA quality was assessed with the Agilent Bioanalyzer and all the samples with RIN values ≥7.0 were used in the downstream experiments. Total RNA-libraries were prepared from 300 to 500 ng of total placental RNA with the TruSeq Stranded Total RNA Library Prep Kit with Ribo-Zero Human/Mouse/Rat (Illumina), pooled and sequenced (single-end, 125 bp) using a Single End V4 cluster kit and Illumina HiSeq2500 and HiSeq4000 instruments. Small RNA-libraries were prepared from 150 ng of total placental RNA with the NEBNext Multiplex Small RNA Library Prep Kit for Illumina (New England Biolabs) and concentrated using the “QIAquick PCR purification kit” (Qiagen). Paired libraries were combined and size selected using the Pippin Prep and 3% Agarose Gel Cassette with marker F (Sage Science), pooled, and sequenced (single-end, 50 bp) using a Single End V4 cluster kit and HiSeq4000 instrument.

### Quality control of sequencing data

We generated 324 total RNA-Seq data sets (single-end 125 bp) from 302 unique placental biopsies from the POP study cohort^12–14^, including 94 PE, 56 FGR, and 155 control samples—three cases of samples were classified as both PE and FGR (Supplementary Fig. 1). Of the 302 biopsies, 22 were sequenced twice (19 control samples and 3 aforementioned samples classified as both PE and FGR cases) and the replicates were merged for transcript quantification. However, for transcriptome reconstruction (explained later), only one of the replicates was chosen based on the following criteria: recently sequenced replicates of planned caesarean section (three samples); otherwise replicates sequenced together with FGR cases (16 samples). Supplementary Data 2 shows the number of raw reads, mapped reads and the mapping statistics. Out of a total of 302 placental biopsies, three samples were excluded due to the presence of decidual contamination (Supplementary Fig. 1). Using *DESeq2* (v.1.18.1)^73^, we calculated Cook’s distance^74^ to identify gene-count outliers for all 324 RNA-Seq datasets. We identified seven genes of recurrently highly ranked Cook’s distance (a large value of Cook’s distance indicates an outlier count) from three samples that had 20-fold higher transcript levels compared to the median value: insulin-like growth factor binding protein 1 (*IGFBP1*), osteomodulin (*OMD*), prolactin (*PRL*), retinol-binding protein 4 (*RBP4*), *KIAA1644*, RAR related orphan receptor B (*RORB*) and chordin like 1 (*CHRDL1*). Transcripts from these seven genes are reported as highly abundant in the decidua^75,76^. A further sample was excluded as it showed a low mapping ratio (~50%) in GRCh37.

### Total RNA-Seq data processing

For total RNA-Seq datasets, adaptor sequences and poor-quality bases were trimmed using *Trim Galore!* (v0.4.0)^77^, which uses *cutadapt* (v1.8.1)^78^ internally, using the following parameters:

*--adaptor AGATCGGAAGAGCACACGTCTGAACTCCAGTCAC --quality 5 --stringency 5*.

Quality-assured trimmed reads were mapped to the GRCh38 version of human genome reference using *TopHat2* (v2.0.12)^79^, a splice-aware mapper built on top of *Bowtie2* short-read aligner (v2.2.3.0)^80^. We applied the so-called two-pass (or two-scan) alignment protocol to rescue unmapped reads from the initial mapping step which was executed with the following parameters:

*--library-type fr-firststrand --max-multihits 10 --prefilter-multihits --transcriptome-index* = *”Ensembl.v82.gtf.indexed.directory”*.

In the second mapping of *TopHat2*, previously unmapped reads were re-aligned toward the exon-intron junctions detected in the first mapping without the guide of genome reference using the following parameters:

*--raw-juncs “sample.wide.merged.junction.bed” --no-novel-junc --library-type fr-firststrand --max-multihits 10*.

30% of the final mapped reads were rescued from the second mapping step (Supplementary Data 2). For each sample, the initial and second mapped reads were merged by *samtools* (v1.2-24-g016c62b)^81^. We obtained a total of ~33 billion reads (a median sequencing depth of 101 million reads per sample), of which ~30.8 billion reads (i.e., 92.9% mapping efficiency) were mapped to the GRCh38 version of the human reference genome sequence (Supplementary Data 2). There was no bias in the mapping efficiency by different pregnancy outcomes and batches (Supplementary Fig. 1).

### Small RNA-Seq data processing

We also generated 328 small RNA-Seq datasets, producing ~6.6 billion reads in total, equivalent to a median sequencing depth of ~20 million per sample. The mapping efficiency for small RNAs was 75.7% (Supplementary Data 3). Firstly, adaptor sequences and poor-quality bases were trimmed using *cutadapt* (v1.8.1)^78^ using the following parameters:

*--trim-only --minimum-length* = *15 --quality-cutoff* = *20 --overlap* = *8 -a NEB3PrimeAdaptor* = *AGATCGGAAGAGCACACGTCT -g NEB5PrimeAdaptor* = *GTTCAGAGTTCTACAGTCCGACGATC*.

Quality-assured trimmed short reads were mapped to the GRCh38 version of the human genome reference sequence using *mapper.pl* script of *miRDeep2* (v2.0.0.7)^82^, which uses *bowtie* (v.1.1.2)^83^ internally, with the following parameters: *-n -c -m -j*.

Then the mapped read file (in. arf format) and the processed read file (i.e., collapsed FASTA file), together with the known and homologous sequences of human miRNAs from *mirBase* (v21)^32^, were run through *miRDeep2.pl* script which is the core part of *miRDeep2*. Known precursor and mature miRNAs were further processed from the quantification outputs (in.csv format) of *miRDeep2* using the following custom *awk* commands:

*awk ‘BEGIN{OFS* = *“\t”}!/^#/{Precursor[$3]+=$2}END{for(p in Precursor)print p,Precursor[p]}’* and *awk ‘BEGIN{OFS* = *“\t”}!/^#/{Mature[$1]+=$2}END{for(m in Mature)print m,Mature[m]}’*.

### Novel miRNA detection

Novel miRNAs were predicted by the same pipeline of *miRDeep2* and their loci were merged if they overlap at least 1 bp using *bedtools* with the following command:

*bedtools merge -s -c 5,6 -o mean,distinct*

We used two additional miRNA prediction tools, *sRNAbench* and *miRge2.0* with the following parameters:

*java -Xmx12g -jar sRNAbench.jar input* = *$FQ_FILE output* = *$OUTPUT_DIR \*

*dbPath* = *$SRNA_DBPATH species* = *genome microRNA* = *hsa libs* = *$PIRBASE_V1_FASTA \*

*plotLibs* = *true predict* = *true \*

*minReadLength* = *19 maxReadLength* = *25* *mm* = *0 alignType* = *v minRC* = *2 p* = *$CORE_NUM*

*miRge2.0 predict -s $FQ_FILE -o $PROJECT_DIR -sp human -d miRBase \*

*-lib $MIRGE_LIB -pb $BOWTIE_DIR -ps $SAMTOOLS_DIR \*

*-pr $RNA_FOLD_DIR -minl 19 -maxl 25 -cpu $CORE_NUM*

From the novel miRNAs predicted by miRDeep2, we filtered them out if they were not supported by sRNAbench or miRge2.0 with at least 30% overlap reciprocally:

*bedtools intersect -a $NOVEL_MIRNA_MIRDEEP2.bed \*

*-b $NOVEL_MIRNA_sRNAbench.bed \*

*-b $NOVEL_MIRNA_miRge.bed -s -u -wa -f 0.3 -r*

### piRNA detection

To detect and quantify piRNAs, we scanned the mapping result files of *miRDeep2* and filtered out reads that overlap at least 1 bp with the mature miRNAs annotated by *mirBase* (v21). We confirmed that the size of those ‘filtered’ reads (i.e., mapped reads without any overlaps with miRNAs) was peaked at 28 nucleotides, which is very close to the median size of known piRNAs (Supplementary Fig. 6). Those ‘filtered’ reads were used for quantification if their genomic coordinates overlapped with at least 30% of piRNAs defined from *piRBase* (v1.0)^84^. As *piRBase* v1 was built on a different version of human genome reference (hg19), the genomic coordinates were converted to GRCh38 using *liftover* tool^85^, then *parse_mappings.pl* script of *miRDeep2* was used, followed by a series of chained commands using *awk*, *sort*, and *bedtools* (v2.20.1)^86^ as follows:

*parse_mappings.pl $ARF_FILE -a 0 -i 3 \*

|*awk ‘BEGIN{OFS* = *“\t”}{if($6* = *=“MT”){$6* = *“M”}; start* = *$8-1; split($1,seq,”_x”); print “chr”$6,start,$9,$1,seq[2],$11}’ \*

|*bedtools intersect -a stdin -b $MIRBASE_V21.bed -s -v \*

|*intersectBed -a $PIRBASE_V1.bed -b stdin -s -wao -f 0.3 \*

|*awk ‘BEGIN{OFS* = *“\t”}{if($11* < *1)cnt* = *0;else cnt* = *$11; piRNA[$4]+=cnt}END{for(p in piRNA)print p,piRNA[p]}’ \*

|*sort -k1,1* *V*

### sncRNA detection

sncRNAs include the following three types of small RNAs annotated from Ensembl v82: snoRNA (small nucleolar RNA), snRNA (small nuclear RNA) and sRNA (small RNA). Firstly, we scanned the mapped read files of *miRDeep2* and filtered out reads that overlap at least 1 bp with any from the following sources: (1) known precursor and mature miRNAs annotated by *mirBase*, (2) *piRNAs* annotated from *piRBase*, and (3) tRNAs annotated from Gencode v37. Then, we quantified sncRNAs if the remaining reads overlap sncRNA loci at least 30%. It can be summarized with the following pseudo-code:

*parse_mappings.pl $ARF_FILE -a 0 -i 3 \*

|*awk ‘BEGIN{OFS* = *“\t”}{if($6* = *=“MT”){$6* = *“M”}; start* = *$8-1; split($1,seq,”_x”); print “chr”$6,start,$9,$1,seq[2],$11}’ \*

|*bedtools intersect -a stdin -b $MIRBASE_V21.bed $PIRBASE_V1.bed $GENCODE_tRNA.bed -s -v \*

|*intersectBed -a $sncRNA.bed -b stdin -s -wao -f 0.3 \*

|*awk ‘BEGIN{FS* = *“\t”}{if($14* < *1)cnt* = *0;else cnt* = *$14; split($9,foo,”;”); split(foo[1],bar,“gene_id “); ensg* = *substr(bar[2],2,15); ID[ensg]+=cnt}END{for(id in ID)print id,ID[id]}’*

|*sort -k1,1* *V*

### Novel small-RNA detection

We defined novel small-RNAs based on those high-depth (i.e., >10x) mapped reads yet remained unannotated from various sources. To identify novel small-RNA loci, we scanned the same mapped reads files mentioned earlier and filtered out reads that overlap with any from the following sources: (1) known precursor and mature miRNAs annotated by *mirBase*, (2) novel miRNAs predicted by *miRDeep2,* (3) *piRNAs* annotated from *piRBase*, and (4) any exonic regions defined by Ensembl gene model:

*parse_mappings.pl $ARF_FILE -a 0 -i 3 \*

|*awk ‘BEGIN{OFS* = *“\t”}{start* = *$8-1; split($1,seq,”_x”); print “chr”$6,start,$9,$1,seq[2],$11}’ \*

|*bedtools intersect -a stdin -b $MIRBASE_V21.bed $NOVEL_MIRNA.bed $PIRBASE_V1.bed $ENSEMBL_EXON.bed -s -v \*

|*sort -k1,1* *V -k2,2n*

The remaining reads were further filtered out if their depth of coverage was <10x per sample and their distinct loci were identified across all the samples. The final loci of novel small-RNAs were merged if they overlap at least 1 bp and their abundances were measured using the original mapped reads. Likewise, reads overlapping at least 30% of the novel small-RNA loci were used:

*parse_mappings.pl $ARF_FILE -a 0 -i 3 \*

|*awk ‘BEGIN{OFS* = *“\t”}{start* = *$8-1; split($1,seq,”_x”); print $6,start,$9,$1,seq[2],$11}’ \*

|*intersectBed -a $NOVEL_SMALLRNA.bed -b stdin -s -wao -f 0.3 \*

|*sort -k1,1 -k2,2n -k3,3n -k4,4 -k6,6 \*

|*groupBy -i stdin -grp 1,2,3,4,6 -c 11 -o sum \*

|*awk ‘BEGIN{OFS* = *“\t”}{if($6* < *1)cnt* = *0;else cnt* = *$6; print $1”:”$2”:”$3”:”$5,cnt}’*

### Case-control matching for differential expression analysis

To select the PE-control pairs, we identified 95 PE cases that had a placental sample collected within 30 min of delivery and we used a custom Python script to match the cases to appropriate controls. Matching was performed as closely as possible on seven characteristics in the following order of importance: (1) presence of labor, (2) gestational age, (3) fetal sex, (4) caesarean section, (5) smoking status, (6) maternal body mass index (BMI) and (7) maternal age. Sequencing failed for 1 pair, and therefore we had 94 matched pairs at the start of the analysis (Supplemental Fig. 1). Subsequently, 12 pairs were excluded due to the presence of one or more of the following issues: (1) “batch effect” during the RNA extraction (3 pairs), (2) decidual contamination of the tissue biopsies (2 pairs), (3) and/or fetal sex mismatch in the case-control pair (7 pairs). Samples of batch effect were detected by plotting the normalized RNA-Seq data on a multidimensional scaling plot (MDS). These samples clustered separately on the MDS plot and were identified to be from the same RNA extraction batch. We included 82 PE case-control pairs in the final differentially expressed gene analysis presented in Table 1. To select the FGR-control pairs, we identified 58 FGR cases and 415 eligible healthy controls who had a placental sample collected within 30 min of delivery. Case-control matching was performed as closely as possible on seven characteristics in the following order of importance: (1) gestational age, (2) fetal sex, (3) mode of delivery (vaginal, intrapartum caesarean, or pre-labor caesarean), (4) maternal smoking, (5) placental collection time, (6) maternal BMI, and (7) maternal age. A script was written in Stata version 13.1 to perform the matching. We were unable to find an adequate match for one extremely preterm case and two matched pairs were excluded due to low RNA quality or low mapped reads. At the start of the analysis we had 55 matched pairs. Subsequently, one pair was excluded due to decidual contamination and five pairs were excluded due to fetal sex mismatch between the case and the control sample. From the 49 remaining pairs, we further excluded pairs where the case also had PE (*n* = 5), essential hypertension (*n* = 3) or gestational hypertension (*n* = 1). We included 40 FGR case-control pairs in the final analysis. Both PE and FGR case-control matching are summarized in Supplementary Fig. 1. There were 10 control samples used to match both PE and FGR cases.

### Sub-categories of FGR cases

Cases of FGR were further sub-categorized according to the following clinical characteristics: (1) maternal hypertensive disorder including PE or essential hypertension or gestational hypertension^87^, (2) abnormal maternal PAPP-A level defined as the lowest decile of the 12-week PAPPA multiple of the median (MoM) corrected for gestational age and maternal weight at measurement^88^, (3) abnormal fetal growth velocity defined as the lowest decile of the abdominal circumference growth velocity z score^13^ from the 20 wkGA scan to the last research scan (36 wkGA scan but 28 wkGA scan if 36 wkGA scan result was missing), (4) abnormal uterine artery Doppler defined as pulsatility index at 20 wkGA scan in the top decile or bilateral notch in the 20 wkGA scan (see Sovio et al.^13^ for detail), and (5) abnormal umbilical artery Doppler defined as pulsatility index in the last research scan (36 wkGA scan but 28 wkGA scan if 36 wkGA scan result was missing (see Sovio et al.^13^ for details).

### Transcriptome reconstruction

In addition to the four samples mentioned above, three samples classified as both PE and FGR were also excluded in transcriptome reconstruction process. Thus, a total of 295 unique placental biopsies were used (Supplementary Fig. 1). Nineteen control samples were sequenced twice (i.e., replicates) in two different libraries and only one replicate was chosen based on the following criteria: replicates sequenced recently if the biopsies are from the planned caesarean section (three samples); otherwise replicates sequenced together with FGR cases (16 samples). For each placental sample, we reconstructed a transcriptome assembly using *StringTie* (v.1.2.3)^22^ guided by the reference transcript annotation from Ensembl v82^89^. To qualify as a valid reconstructed transcript we applied a threshold of 10 and 5 reads as a minimum base-pair coverage (*-c*) and junction coverage (*-j*), respectively. Then, we assembled a placental transcriptome from 295 reconstructed transcript files (in GTF formats) using *cuffcompare* tool of *cufflinks* software package (v.2.2.1)^24^ aided by the same version of the reference human transcript annotation (Ensembl 82) used at the alignment (*TopHat2*) and assembly (*StringTie*) steps. We used three additional meta-assemblers, *gffcompare*, TACO^23^ and StringTie (with merge option), and assessed the degree of agreement with the reference transcript annotation (Ensembl v82) using g*ffcompare* utility (v.0.10.6; https://github.com/gpertea/gffcompare).

### Novel reconstructed placental transcripts

We selected reconstructed transcripts for further analysis based on the following two conditions: (1) present in at least 10% of the total number of samples (i.e., supported by at least 30 samples out of 295 total samples) and (2) expressed at least 0.1 RPKM. These reconstructed transcripts were annotated as per the ‘transfrag class codes’ of *cuffcompare* tool of *cufflinks* software package (v.2.2.1)^24^, from which we considered the following five codes as putative novel isoforms: (1) potentially novel isoform (code: j), (2) within a reference intron (code: i), (3) unknown, intergenic transcript (code: u), (4) exon on the opposite strand (code: x), and (5) generic exonic overlap (code: o). To identify novel transcripts, we compared our reconstructed transcripts with the CHESS database (v2.1)^25^ using *gffcompare* (https://github.com/gpertea/gffcompare).

### Target mRNAs of highly expressed miRNAs in C19MC

Thirty-three mature miRNAs present in C19MC were within the most abundant 5% mature miRNAs. Their binding target mRNAs were selected from miRTarbase (v8.0)^19^ if they satisfied the following two conditions: (1) functional mRNA-target interactions only, and (2) interactions supported by at least three pieces of strong evidence. There were 34 miRNA-target pairs satisfying aforementioned conditions, consisting of 14 and 27 unique miRNAs and mRNAs, respectively. The correlation between the expression level of 14 miRNAs and their 27 target mRNAs were calculated using ‘*cor.test*’ of R package. Gene Ontology (GO) analysis of the 27 binding targets was conducted using g:Profiler^90^ (version e101_eg48_p14_baf17f0).

### Analysis of miRNA-mRNA interactions

We followed the approach of Liu et al.^38^, using WGCNA^37^ to identify miRNA-mRNA co-expression networks. For the 288 samples for which both miRNA and mRNA data were available (88 PE, 52 FGR, and 148 control samples), we concatenated the log_2_(*x* + 1)-transformed RPKM miRNA and mRNA data matrices, and used the ‘*WGCNA*’ R package to identify 22 network modules of highly correlated transcripts, plus an additional “null” module (module 0) comprising transcripts that could not be allocated to any other module. We performed logistic regression analyses to test for association between each module eigengene and patient group (PE, FGR, control), using the ‘*qvalue*’ Bioconductor package to control the local false discovery rate at the 0.05 level. We used Fisher’s exact test to identify modules that contained a greater number of miRNAs and known miRNA targets than expected by chance, using Bonferroni correction to address the multiple comparisons problem. GO, Reactome, and WikiPathways term enrichment analyses were performed using the R interface to g:Profiler, and adopting the g:SCS correction to control for multiple testing while taking into account the directed acyclic graph structure of the Gene Ontology.

### Identification of circRNAs and their possible miRNA targets

Initially circRNAs were identified in non-oligo-dT primed or selected RNA-Seq datasets (i.e., rRNA depleted a “ribo-minus” library; Supplementary Data 8). We then identified circRNAs in an oligo-dT primed placental RNA-Seq dataset (i.e., poly-A^+^ library) from the same cohort (*n* = 60, Supplementary Data 9). Such RNAs could be artefactual so we removed all these circRNAs from the list of those found in the rRNA-depleted libraries. Adaptor-trimmed and quality-assured reads of both RNA-Seq datasets were mapped to GRCh38 version of human genome reference using *BWA-MEM* algorithm of *BWA* (v0.7.17-r1188)^91^ with a minimum score to output (*-T*) 19. Lane-wise aligned SAM files were combined for each sample and the merged SAM files were analyzed with CIRI2 (v2.0.6)^92^. This is a software tool for detecting circRNAs that can differentiate back-splice junction (BSJ) reads from non-BSJ reads, with its default parameters. We used Ensembl v90 transcript annotation file to annotate types of genomic positions (e.g., exons, introns, or inter-genic regions) where the back-spliced junctions are located. We applied a threshold of minimum sample frequency 30% (i.e., POPS30) to the circRNAs predicted to be present in the rRNA-depleted dataset, then filtered and removed them (1) if the back-spliced products spanned multiple genes (e.g., read-through) and (2) further subtracted those present in at least one sample from the poly-A^+^ library dataset. There were 3,304 circRNAs in the POPS30 rRNA-depleted dataset and 217 in the poly-A^+^ dataset—25 were identified in both datasets and they were excluded from the analysis. As there is no official nomenclature of circRNAs, we used the following pieces of information to identify a circRNA: chromosome, start position, and end position (e.g., chrX:140,783,175-140,784,659) where the start and end position are the splice-acceptor and splice-donor positions, respectively, of BSJ sites predicted by CIRI2. The chromosome coordinates and the number of BSJ of 3452 and 217 circRNAs are shown in Supplementary Data 8 and 9, respectively. We generated FASTA files of 3399 circRNAs in POPS30 using *Biosrings* and *BSgenome.Hsapiens.UCSC.hg38* packages of Bioconductor based on the aforementioned coordinates of circRNA and we predicted their putative miRNA-binding sites using miRanda (v3.3a)^31^ with a ‘*-strict*’ option which demands a strict 5′ seed pairing. We analyzed the sequence motif of miRNA-binding regions using MEME suite of programs^93^.

### Identification of translated circRNAs

To identify peptides derived from translated circRNAs, we searched two publicly available large proteome-wide datasets. Namely “the draft map of the human proteome” dataset^34^, containing deep proteomic profiling of 17 adult tissues, 7 fetal tissues, and 6 purified primary hematopoietic cells and the “proteome and transcriptome abundance atlas”^36^ containing profiles from 29 healthy human tissues. Raw files were obtained from the PRoteomics IDEntifications (PRIDE) database^94^ (projects PXD000561 and PXD010154) and converted to Mascot generic format (MGF) using the ThermoRawFileParser package (version 1.1.8, https://github.com/compomics/ThermoRawFileParser).

Two approaches were taken to identify peptides from translated circRNAs: (i) searching for peptides that span the back-splice junction and (ii) searching for peptides from single-exon circRNAs.

The first approach is based on the three reading-frame translations of RNA sequences spanning the back-splice junction. Therefore, 99 nucleotides upstream and downstream of the back-splice junction are translated to a protein sequence in three reading frames. In the second approach only circRNAs that arise from the circularization of a single exons are considered. The RNA sequence of the entire exon was obtained and translated to a protein sequence in three reading frames.

Peptide databases are created as a full in silico trypsin digestion (allowing up to one missed cleavage) of the protein sequence dataset consisting of all human protein isoforms in the UniProtKB database^95^ (UP000005640 proteome, 73,101 protein isoforms), protein sequences in the common Repository of Adventitious Proteins (cRAP) database (https://www.thegpm.org/crap) and the in silico translated circRNA sequences. For false discovery rate (FDR) estimation, a decoy peptide database is constructed by reversing the protein sequences in the target database followed by full in silico trypsin digestion with one missed cleavage.

Analysis of the tandem mass spectrometry data has been performed using Ionbot (ionbot (manuscript in preparation; http://compomics.com/ionbot, based on the work of Silva et al.^96^), a sequence database search tool based on machine learning capable of performing rapid open modification and open mutation searches. Here, Ionbot was run without the open modification and mutation functionality. Ionbot was used under a beta-tester version supplied by Sven Degroeve and Lennart Martens (Ghent University, VIB). As part of the Ionbot pipeline, Percolator^97^ has used to re-score the peptide-spectrum matches. Next, the peptide-spectrum matches (PSMs) are analyzed according to the “search all, analyze subset” strategy^98^, ensuring correct estimating of the FDR for the peptides mapping to circRNAs. Thus, only PSMs mapping uniquely to circRNAs (or circRNA junctions in approach I) and the corresponding decoy sequences are considered. The FDR was estimated with the target-decoy approach and calculated as:

$${\mathrm{FDR}}=\frac{\#{\mathrm{decoy}}}{\#{\mathrm{target}}}$$

Positive hits are filtered at the score calculated by percolator corresponding to the 1% FDR threshold.

After FDR filtering, 55 PSMs corresponding to 5 peptides mapping to circRNA BSJs and 93 PSMs corresponding to 15 unique peptides derived from single-exon circRNAs remained. Next, the peptide retention time was used as a mean of orthogonal validation of the PSMs. Therefore, the observed retention time was compared to the retention time predicted by DeepLC^99^ (version 0.1.17). Thousand high-scoring Uniprot peptides were used for calibration. A 95% confidence interval on the predicted retention time was calculated from peptides mapping to Uniprot. While all five peptides mapping to circRNA BSJs had a predicted retention time within this 95% confidence interval, of the 15 single-exon circRNA peptides, only six had retention times within the interval, highly indicative of a misidentification. Upon manual inspection, two out of five peptides mapping to BSJ sequences and three out of the six peptides mapping to single-exon circRNAs could be derived from isoforms of Uniprot proteins absent in the search database. Of the three remaining peptides mapping to the BSJ of circRNAs (GTGLDEAMEWWLFLK, TSATKGTGLDEAMEWWLFLK, NALKQIWDTAGQER), two maps to the same circRNA. For the single-exon circRNAs, three peptides with a very low number of PSMs (<5 PSMs across the entire collection) remain IIELTALR, LLLPQSVSLIVMR, and GDQKQWEETTR. While it is possible that these are indeed circRNA derived peptides, this low number of identifications falls well within the 1% FDR range and should thus be interpreted with extreme caution.

### RT-qPCR and Sanger sequencing

Reverse transcriptase (RT) reactions were performed on seven samples of human placental RNA isolated as described above (500 ng/sample), using the “Super Script IV VILO Master Mix” (ThermoFisher Scientific) following the manufacturer’s instructions. One reaction lacking the reverse transcriptase enzyme was included to identify any possible amplification of genomic DNA. Quantitative PCR (qPCR) analysis was performed on diluted cDNAs (1:10) using the “Power Up Sybr Green Master Mix” and the QuantStudio 6 instrument (both from ThermoFisher Scientific). The divergent primers employed in the qPCR assays are described in Supplementary Data 21. In order to check the amplified product size, the qPCR products (one well of each triplicate) were run on a 1% agarose gel together with a 1 kb ladder. The other two wells of each qPCR assay were combined, purified using the “QIAquick PCR Purification Kit” (Qiagen) and Sanger sequenced at the DNA Sequencing Facility of the Department of Biochemistry (University of Cambridge) using both the forward and reverse primers described above.

### Transcript quantitation and differential expression analysis

For total RNA-Seq datasets, we measured transcript abundance using two software tools: *featureCounts* (v1.5.1)^100^ and *Salmon* (v.0.9.1) in quasi-mapping-mode^101^. The quantifications were made against two sets of transcriptome definitions: (i) reference-based transcript annotation (Ensembl v82) and (ii) reconstructed novel placental transcripts present in at least 10% of 295 samples used in the placenta transcriptome reconstruction. In the case of *Salmon*, the transcript level abundance was merged at the gene level. For the qualification of miRNA and piRNA see ‘RNA-Seq data processing’ section above. We identified differentially expressed genes (shown in Table 1) using a *P* value-based approach and a fold change approach. For both approaches, we used transcripts passing the following filters: (1) RPM ≥ 0.2 for non-circRNA; count ≥5 for circRNA, (2) present in >10% of the cohort, (3) found in both the FGR and PE cohorts, and 4) genes not on chrY and chrMT. For both the PE and FGR cohorts, we used a multi-factor design to take into account the case-control pair information (e.g., ~pair + condition) when performing *DESeq2* (v.1.18.1). The original *P* values were obtained from *DESeq2* and they were further adjusted for multiple comparisons using the Benjamini-Hochberg correction method implemented by *p.adjust* function of R *stats* package. Note that for circRNAs, the number of back-spliced junction reads was used as the input for *DESeq2*.

### Bootstrapping samples for differential expression analysis

We sampled the cases and controls to generate the same number of pairs as in the initial data, *n* = 82 or 40 pairs for PE and FGR respectively. We repeated this 10,000 times with replacement using the *sample* function of R base package. We calculated the mean RPM of the resampled cases and controls. Then we took the ratio of RPM between cases and controls for each qualified transcript that had passed the aforementioned filters. Finally, we calculated the bootstrap-generated fold-changes by taking the mean over 10,000 times. This can be summarized in the following series of equations:

1 $${{\rm{FC}}}_{i}^{k}:{\mathrm{Fold-change}}\,{\mathrm{of}}\,{\mathrm{gene}}\,i\,{\mathrm{at}}\,{\mathrm{the}}\,k-{\mathrm{th}}\,{\mathrm{bootstrapping}},\,{\mathrm{where}}\,k\,{\mathrm{goes}}\,{\mathrm{from}}\,1\,{\mathrm{to}}\,10,000$$

2 $${{\rm{FC}}}_{i}^{k}={\mathrm{mean}}\,{{\rm{RPM}}}_{i}^{k}({\rm{bootstrapped}}{\hbox{-}}{\rm{cases}})/{\rm{mean}}\,{{\rm{RPM}}}_{i}^{k}({\rm{bootstrapped}}{\hbox{-}}{\rm{controls}})$$

3 $${\mathrm{FC}}i=(\mathop{\sum }\limits_{k=1}^{n}{{\rm{FC}}}_{i}^{k})/n$$

4 $${\mathrm{log}}2(F{C}_{i})={\mathrm{log}}2(\frac{{\sum }_{k}^{n}{{\rm{FC}}}_{i}^{k}}{n})$$

The magnitude of the fold change can be misleading when the transcript is very poorly expressed. We therefore used the most abundant transcripts, selecting the 5000 most abundant protein-coding transcripts from 15,257 transcripts passing the filters described above. We used the same selection ratio (i.e., 5000/15257) for the other types of transcript. For all qualified transcripts, the bootstrap-generated fold changes (i.e., $2(FCi)$) were ranked in descending order and the top 5% and top 3% transcripts were selected from the most abundant transcripts. There were 250 transcripts within the top 5% highly ranked protein-coding transcripts (150 in top 3%), where 95 of them were shared between PE and FGR (92 were in same direction of fold change; 3 were in opposite direction). We performed the Fisher’s exact test to calculate the significance of the number of top-ranked transcripts shared by the PE and FGR cohorts. We only considered top-ranked transcripts which changed in the same direction shared in both conditions using most abundant transcripts as background number. For example, the significance level of observing 92 transcripts shared in the PE and FGR samples, given the selection of 250 transcripts (i.e., top 5%) out of 5000 most abundant transcripts for each cohort, was performed from the following matrix using *fisher.test* of R package: $$\left({{92}\atop{250-92}} \\ \quad{{250-92}\atop{5214-250-250+92}} \right)$$. Note, there were 5214 unique transcripts from the union of 5000 transcripts from each of the PE and FGR samples. The same selection ratio (i.e., 5000/15257) was applied to other types of transcripts.

### Identification of transcripts enriched in the placenta

To identify transcripts that are expressed specifically in the placenta, we compared our data with the 49 somatic tissues from GTEx (v8.p2)^1^. To select eligible samples from the GTEx RNA-Seq datasets, we used a set of filtering conditions similar to our previous study^15^ with minor modifications: (a) RNA integrity number (SMRIN) ≥6, (b) mapping rate (SMMAPRT) ≥0.9, (c) exonic mapping rate (SMEXNCRT) ≥0.75, and (d) ≥20 qualifying samples per tissue. Five tissues (out of 54) were dropped after applying aforementioned quality control measures: the kidney (Kidney - Medulla), the fallopian tube, the cervix endocervix, the cervix ectocervix, and the bladder. Finally, a total of 13,070 samples were selected from the 49 tissues (including two cell lines: cultured fibroblasts and EBV-transformed lymphocytes). We considered 56,156 genes from the gene-level quantification information available from the following file: GTEx_Analysis_2017-06-05_v8_RNASeQCv1.1.9_gene_reads.gct.gz (https://storage.googleapis.com/gtex_analysis_v8/rna_seq_data/GTEx_Analysis_2017-06-05_v8_RNASeQCv1.1.9_gene_reads.gct.gz). We generated a count matrix of 56,156 genes by 13,270 samples (i.e., 13,070 samples from GTEx and 200 from the placenta (171 from Ribo-Zero and 29 from the oligo-dT library), then filtered out 259 genes of the following conditions: (1) the sum of read count across all samples is zero (*n* = 169), and (2) non-polyadenylated RNAs (e.g., transcripts of major histones) reported from the study of Yang et al. (*n* = 90). After this filtering, a total of 55,897 genes were considered in this study. To adjust differences in the composition of RNA populations across multiple tissues, we applied the trimmed mean of *M*-values (TMM)^102^ (available from ‘calcNormFactors’ function of edgeR Bioconductor package^103^) to the count matrix. We then built a matrix of RPKM per tissue using the ‘rpkmByGroup’ function of edgeR package, which reduced the columns (i.e., 13,270 samples) of the matrix to a size of 52 columns (i.e., 49 tissues from GTEx and 2 placenta datasets (Ribo Zero and oligo-dT). Transcript abundance measured in RPKM was converted to TPM using the formula of Pachter^104^. For each gene, we calculated the *Tau* score (a tissue-specificity score^105^), using TPM values across 50 tissues (49 tissues from GTEx and the placenta Ribo-Zero dataset) and flagged genes as “tissue enriched” if they satisfy the following conditions: (i) *Tau* score^105,106^ >0.99, (ii) TPM (query tissue) >1, (iii) TPM (query tissue) >mean TPM (remaining tissues) * 100, and (iv) ‘protein coding’ or ‘lincRNA’ as per the gene biotype in Gencode v26 (Ensembl v88). The same conditions were applied using the placental oligo-dT dataset and the list of placenta enriched transcripts was compared with those discovered from the placenta Ribo-Zero dataset. The aforementioned conditions were modified as follows, to investigate transcripts encoded by endogenous retroviruses in the placenta (Supplementary Data 7): (i) Tau score > 0.9, (ii) TPM in placenta (Ribo-Zero) is >10 times of the average TPM of the 49 non-placental tissues. As GTEx RNA-Seq datasets are based on polyA^+^ selected RNA, we manually determined whether the candidate transcripts were polyadenylated or not using datasets from Yang et al.^17^

### Size distributions in small RNA-Seq datasets

To count ‘Mapped reads’ in Supplementary Fig. 6, quality-assured trimmed small RNA-Seq reads were mapped to the GRCh38 version of human genome reference sequence using *mapper.pl* script of *miRDeep2* (v2.0.0.7)^82^, and the mapped reads were parsed by *parse_mappings.pl* script with the following parameters: *-a 0 -i 3*. Mapped reads of at least 10× coverage were considered:

*parse_mappings.pl $ARF_FILE -a 0 -i 3 \*

|*awk ‘BEGIN{OFS=“,”}{split($1,seq,”_x”); if(seq[2]>=10) print $1,$2,seq[2]}’ \*

|*sort* | *uniq*

Then, reads from the ‘Mapped reads’ were removed if they overlap with any mature miRNA regions annotated from *mirBase* (v21)—their read counts are shown as ‘-miRNAs’:

*parse_mappings.pl $ARF_FILE -a 0 -i 3 \*

|*awk ‘BEGIN{OFS=“\t”}{if($6==“MT”){$6=“M”}; start=$8-1; split($1,seq,”_x”); if(seq[2]>=10) print “chr”$6,start,$9,$1,seq[2],$11}’ \*

|*bedtools intersect -a stdin -b $MIRBASE_V21.bed -s -v \*

|*awk ‘BEGIN{OFS=“,”}{width=$3-$2; print $4,width,$5}’ | sort*|*uniq*

From the ‘-miRNAs’, reads were further removed if they overlap with any from the following sources: (1) known precursor miRNAs annotated by *mirBase*, (2) novel miRNAs predicted by *miRDeep2,* (3) *piRNAs* annotated from *piRBase*, and (4) exonic regions defined by Ensembl gene model. These remaining reads mapped to ‘unannotated’ genomic regions are shown as ‘-miRNA-piRNA-exon’ and they are used as the source for ‘novel small-RNA’:

*parse_mappings.pl $ARF_FILE -a 0 -i 3 \*

|*awk ‘BEGIN{OFS=“\t”}{if($6==“MT”){$6=“M”}; start=$8-1; split($1,seq,”_x”); if(seq[2]>=10) print “chr”$6,start,$9,$1,seq[2],$11}’ \*

|*bedtools intersect -a stdin -b $MIRBASE_V21.bed $NOVEL_MIRNA.bed $PIRBASE_V1.bed $ENS_EXON.bed -s -v \*

|*awk ‘BEGIN{OFS=“,”}{width=$3-$2; print $4,width,$5}’ | sort* | *uniq*

### Maternal serum immunoassays

Circulating FSTL3 was measured in maternal serum samples collected at 36 wkGA during the POP study. We selected 94 PE cases, 86 FGR cases, and a random sub-cohort of 334 women (comparison group). From the random sub-cohort, (i) we excluded 10 women who had a small for gestational age (SGA) infant but no indicators of FGR, and (ii) re-labeled women who fulfilled the case status for each analysis (21 cases of PE only, 13 cases of FGR only and 1 case of both PE and FGR, respectively). In total, 495 samples were analyzed, including 289 non-cases and 206 cases (106 with PE only, 90 with FGR only, and 10 with both PE and FGR; therefore 116 PE cases and 100 FGR cases). FSTL3 was measured using the Human FSTL3 (FLRG) Quantikine ELISA Kit (R&D Systems, cat # DFLRG0). Multiples of the median (MoM) of the average concentrations of FSTL3 were calculated, referent to the random sub-cohort of controls. The MoMs were then corrected for gestational age (GA, weeks) and maternal weight (WT, kg) at the time of measurement and for sample storage time (SST, years) at the time of sample processing applying a log-linear regression procedure to the random sub-cohort. This resulted in the following equation for the corrected MoMs, which was applied to all samples: corrected MoM = uncorrected MoM / 10^(–2.261734+0.0657065*GA–0.0011209*WT–0.001105*SST)^. A 97th percentile cut-off of the corrected MoMs was calculated in the random sub-cohort, and the corrected MoMs in all women were dichotomized according to this cut-off. The outcomes were assessed in all deliveries subsequent to the 36-week measurement. PE was defined according to the ACOG2013 classification^71^. FGR was defined as delivery of an infant with birth weight <3rd percentile (using fetal sex and gestational age-adjusted reference standard derived from a UK population^107^) or birth weight <10th percentile plus at least one of the following: (a) top decile of uterine artery Doppler mean Pulsatility Index (PI) at 20 wkGA, (b) top decile of umbilical artery Doppler PI at 36 wkGA, (c) lowest decile abdominal circumference growth velocity from 20 to 36 wkGA, (d) neonatal morbidity, or (e) maternal PE^13^.

### Correlations between placental mRNAs and serum metabolites

We selected transcripts as for the other analyses (RPM ≥ 0.2 and present in >10% of the cohort). We used the circulating metabolite data from the samples collected at 36 wkGA reported by Sovio et al.^39^ (metabolite identifiers are provided in Supplementary Data 24). We removed the data for the metabolites that had a standard deviation of zero (the drugs—carbamazepine and its metabolites and cimetidine). Transcript and metabolite data were available from 89 subjects. We calculated the Spearman correlation between the four metabolites associated with FGR and the 24,611 transcripts using *cor* (stats package in R 3.6.3). We filtered this data to remove all metabolites and transcripts for which the values of Spearman’s *rho* were all >−0.4 and <0.4. Adjusted (Benjamini-Hochberg) *P* values were calculated using *correlation* (correlation package v 0.4.0). We performed a similar analysis using all 1,185 metabolites; in this instance we filtered this data to remove all metabolites and transcripts for which the values of Spearman’s *rho* were all >−0.5 and <0.5. Heatmaps were generated using *pheatmap* (v1.0.12). Transcripts for which the correlation with the individual metabolites had a *P*_adj_ ≤ 0.05 were analyzed for the over representation of GO terms (Panther v15.0^108^).

## Supplementary information

Supplementary Information

Supplementary Data 1-27

Supplementary Data 28-74

Description of Additional Supplementary Files

## Data Availability

All the computational analyses were conducted using the Linux clusters at the University of Cambridge High Performance Computing Service and the Linux workstations of School of Biological Science computing. The RNA-Seq datasets generated during the current study are available in the European genome-phenome archive (EGA, https://www.ebi.ac.uk/ega) with the following accession codes: EGAD00001003457, EGAD00001003507, EGAD00001003508, EGAD00001006304, and EGAD00001004860. The curated datasets described in the current study can be found at https://www.obgyn.cam.ac.uk/placentome (ref. ^109^). The datasets used in this study are available from miRbase v21 (https://mirbase.org/pub/mirbase/21), piRBase v1, miRTarBase v8, CHESS database v2.1 and GTEx v8.p2, PRIDE database with the following two accession codes: PXD000561 and PXD010154, the common Repository of Adventitious Proteins (cRAP) database (https://www.thegpm.org/crap) and UniProtKB database with the following accession code: UP000005640. The data supporting the findings of this study are available from the corresponding authors upon reasonable request. Source data for the figures are provided as a Source Data file.

## Code availability

Code used in this study is available in the Methods section and at https://github.com/sung/POPS-Placenta-Transcriptome-2020. Code used to build the website is available at https://github.com/sung/ShinyPlacentome (ref. ^109^).
