# Supplementary material for: The RNA landscape of the human placenta in health and disease
Source: Nat Commun. 2021 May 11;12:2639. doi: 10.1038/s41467-021-22695-y (PMC8113443; doi:10.1038/s41467-021-22695-y)
Supplement: Supplementary file 1 — Supplementary Information [file 41467_2021_22695_MOESM1_ESM.pdf]

SUPPLEMENTARY INFORMATION

The RNA landscape of the human placenta in health and disease

Sungsam Gong, Francesca Gaccioli, Justyna Dopierala, Ulla Sovio, Emma Cook, Pieter-Jan Volders, Lennart Martens, Paul DW Kirk, Sylvia Richardson, Gordon CS Smith and D Stephen Charnock-Jones

Supplemental Note

Leptin transcript variants

We identified 12 re-constructed *LEP* transcript variants, three of which were present in at least 30 placental RNA-seq samples (i.e. 10% of cohort) with a minimum expression level of RPKM>0.1. One of these three corresponds to the reference transcript model (ENST00000308868) and the remaining two are annotated as 'potentially novel isoforms' where their exon boundaries differ from the reference transcript (Supplementary Figure 7a and Supplementary Data 18). The reference-matching transcript (TCONS_00323031) is the most abundant (RPKM=6.6) and the most frequently detected (present in 268 samples (91%) of 295 samples used in the transcript re-construction). TCONS_00329506 (RPKM=2.6) is present in 41 samples) and TCONS_00320483 (RPKM=1.8) present in 31 samples. TCONS_00320483, however, consists of two exons, as opposed to three exons in the reference transcript, where the first intron of the reference transcript is retained in the first exon of this two-exon transcript variant. This is likely due to transcriptional or splicing background noise[1].

The transcription start sites (TSS) of the two novel isoforms are extended by 71bp (TCONS_00329506) and 38bp (TCONS_00320483) toward 5' upstream relative to the TSS of the reference transcript (ENST00000308868). The transcript end sites (TES) of the two novel isoforms are extended by 1bp (TCONS_00329506) and 41bp (TCONS_00320483) toward 3' downstream relative to the TES of the reference transcript (Supplementary Data 18). The RNA-Seq read coverage, however, diminishes rapidly as it extends both from the TSS and TES of the reference transcript.

The novel isoforms (TCONS_00329506 and TCONS_00320483) also differ in the exon boundaries which change the coding sequence. The start positions of the last exon are three bases less than the reference transcript. This removes a codon (CAG) encoding a glutamine (Q) at the 49th amino acid when translated (Supplementary Figure 7b). The missing glutamine has been previously annotated in UniProt (http://www.uniprot.org/uniprot/P41159#Natural_variant_section)[2]. This was first reported by Zhang *et al* in 1994[3] who found 30% of the clones analyzed were missing the glutamine at codon 49. This is possibly due to slippage at the splice-acceptor site as the codon (CAG) itself also contains an AG splice-acceptor sequence (Supplementary Figure 7b). The transcript with the missing glutamine codon was also predicted as one of *LEP* transcript variants in RefSeq (https://www.ncbi.nlm.nih.gov/nuccore/XM_005250340). The CHESS database[1] also supports this rare *LEP* transcript with the missing glutamine codon. The glutamine residue is located in a highly conserved region of leptin and it is retained across the mammals but absent in some birds (e.g. chicken and turkey; Supplementary Figure 7c). We determined whether

this rare *LEP* transcript is also present in other human tissues. It is present, at low frequency in other placenta-related tissues (e.g. amnion, basal plate, villi, trophoblast and chorion) and some of somatic tissues such as adipose tissue and the bladder (Supplementary Data 19).

Even though the reference-matching transcript (TCONS_00323031) was the most abundant form, the two rarer *LEP* transcript variants (TCONS_00329506 and TCONS_00320483) are more frequently observed in the samples of pre-eclampsia than expected (Supplementary Figure 7d). For example, TCONS_00329506 is present in 41 samples and it is 1.4 times more frequent in samples of pre-eclampsia (56%) than healthy controls (39%, P=0.0011 chi-squared test).

Assessment of reconstructed placenta transcriptome

To evaluate our pipeline used in placenta transcriptome, we used three additional meta-assemblers, Gffcompare (https://github.com/gpertea/gffcompare), TACO[4] and StringTie (with merge option; StringTie-merge hereafter), and assessed the degree of agreement with the reference transcript annotation (Ensembl v82). The performance of Cuffcompare[5] (applied in this study) is very similar to that of Gffcompare and TACO outperformed StringTie-merge, as described in their original study[4], and it exceeded other methods in terms of precision (Supplementary Figure 5e). However, when we applied a minimum level of sample frequency, for example, ≥10% threshold, the precision became twice as high as that of the original cuffcompare and it exceeded that of TACO. This was achieved by sacrificing some degree of sensitivity, but even this trade-off seemed worthwhile as it still outperformed TACO.

Analysis of miRNA-mRNA interactions

To investigate the relationships between miRNA-mRNA pairs, we followed the approach of Liu *et al.*[6] using weighted gene co-expression network analysis (WGCNA)[7]. For the 288 samples for which both data types were available (88 PE, 52 FGR and 148 control samples), we concatenated the miRNA and mRNA data matrices, and used the *WGCNA* R package to identify 22 network modules of highly correlated transcripts, plus an additional “null” module (module 0) comprising transcripts that could not be allocated to any other module (for module membership details, see Supplementary Information File 3). Using eigengene network methodology, we found that most modules were at least weakly related to patient group (PE, FGR; for full results of tests for association between module eigengenes and patient groups see Supplementary Information File 3). We identified modules 10 and 11 as being significantly enriched for miRNAs (Fisher exact test, $P < 0.05$; see Supplementary Information File 3). Of the 12 miRNAs differentially expressed between PE and controls (see Table 1), 2 were in module 10 (miR-126-3p and miR-221-3p), 6 were in module 11 (miR-7641, miR-371a-3p, miR-372-3p, miR-373-3p, miR-10b-5p, and miR-659-5p), and the remaining 4 were allocated to module 0. Using the R interface to *g:Profiler*[8], we performed enrichment analysis for modules 10 and 11 (see Figure 4a and 4b). Full results of the *g:Profiler* enrichment analysis for these and all other modules are provided in Supplementary Information File 3. These tables contain overrepresented gene ontology terns (biological process, molecular function and cellular compartment, BP, MF and CC respectively); as well as overrepresented KEGG, Reactome (REAC), WikiPathways(WP), Human Protein Atlas (HPA), TRANSFAC (TF), miRTarBase (MIRNA), CORUM database (CORUM), and Human phenotype ontology (HP)

annotations. Multiple testing correction was performed using the *Set Count and Sizes* (SCS) algorithm described in Reimand *et al*.[9], with threshold *P* = 0.05. An R script to reproduce all of the analyses described above is available at: https://github.com/sung/POPS-Placenta-Transcriptome-2020

Putative binding sites of circRNA CDR1as (chrX:140,783,175-140,784,659) predicted by miRanda

```
Read Sequence:hsa-miR-7-5p (23 nt)
Read Sequence:ENST00000625883.1 AL078639.1-201 cdna:antisense
(1485 nt)
=-=-=-=-=-=-=-=-=-=-=-=-=-=-=-=-=-=-=-=-=-=-=-=-=-=-=-=-=-=-=-=-=
Performing Scan: hsa-miR-7-5p vs ENST00000625883.1
=-=-=-=-=-=-=-=-=-=-=-=-=-=-=-=-=-=-=-=-=-=-=-=-=-=-=-=-=-=-=-=-=

   Forward: Score: 174.000000  Q:2 to 20  R:207 to 230 Align Len (19)
(78.95%) (94.74%)

   Query:    3' uguuGUU-UUAGUGAUCAGAAGGu 5'
                    ||: |||:|||:|||||||
   Ref:      5' cttcCAGCAATTACTGGTCTTCCa 3'

   Energy:  -28.270000 kCal/Mol

Scores for this hit:
>hsa-miR-7-5p     ENST00000625883.1 174.00      -28.27      2 20   207 230
      19    78.95%      94.74%


   Forward: Score: 170.000000  Q:2 to 21  R:356 to 377 Align Len (19)
(84.21%) (84.21%)

   Query:    3' uguUGUUUUAGUGAUCAGAAGGu 5'
                   || ||||| | ||||||||
   Ref:      5' tccACCAAATC-CAAGTCTTCCa 3'

   Energy:  -20.150000 kCal/Mol

Scores for this hit:
>hsa-miR-7-5p     ENST00000625883.1 170.00      -20.15      2 21   356 377
      19    84.21%      84.21%


   Forward: Score: 170.000000  Q:2 to 21  R:818 to 839 Align Len (19)
(84.21%) (84.21%)

   Query:    3' uguUGUUUUAGUGAUCAGAAGGu 5'
                   | |||||| || |||||||
   Ref:      5' tccAGAAAATC-CTTGTCTTCCc 3'

   Energy:  -18.879999 kCal/Mol

Scores for this hit:
>hsa-miR-7-5p     ENST00000625883.1 170.00      -18.88      2 21   818 839
      19    84.21%      84.21%
```

```
   Forward: Score: 170.000000  Q:2 to 21  R:944 to 965 Align Len (19)
(84.21%) (84.21%)

   Query:    3' uguUGUUUUAGUGAUCAGAAGGu 5'
                   | |||||| || |||||||
   Ref:      5' tccAGAAAATC-CTTGTCTTCCc 3'

   Energy:  -18.879999 kCal/Mol

Scores for this hit:
>hsa-miR-7-5p     ENST00000625883.1 170.00      -18.88      2 21  944 965
      19    84.21%      84.21%


   Forward: Score: 167.000000  Q:2 to 22  R:432 to 453 Align Len (20)
(75.00%) (85.00%)

   Query:    3' ugUUGUUUUAGUGAUCAGAAGGu 5'
                  || :||||| | :|||||||
   Ref:      5' agAAAGAAATC-CAGGTCTTCCa 3'

   Energy:  -18.930000 kCal/Mol

Scores for this hit:
>hsa-miR-7-5p     ENST00000625883.1 167.00      -18.93      2 22  432 453
      20    75.00%      85.00%


   Forward: Score: 167.000000  Q:2 to 22  R:471 to 492 Align Len (20)
(75.00%) (85.00%)

   Query:    3' ugUUGUUUUAGUGAUCAGAAGGu 5'
                  || :||||| | :|||||||
   Ref:      5' agAAAGAAATC-CAGGTCTTCCa 3'

   Energy:  -18.930000 kCal/Mol

Scores for this hit:
>hsa-miR-7-5p     ENST00000625883.1 167.00      -18.93      2 22  471 492
      20    75.00%      85.00%


   Forward: Score: 167.000000  Q:2 to 22  R:564 to 585 Align Len (20)
(75.00%) (85.00%)

   Query:    3' ugUUGUUUUAGUGAUCAGAAGGu 5'
                  :| |||||| | :|||||||
   Ref:      5' cgGAAAAAATC-CAGGTCTTCCa 3'

   Energy:  -19.400000 kCal/Mol

Scores for this hit:
>hsa-miR-7-5p     ENST00000625883.1 167.00      -19.40      2 22  564 585
      20    75.00%      85.00%


   Forward: Score: 166.000000  Q:2 to 21  R:526 to 547 Align Len (19)
(78.95%) (84.21%)

   Query:    3' uguUGUUUUAGUGAUCAGAAGGu 5'
                   || ||||| | :|||||||
```

```
   Ref:      5' tccACCAAATC-CAGGTCTTCCa 3'

   Energy:  -19.180000 kCal/Mol

Scores for this hit:
>hsa-miR-7-5p     ENST00000625883.1 166.00      -19.18      2 21  526 547
      19    78.95%      84.21%


   Forward: Score: 166.000000  Q:2 to 21  R:854 to 875 Align Len (19)
(78.95%) (84.21%)

   Query:    3' uguUGUUUUAGUGAUCAGAAGGu 5'
                   | |||||| | :|||||||
   Ref:      5' tccAAAAAATC-CGGGTCTTCCa 3'

   Energy:  -20.940001 kCal/Mol

Scores for this hit:
>hsa-miR-7-5p     ENST00000625883.1 166.00      -20.94      2 21  854 875
      19    78.95%      84.21%


   Forward: Score: 166.000000  Q:2 to 22  R:909 to 929 Align Len (20)
(75.00%) (85.00%)

   Query:    3' ugUUGUUUUAGUGAUCAGAAGGu 5'
                  ||||||: ||:  |||||||
   Ref:      5' ccAACAAAGCCAT--GTCTTCCa 3'

   Energy:  -23.920000 kCal/Mol

Scores for this hit:
>hsa-miR-7-5p     ENST00000625883.1 166.00      -23.92      2 22  909 929
      20    75.00%      85.00%


   Forward: Score: 166.000000  Q:2 to 22  R:1035 to 1055 Align Len (20)
(75.00%) (85.00%)

   Query:    3' ugUUGUUUUAGUGAUCAGAAGGu 5'
                  ||||||: ||:  |||||||
   Ref:      5' ccAACAAAGCCAT--GTCTTCCa 3'

   Energy:  -23.920000 kCal/Mol

Scores for this hit:
>hsa-miR-7-5p     ENST00000625883.1 166.00      -23.92      2 22  1035 1055
      20    75.00%      85.00%


   Forward: Score: 166.000000  Q:2 to 22  R:1089 to 1109 Align Len (20)
(75.00%) (85.00%)

   Query:    3' ugUUGUUUUAGUGAUCAGAAGGu 5'
                  ||||||: :||  |||||||
   Ref:      5' ccAACAAAGGTAC--GTCTTCCa 3'

   Energy:  -23.570000 kCal/Mol

Scores for this hit:
```

```
>hsa-miR-7-5p     ENST00000625883.1 166.00      -23.57      2 22  1089 1109
      20    75.00%      85.00%


   Forward: Score: 166.000000  Q:2 to 22  R:1107 to 1127 Align Len (20)
(75.00%) (85.00%)

   Query:    3' ugUUGUUUUAGUGAUCAGAAGGu 5'
                  ||||||: :||  |||||||
   Ref:      5' ccAACAAAGGTAC--GTCTTCCa 3'

   Energy:  -23.570000 kCal/Mol

Scores for this hit:
>hsa-miR-7-5p     ENST00000625883.1 166.00      -23.57      2 22  1107 1127
      20    75.00%      85.00%


   Forward: Score: 166.000000  Q:2 to 22  R:1143 to 1163 Align Len (20)
(75.00%) (85.00%)

   Query:    3' ugUUGUUUUAGUGAUCAGAAGGu 5'
                  ||||||: :||  |||||||
   Ref:      5' ccAACAAAGGTAC--GTCTTCCa 3'

   Energy:  -23.570000 kCal/Mol

Scores for this hit:
>hsa-miR-7-5p     ENST00000625883.1 166.00      -23.57      2 22  1143 1163
      20    75.00%      85.00%


   Forward: Score: 164.000000  Q:2 to 20  R:690 to 712 Align Len (19)
(84.21%) (84.21%)

   Query:    3' uguuGUU-UUAGUGAUCAGAAGGu 5'
                    ||| |||| | ||||||||
   Ref:      5' ttccCAACAATC-CAAGTCTTCCg 3'

   Energy:  -21.799999 kCal/Mol

Scores for this hit:
>hsa-miR-7-5p     ENST00000625883.1 164.00      -21.80      2 20  690 712
      19    84.21%      84.21%


   Forward: Score: 164.000000  Q:2 to 19  R:980 to 1001 Align Len (17)
(82.35%) (88.24%)

   Query:    3' uguugUUUUAGUGAUCAGAAGGu 5'
                     |||||| | :|||||||
   Ref:      5' tccgaAAAATC-CAGGTCTTCCa 3'

   Energy:  -20.290001 kCal/Mol

Scores for this hit:
>hsa-miR-7-5p     ENST00000625883.1 164.00      -20.29      2 19  980 1001
      17    82.35%      88.24%
```

```
   Forward: Score: 163.000000  Q:2 to 22  R:157 to 178 Align Len (20)
(75.00%) (80.00%)

   Query:    3' ugUUGUUUUAGUGAUCAGAAGGu 5'
                  || ||: || | ||||||||
   Ref:      5' ccAAGAAGCTC-CAAGTCTTCCa 3'

   Energy:  -20.780001 kCal/Mol

Scores for this hit:
>hsa-miR-7-5p     ENST00000625883.1 163.00      -20.78      2 22  157 178
      20    75.00%      80.00%


   Forward: Score: 163.000000  Q:2 to 22  R:246 to 267 Align Len (20)
(75.00%) (80.00%)

   Query:    3' ugUUGUUUUAGUGAUCAGAAGGu 5'
                  |: |||||| |  |||||||
   Ref:      5' ccAGGAAAATC-CACGTCTTCCa 3'

   Energy:  -21.790001 kCal/Mol

Scores for this hit:
>hsa-miR-7-5p     ENST00000625883.1 163.00      -21.79      2 22  246 267
      20    75.00%      80.00%


   Forward: Score: 163.000000  Q:2 to 22  R:338 to 359 Align Len (20)
(75.00%) (80.00%)

   Query:    3' ugUUGUUUUAGUGAUCAGAAGGu 5'
                  |: ||||||  | |||||||
   Ref:      5' ccAGAAAAATCTGT-GTCTTCCa 3'

   Energy:  -20.490000 kCal/Mol

Scores for this hit:
>hsa-miR-7-5p     ENST00000625883.1 163.00      -20.49      2 22  338 359
      20    75.00%      80.00%


   Forward: Score: 162.000000  Q:2 to 18  R:175 to 195 Align Len (16)
(87.50%) (87.50%)

   Query:    3' uguuguUUUAGUGAUCAGAAGGu 5'
                      ||||||  ||||||||
   Ref:      5' tccagtAAATCA--AGTCTTCCa 3'

   Energy:  -21.190001 kCal/Mol

Scores for this hit:
>hsa-miR-7-5p     ENST00000625883.1 162.00      -21.19      2 18  175 195
      16    87.50%      87.50%


   Forward: Score: 162.000000  Q:2 to 21  R:618 to 639 Align Len (19)
(78.95%) (78.95%)

   Query:    3' uguUGUUUUAGUGAUCAGAAGGu 5'
                   | |||||| |  |||||||
```

```
   Ref:      5' tccAGAAAATC-CATGTCTTCCa 3'

   Energy:  -18.690001 kCal/Mol

Scores for this hit:
>hsa-miR-7-5p     ENST00000625883.1 162.00      -18.69      2 21  618 639
      19    78.95%      78.95%


   Forward: Score: 162.000000  Q:2 to 21  R:636 to 657 Align Len (19)
(78.95%) (78.95%)

   Query:    3' uguUGUUUUAGUGAUCAGAAGGu 5'
                   | |||||| |  |||||||
   Ref:      5' tccAGAAAATC-CATGTCTTCCa 3'

   Energy:  -18.690001 kCal/Mol

Scores for this hit:
>hsa-miR-7-5p     ENST00000625883.1 162.00      -18.69      2 21  636 657
      19    78.95%      78.95%


   Forward: Score: 162.000000  Q:2 to 21  R:673 to 694 Align Len (19)
(78.95%) (78.95%)

   Query:    3' uguUGUUUUAGUGAUCAGAAGGu 5'
                   | |||||| |  |||||||
   Ref:      5' tccAGAAAATC-CACGTCTTCCc 3'

   Energy:  -15.660000 kCal/Mol

Scores for this hit:
>hsa-miR-7-5p     ENST00000625883.1 162.00      -15.66      2 21  673 694
      19    78.95%      78.95%


   Forward: Score: 162.000000  Q:2 to 22  R:1017 to 1037 Align Len (20)
(75.00%) (80.00%)

   Query:    3' ugUUGUUUUAGUGAUCAGAAGGu 5'
                  |:||||  |||  |||||||
   Ref:      5' ccAGCAAATCCAC--GTCTTCCa 3'

   Energy:  -23.330000 kCal/Mol

Scores for this hit:
>hsa-miR-7-5p     ENST00000625883.1 162.00      -23.33      2 22  1017 1037
      20    75.00%      80.00%


   Forward: Score: 162.000000  Q:2 to 21  R:1052 to 1073 Align Len (19)
(73.68%) (84.21%)

   Query:    3' uguUGUUUUAGUGAUCAGAAGGu 5'
                   |: ||||:| | |||||||
   Ref:      5' tccATCAAATTAAT-GTCTTCCa 3'

   Energy:  -17.180000 kCal/Mol

Scores for this hit:
```

```
>hsa-miR-7-5p     ENST00000625883.1 162.00      -17.18      2 21  1052 1073
      19    73.68%      84.21%


   Forward: Score: 162.000000  Q:2 to 22  R:1125 to 1145 Align Len (20)
(70.00%) (85.00%)

   Query:    3' ugUUGUUUUAGUGAUCAGAAGGu 5'
                  ||||||: :|:  |||||||
   Ref:      5' ccAACAAAGGTAT--GTCTTCCa 3'

   Energy:  -22.129999 kCal/Mol

Scores for this hit:
>hsa-miR-7-5p     ENST00000625883.1 162.00      -22.13      2 22  1125 1145
      20    70.00%      85.00%


   Forward: Score: 162.000000  Q:2 to 21  R:1160 to 1181 Align Len (19)
(78.95%) (78.95%)

   Query:    3' uguUGUUUUAGUGAUCAGAAGGu 5'
                   | |||||| |  |||||||
   Ref:      5' tccAGAAAATC-CACGTCTTCCa 3'

   Energy:  -18.340000 kCal/Mol

Scores for this hit:
>hsa-miR-7-5p     ENST00000625883.1 162.00      -18.34      2 21  1160 1181
      19    78.95%      78.95%


   Forward: Score: 162.000000  Q:2 to 21  R:1196 to 1217 Align Len (19)
(78.95%) (78.95%)

   Query:    3' uguUGUUUUAGUGAUCAGAAGGu 5'
                   | |||||| |  |||||||
   Ref:      5' tccAGAAAATC-CACGTCTTCCa 3'

   Energy:  -18.340000 kCal/Mol

Scores for this hit:
>hsa-miR-7-5p     ENST00000625883.1 162.00      -18.34      2 21  1196 1217
      19    78.95%      78.95%


   Forward: Score: 162.000000  Q:2 to 22  R:1251 to 1271 Align Len (20)
(75.00%) (80.00%)

   Query:    3' ugUUGUUUUAGUGAUCAGAAGGu 5'
                  ||||||  ||:  |||||||
   Ref:      5' ccAACAAATCCAT--GTCTTCCt 3'

   Energy:  -19.770000 kCal/Mol

Scores for this hit:
>hsa-miR-7-5p     ENST00000625883.1 162.00      -19.77      2 22  1251 1271
      20    75.00%      80.00%
```

```
   Forward: Score: 162.000000  Q:2 to 20  R:1302 to 1325 Align Len (19)
(73.68%) (84.21%)

   Query:    3' uguuGUUUUAG-UGAUCAGAAGGu 5'
                    ||: ||| :|| |||||||
   Ref:      5' cttcCAGCATCTGCTCGTCTTCCa 3'

   Energy:  -20.459999 kCal/Mol

Scores for this hit:
>hsa-miR-7-5p     ENST00000625883.1 162.00      -20.46      2 20  1302 1325
      19    73.68%      84.21%


   Forward: Score: 162.000000  Q:2 to 20  R:1320 to 1343 Align Len (19)
(78.95%) (78.95%)

   Query:    3' uguuGUUUUAGUGAU-CAGAAGGu 5'
                    ||| ||| | | |||||||
   Ref:      5' cttcCAACATCTCCACGTCTTCCa 3'

   Energy:  -20.360001 kCal/Mol

Scores for this hit:
>hsa-miR-7-5p     ENST00000625883.1 162.00      -20.36      2 20  1320 1343
      19    78.95%      78.95%


   Forward: Score: 162.000000  Q:2 to 20  R:1338 to 1361 Align Len (19)
(73.68%) (84.21%)

   Query:    3' uguuGUUUUAGUGAU-CAGAAGGu 5'
                    ||: ||| ||: |||||||
   Ref:      5' cttcCAGCATCTCTGTGTCTTCCa 3'

   Energy:  -20.820000 kCal/Mol

Scores for this hit:
>hsa-miR-7-5p     ENST00000625883.1 162.00      -20.82      2 20  1338 1361
      19    73.68%      84.21%


   Forward: Score: 161.000000  Q:2 to 20  R:63 to 87 Align Len (20)
(80.00%) (80.00%)

   Query:    3' uguuGUU-UUAGUGAU-CAGAAGGu 5'
                    ||| |||| | | |||||||
   Ref:      5' cttcCAATAATCTCAAGGTCTTCCa 3'

   Energy:  -22.129999 kCal/Mol

Scores for this hit:
>hsa-miR-7-5p     ENST00000625883.1 161.00      -22.13      2 20  63 87 20
      80.00%      80.00%


   Forward: Score: 161.000000  Q:2 to 22  R:508 to 529 Align Len (21)
(76.19%) (80.95%)

   Query:    3' ugUUGUUUUAGUGAU-CAGAAGGu 5'
                  |: |||||  ||| |||||||
```

```
   Ref:      5' ccAGAAAAAT--CTACGTCTTCCa 3'

   Energy:  -19.760000 kCal/Mol

Scores for this hit:
>hsa-miR-7-5p     ENST00000625883.1 161.00      -19.76      2 22  508 529
      21    76.19%      80.95%


   Forward: Score: 159.000000  Q:2 to 22  R:193 to 212 Align Len (20)
(80.00%) (85.00%)

   Query:    3' ugUUGUUUUAGUGAUCAGAAGGu 5'
                  |:| ||||| | ||||||||
   Ref:      5' ccAGC-AAATC-C-AGTCTTCCa 3'

   Energy:  -23.430000 kCal/Mol

Scores for this hit:
>hsa-miR-7-5p     ENST00000625883.1 159.00      -23.43      2 22  193 212
      20    80.00%      85.00%


   Forward: Score: 159.000000  Q:2 to 22  R:283 to 304 Align Len (20)
(70.00%) (80.00%)

   Query:    3' ugUUGUUUUAGUGAUCAGAAGGu 5'
                  ||:||  |||  :|||||||
   Ref:      5' ccAATAATTTCA-AGGTCTTCCa 3'

   Energy:  -22.610001 kCal/Mol

Scores for this hit:
>hsa-miR-7-5p     ENST00000625883.1 159.00      -22.61      2 22  283 304
      20    70.00%      80.00%


   Forward: Score: 159.000000  Q:2 to 22  R:655 to 676 Align Len (20)
(70.00%) (80.00%)

   Query:    3' ugUUGUUUUAGUGAUCAGAAGGu 5'
                  |::||  || | ||||||||
   Ref:      5' ccAGTAACCTC-CCAGTCTTCCa 3'

   Energy:  -20.129999 kCal/Mol

Scores for this hit:
>hsa-miR-7-5p     ENST00000625883.1 159.00      -20.13      2 22  655 676
      20    70.00%      80.00%


   Forward: Score: 159.000000  Q:2 to 22  R:1377 to 1398 Align Len (20)
(75.00%) (75.00%)

   Query:    3' ugUUGUUUUAGUGAUCAGAAGGu 5'
                  ||| || |  | ||||||||
   Ref:      5' ccAAC-AACTACCCAGTCTTCCa 3'

   Energy:  -21.980000 kCal/Mol

Scores for this hit:
```

```
>hsa-miR-7-5p     ENST00000625883.1 159.00      -21.98      2 22  1377 1398
      20    75.00%      75.00%


   Forward: Score: 158.000000  Q:2 to 22  R:48 to 68 Align Len (20)
(70.00%) (80.00%)

   Query:    3' ugUUGUUUUAGUGAUCAGAAGGu 5'
                  |:|:|  |||  ||||||||
   Ref:      5' ccAGCGACTTCA--AGTCTTCCa 3'

   Energy:  -22.990000 kCal/Mol

Scores for this hit:
>hsa-miR-7-5p     ENST00000625883.1 158.00      -22.99      2 22  48 68 20
      70.00%      80.00%


   Forward: Score: 158.000000  Q:2 to 21  R:264 to 285 Align Len (19)
(73.68%) (78.95%)

   Query:    3' uguUGUUUUAGUGAUCAGAAGGu 5'
                   | :||||| |  |||||||
   Ref:      5' tccAGGAAATC-CATGTCTTCCa 3'

   Energy:  -18.450001 kCal/Mol

Scores for this hit:
>hsa-miR-7-5p     ENST00000625883.1 158.00      -18.45      2 21  264 285
      19    73.68%      78.95%


   Forward: Score: 158.000000  Q:2 to 17  R:411 to 432 Align Len (15)
(86.67%) (86.67%)

   Query:    3' uguuguuUUAGUGAUCAGAAGGu 5'
                       ||||| | |||||||
   Ref:      5' tccagtcAATCAGT-GTCTTCCa 3'

   Energy:  -19.219999 kCal/Mol

Scores for this hit:
>hsa-miR-7-5p     ENST00000625883.1 158.00      -19.22      2 17  411 432
      15    86.67%      86.67%


   Forward: Score: 158.000000  Q:2 to 17  R:450 to 471 Align Len (15)
(86.67%) (86.67%)

   Query:    3' uguuguuUUAGUGAUCAGAAGGu 5'
                       ||||| | |||||||
   Ref:      5' tccagtcAATCAGT-GTCTTCCa 3'

   Energy:  -19.219999 kCal/Mol

Scores for this hit:
>hsa-miR-7-5p     ENST00000625883.1 158.00      -19.22      2 17  450 471
      15    86.67%      86.67%
```

```
   Forward: Score: 158.000000  Q:2 to 19  R:727 to 748 Align Len (18)
(83.33%) (83.33%)

   Query:    3' uguugUUUUAGUGAU-CAGAAGGu 5'
                     |||||  ||| |||||||
   Ref:      5' tcctgAAAAT--CTACGTCTTCCa 3'

   Energy:  -17.200001 kCal/Mol

Scores for this hit:
>hsa-miR-7-5p     ENST00000625883.1 158.00      -17.20      2 19  727 748
      18    83.33%      83.33%


   Forward: Score: 158.000000  Q:2 to 20  R:743 to 766 Align Len (19)
(73.68%) (78.95%)

   Query:    3' uguuGUUUUAGUGAU-CAGAAGGu 5'
                    |||||  :| | |||||||
   Ref:      5' cttcCAAAAAAGCCATGTCTTCCa 3'

   Energy:  -17.580000 kCal/Mol

Scores for this hit:
>hsa-miR-7-5p     ENST00000625883.1 158.00      -17.58      2 20  743 766
      19    73.68%      78.95%


   Forward: Score: 158.000000  Q:2 to 21  R:872 to 893 Align Len (19)
(73.68%) (78.95%)

   Query:    3' uguUGUUUUAGUGAUCAGAAGGu 5'
                   | :||||| |  |||||||
   Ref:      5' tccAGGAAATC-CGTGTCTTCCa 3'

   Energy:  -19.049999 kCal/Mol

Scores for this hit:
>hsa-miR-7-5p     ENST00000625883.1 158.00      -19.05      2 21  872 893
      19    73.68%      78.95%


   Forward: Score: 158.000000  Q:2 to 22  R:891 to 911 Align Len (20)
(70.00%) (80.00%)

   Query:    3' ugUUGUUUUAGUGAUCAGAAGGu 5'
                  |:|||:  |||  |||||||
   Ref:      5' ccAGCAAGTCCAC--GTCTTCCa 3'

   Energy:  -23.900000 kCal/Mol

Scores for this hit:
>hsa-miR-7-5p     ENST00000625883.1 158.00      -23.90      2 22  891 911
      20    70.00%      80.00%


   Forward: Score: 158.000000  Q:2 to 21  R:998 to 1019 Align Len (19)
(73.68%) (78.95%)

   Query:    3' uguUGUUUUAGUGAUCAGAAGGu 5'
                   | :||||| |  |||||||
```

```
   Ref:      5' tccAGGAAATC-CGTGTCTTCCa 3'

   Energy:  -19.049999 kCal/Mol

Scores for this hit:
>hsa-miR-7-5p     ENST00000625883.1 158.00      -19.05      2 21  998 1019
      19    73.68%      78.95%


   Forward: Score: 158.000000  Q:2 to 22  R:1179 to 1199 Align Len (20)
(70.00%) (80.00%)

   Query:    3' ugUUGUUUUAGUGAUCAGAAGGu 5'
                 ||| ||: ||:  |||||||
   Ref:      5' ccAACCAAGCCAT--GTCTTCCa 3'

   Energy:  -19.420000 kCal/Mol

Scores for this hit:
>hsa-miR-7-5p     ENST00000625883.1 158.00      -19.42      2 22  1179 1199
      20    70.00%      80.00%


   Forward: Score: 158.000000  Q:2 to 21  R:1214 to 1235 Align Len (19)
(73.68%) (78.95%)

   Query:    3' uguUGUUUUAGUGAUCAGAAGGu 5'
                  | ||||| |:  |||||||
   Ref:      5' tccAGAAAAT-ATATGTCTTCCa 3'

   Energy:  -16.440001 kCal/Mol

Scores for this hit:
>hsa-miR-7-5p     ENST00000625883.1 158.00      -16.44      2 21  1214 1235
      19    73.68%      78.95%


   Forward: Score: 158.000000  Q:2 to 22  R:1233 to 1253 Align Len (20)
(70.00%) (80.00%)

   Query:    3' ugUUGUUUUAGUGAUCAGAAGGu 5'
                 ||| ||: :||  |||||||
   Ref:      5' ccAACTAAGCTAC--GTCTTCCa 3'

   Energy:  -19.959999 kCal/Mol

Scores for this hit:
>hsa-miR-7-5p     ENST00000625883.1 158.00      -19.96      2 22  1233 1253
      20    70.00%      80.00%


   Forward: Score: 157.000000  Q:2 to 22  R:30 to 50 Align Len (20)
(75.00%) (80.00%)

   Query:    3' ugUUGUUUUAGUGAUCAGAAGGu 5'
                 ||| || || | :|||||||
   Ref:      5' ccAAC-AACTC-CGGGTCTTCCa 3'

   Energy:  -21.260000 kCal/Mol

Scores for this hit:
```

```
>hsa-miR-7-5p     ENST00000625883.1 157.00      -21.26      2 22  30 50 20
      75.00%      80.00%


   Forward: Score: 157.000000  Q:2 to 20  R:1405 to 1426 Align Len (18)
(77.78%) (77.78%)

   Query:    3' uguuGUUUUAGUGAUCAGAAGGu 5'
                    ||| ||| |  |||||||
   Ref:      5' ggctCAATATC-CATGTCTTCCa 3'

   Energy:  -17.150000 kCal/Mol

Scores for this hit:
>hsa-miR-7-5p     ENST00000625883.1 157.00      -17.15      2 20  1405 1426
      18    77.78%      77.78%


   Forward: Score: 156.000000  Q:2 to 19  R:600 to 621 Align Len (17)
(76.47%) (82.35%)

   Query:    3' uguugUUUUAGUGAUCAGAAGGu 5'
                     ||:||| |  |||||||
   Ref:      5' tcctgAAGATC-CACGTCTTCCa 3'

   Energy:  -17.320000 kCal/Mol

Scores for this hit:
>hsa-miR-7-5p     ENST00000625883.1 156.00      -17.32      2 19  600 621
      17    76.47%      82.35%


   Forward: Score: 154.000000  Q:2 to 21  R:120 to 141 Align Len (19)
(73.68%) (73.68%)

   Query:    3' uguUGUUUUAGUGAUCAGAAGGu 5'
                   | | |||| |  |||||||
   Ref:      5' tccAGACAATC-CATGTCTTCCg 3'

   Energy:  -17.299999 kCal/Mol

Scores for this hit:
>hsa-miR-7-5p     ENST00000625883.1 154.00      -17.30      2 21  120 141
      19    73.68%      73.68%


   Forward: Score: 154.000000  Q:2 to 17  R:489 to 510 Align Len (15)
(80.00%) (86.67%)

   Query:    3' uguuguuUUAGUGAUCAGAAGGu 5'
                       |:||| | |||||||
   Ref:      5' tccagtcAGTCAGT-GTCTTCCa 3'

   Energy:  -19.430000 kCal/Mol

Scores for this hit:
>hsa-miR-7-5p     ENST00000625883.1 154.00      -19.43      2 17  489 510
      15    80.00%      86.67%
```

```
   Forward: Score: 154.000000  Q:2 to 20  R:1266 to 1289 Align Len (19)
(73.68%) (73.68%)

   Query:    3' uguuGUUUUAGUGAU-CAGAAGGu 5'
                    | | ||| | | |||||||
   Ref:      5' cttcCTATATCTCCAGGTCTTCCa 3'

   Energy:  -15.270000 kCal/Mol

Scores for this hit:
>hsa-miR-7-5p     ENST00000625883.1 154.00      -15.27      2 20  1266 1289
      19    73.68%      73.68%


   Forward: Score: 154.000000  Q:2 to 20  R:1356 to 1379 Align Len (19)
(68.42%) (78.95%)

   Query:    3' uguuGUUUUAGUGAU-CAGAAGGu 5'
                    ||: ||| : | |||||||
   Ref:      5' cttcCAGCATCTTCATGTCTTCCa 3'

   Energy:  -20.930000 kCal/Mol

Scores for this hit:
>hsa-miR-7-5p     ENST00000625883.1 154.00      -20.93      2 20  1356 1379
      19    68.42%      78.95%


   Forward: Score: 152.000000  Q:2 to 19  R:138 to 159 Align Len (17)
(76.47%) (76.47%)

   Query:    3' uguugUUUUAGUGAUCAGAAGGu 5'
                     | |||| |  |||||||
   Ref:      5' tccggACAATC-CATGTCTTCCa 3'

   Energy:  -16.920000 kCal/Mol

Scores for this hit:
>hsa-miR-7-5p     ENST00000625883.1 152.00      -16.92      2 19  138 159
      17    76.47%      76.47%


   Forward: Score: 151.000000  Q:2 to 22  R:782 to 803 Align Len (20)
(60.00%) (80.00%)

   Query:    3' ugUUGUUUUAGUGAUCAGAAGGu 5'
                  ||:::  || | :|||||||
   Ref:      5' ccAATGGCCTC-CAGGTCTTCCa 3'

   Energy:  -17.180000 kCal/Mol

Scores for this hit:
>hsa-miR-7-5p     ENST00000625883.1 151.00      -17.18      2 22  782 803
      20    60.00%      80.00%


   Forward: Score: 150.000000  Q:2 to 17  R:319 to 340 Align Len (15)
(80.00%) (80.00%)

   Query:    3' uguuguuUUAGUGAUCAGAAGGu 5'
                       |||| |  |||||||
```

```
   Ref:      5' tccagctAATC-CATGTCTTCCa 3'

   Energy:  -19.430000 kCal/Mol

Scores for this hit:
>hsa-miR-7-5p     ENST00000625883.1 150.00      -19.43      2 17  319 340
      15    80.00%      80.00%


   Forward: Score: 150.000000  Q:2 to 20  R:706 to 730 Align Len (20)
(60.00%) (80.00%)

   Query:    3' uguuGUUUUAGUGA--UCAGAAGGu 5'
                    | ::|| |:|  :|||||||
   Ref:      5' tcttCCGGATAATTTGGGTCTTCCt 3'

   Energy:  -15.170000 kCal/Mol

Scores for this hit:
>hsa-miR-7-5p     ENST00000625883.1 150.00      -15.17      2 20  706 730
      20    60.00%      80.00%


   Forward: Score: 150.000000  Q:2 to 20  R:798 to 821 Align Len (19)
(68.42%) (73.68%)

   Query:    3' uguuGUUUUAGUGAU-CAGAAGGu 5'
                    ||:| |  | | |||||||
   Ref:      5' cttcCAGACTATCCATGTCTTCCa 3'

   Energy:  -16.629999 kCal/Mol

Scores for this hit:
>hsa-miR-7-5p     ENST00000625883.1 150.00      -16.63      2 20  798 821
      19    68.42%      73.68%


   Forward: Score: 150.000000  Q:2 to 20  R:924 to 947 Align Len (19)
(68.42%) (73.68%)

   Query:    3' uguuGUUUUAGUGAU-CAGAAGGu 5'
                    ||:| |  | | |||||||
   Ref:      5' cttcCAGACTATCCATGTCTTCCa 3'

   Energy:  -16.629999 kCal/Mol

Scores for this hit:
>hsa-miR-7-5p     ENST00000625883.1 150.00      -16.63      2 20  924 947
      19    68.42%      73.68%


   Forward: Score: 149.000000  Q:2 to 20  R:1446 to 1467 Align Len (18)
(61.11%) (83.33%)

   Query:    3' uguuGUUUUAGUGAUCAGAAGGu 5'
                    ::| ||: | :|||||||
   Ref:      5' cttcTGACATT-CAGGTCTTCCa 3'

   Energy:  -17.250000 kCal/Mol

Scores for this hit:
```

```
>hsa-miR-7-5p     ENST00000625883.1 149.00      -17.25      2 20  1446 1467
      18    61.11%      83.33%


   Forward: Score: 147.000000  Q:2 to 22  R:11 to 32 Align Len (20)
(60.00%) (75.00%)

   Query:    3' ugUUGUUUUAGUGAUCAGAAGGu 5'
                  | | : :|||  :|||||||
   Ref:      5' gcACCTGTGTCA-AGGTCTTCCa 3'

   Energy:  -18.840000 kCal/Mol

Scores for this hit:
>hsa-miR-7-5p     ENST00000625883.1 147.00      -18.84      2 22  11 32 20
      60.00%      75.00%


   Forward: Score: 146.000000  Q:2 to 22  R:583 to 603 Align Len (20)
(60.00%) (75.00%)

   Query:    3' ugUUGUUUUAGUGAUCAGAAGGu 5'
                  |:| ||  :|:  |||||||
   Ref:      5' ccAGCCAA--TATATGTCTTCCt 3'

   Energy:  -13.990000 kCal/Mol

Scores for this hit:
>hsa-miR-7-5p     ENST00000625883.1 146.00      -13.99      2 22  583 603
      20    60.00%      75.00%


   Forward: Score: 142.000000  Q:2 to 20  R:1067 to 1091 Align Len (20)
(60.00%) (70.00%)

   Query:    3' uguuGUUUUAGUGA--UCAGAAGGu 5'
                    | |:  :|||   |||||||
   Ref:      5' tcttCCAGCCTACTTGTGTCTTCCa 3'

   Energy:  -18.230000 kCal/Mol

Scores for this hit:
>hsa-miR-7-5p     ENST00000625883.1 142.00      -18.23      2 20  1067 1091
      20    60.00%      70.00%

Score for this Scan:
Seq1,Seq2,Tot Score,Tot Energy,Max Score,Max
Energy,Strand,Len1,Len2,Positions
>>hsa-miR-7-5p    ENST00000625883.1 10697.00    -1323.67    174.00      -
28.27 108   23    1485   207 356 818 944 432 471 564 526 854 909 1035 1089
1107 1143 690 980 157 246 338 175 618 636 673 1017 1052 1125 1160 1196 1251
1302 1320 1338 63 508 193 283 655 1377 48 264 411 450 727 743 872 891 998
1179 1214 1233 30 1405 600 120 489 1266 1356 138 782 319 706 798 924 1446
11 583 1067
Complete
```

Putative binding sites of circSTS (chrX:7,514,882-7,516,290) predicted by miRanda

```
Read Sequence:hsa-miR-5584-5p (22 nt)
Read Sequence:X:7514882|7516290 (1409 nt)
=-=-=-=-=-=-=-=-=-=-=-=-=-=-=-=-=-=-=-=-=-=-=-=-=-=-=-=-=-=-=-=-=
Performing Scan: hsa-miR-5584-5p vs X:7514882|7516290
=-=-=-=-=-=-=-=-=-=-=-=-=-=-=-=-=-=-=-=-=-=-=-=-=-=-=-=-=-=-=-=-=

   Forward: Score: 166.000000  Q:2 to 21  R:774 to 794 Align Len (19)
(78.95%) (84.21%)

   Query:    3' agAUCAAGAAGGGUAAAGGGAc 5'
                  || |||  |:|||||||||
   Ref:      5' ggTA-TTCAACTCATTTCCCTg 3'

   Energy:  -23.950001 kCal/Mol

Scores for this hit:
>hsa-miR-5584-5p  X:7514882|7516290 166.00      -23.95      2 21  774 794
      19    78.95%      84.21%


   Forward: Score: 160.000000  Q:2 to 17  R:623 to 644 Align Len (15)
(80.00%) (86.67%)

   Query:    3' agaucaAGAAGGGUAAAGGGAc 5'
                      ||  |:|||||||||
   Ref:      5' gggtacTCAACTCATTTCCCTg 3'

   Energy:  -23.490000 kCal/Mol

Scores for this hit:
>hsa-miR-5584-5p  X:7514882|7516290 160.00      -23.49      2 17  623 644
      15    80.00%      86.67%


   Forward: Score: 158.000000  Q:2 to 21  R:744 to 764 Align Len (19)
(68.42%) (84.21%)

   Query:    3' agAUCAAGAAGGGUAAAGGGAc 5'
                  || |||  |:::|||||||
   Ref:      5' caTA-TTCAACTTGTTTCCCTg 3'

   Energy:  -20.540001 kCal/Mol

Scores for this hit:
>hsa-miR-5584-5p  X:7514882|7516290 158.00      -20.54      2 21  744 764
      19    68.42%      84.21%


   Forward: Score: 158.000000  Q:2 to 21  R:836 to 856 Align Len (19)
(73.68%) (78.95%)

   Query:    3' agAUCAAGAAGGGUAAAGGGAc 5'
                  || |||  |:| |||||||
   Ref:      5' ggTA-TTCAACTCTTTTCCCTg 3'

   Energy:  -19.040001 kCal/Mol
```

```
Scores for this hit:
>hsa-miR-5584-5p  X:7514882|7516290 158.00      -19.04      2 21  836 856
      19    73.68%      78.95%


   Forward: Score: 158.000000  Q:2 to 21  R:1279 to 1303 Align Len (22)
(72.73%) (77.27%)

   Query:    3' agAUCAAGAAG--GGU-AAAGGGAc 5'
                  ||| || ||  |:| |||||||
   Ref:      5' ccTAGATCATCAACTATTTTCCCTg 3'

   Energy:  -20.660000 kCal/Mol

Scores for this hit:
>hsa-miR-5584-5p  X:7514882|7516290 158.00      -20.66      2 21  1279 1303
      22    72.73%      77.27%


   Forward: Score: 156.000000  Q:2 to 13  R:653 to 674 Align Len (11)
(90.91%) (100.00%)

   Query:    3' agaucaagaaGGGUAAAGGGAc 5'
                          |:|||||||||
   Ref:      5' tggtacccaaCTCATTTCCCTg 3'

   Energy:  -22.360001 kCal/Mol

Scores for this hit:
>hsa-miR-5584-5p  X:7514882|7516290 156.00      -22.36      2 13  653 674
      11    90.91%      100.00%


   Forward: Score: 156.000000  Q:2 to 17  R:1223 to 1244 Align Len (15)
(73.33%) (86.67%)

   Query:    3' agaucaAGAAGGGUAAAGGGAc 5'
                      ||| ::| |||||||
   Ref:      5' cggtcaTCTCTTCTTTTCCCTg 3'

   Energy:  -19.570000 kCal/Mol

Scores for this hit:
>hsa-miR-5584-5p  X:7514882|7516290 156.00      -19.57      2 17  1223 1244
      15    73.33%      86.67%


   Forward: Score: 155.000000  Q:2 to 16  R:1117 to 1138 Align Len (14)
(71.43%) (92.86%)

   Query:    3' agaucaaGAAGGGUAAAGGGAc 5'
                       || :::||||||||
   Ref:      5' tcatcaaCTATTTATTTCCCTt 3'

   Energy:  -15.740000 kCal/Mol

Scores for this hit:
>hsa-miR-5584-5p  X:7514882|7516290 155.00      -15.74      2 16  1117 1138
      14    71.43%      92.86%
```

```
   Forward: Score: 151.000000  Q:2 to 21  R:711 to 734 Align Len (21)
(61.90%) (76.19%)

   Query:    3' agAUCA--AGAAGGGUAAAGGGAc 5'
                  |:||  ||  |: :|||||||
   Ref:      5' ctTGGTACTCAGCTGGTTTCCCTg 3'

   Energy:  -22.190001 kCal/Mol

Scores for this hit:
>hsa-miR-5584-5p  X:7514882|7516290 151.00      -22.19      2 21  711 734
      21    61.90%      76.19%


   Forward: Score: 148.000000  Q:2 to 17  R:683 to 704 Align Len (15)
(66.67%) (80.00%)

   Query:    3' agaucaAGAAGGGUAAAGGGAc 5'
                      ||  |: :|||||||
   Ref:      5' cggtacTCAACTGGTTTCCCTg 3'

   Energy:  -18.450001 kCal/Mol

Scores for this hit:
>hsa-miR-5584-5p  X:7514882|7516290 148.00      -18.45      2 17  683 704
      15    66.67%      80.00%


   Forward: Score: 147.000000  Q:2 to 21  R:471 to 494 Align Len (21)
(61.90%) (71.43%)

   Query:    3' agAUCA--AGAAGGGUAAAGGGAc 5'
                  |:||  ||  |:  |||||||
   Ref:      5' ttTGGTACTCAACTGCTTTCCCTg 3'

   Energy:  -17.809999 kCal/Mol

Scores for this hit:
>hsa-miR-5584-5p  X:7514882|7516290 147.00      -17.81      2 21  471 494
      21    61.90%      71.43%


   Forward: Score: 146.000000  Q:2 to 20  R:66 to 89 Align Len (20)
(65.00%) (70.00%)

   Query:    3' agaUCAAGAA--GGGUAAAGGGAc 5'
                   |||  ||  |:  |||||||
   Ref:      5' ctcAGTCATTGACTGCTTTCCCTg 3'

   Energy:  -17.650000 kCal/Mol

Scores for this hit:
>hsa-miR-5584-5p  X:7514882|7516290 146.00      -17.65      2 20  66 89 20
      65.00%      70.00%


   Forward: Score: 144.000000  Q:2 to 17  R:1252 to 1273 Align Len (15)
(66.67%) (73.33%)

   Query:    3' agaucaAGAAGGGUAAAGGGAc 5'
```

```
                   ||   :| |||||||
   Ref:      5' gtgtaaTCACGTCTTTTCCCTg 3'

   Energy:  -16.160000 kCal/Mol

Scores for this hit:
>hsa-miR-5584-5p  X:7514882|7516290 144.00      -16.16      2 17  1252 1273
      15    66.67%      73.33%


   Forward: Score: 143.000000  Q:2 to 21  R:381 to 404 Align Len (21)
(57.14%) (71.43%)

   Query:    3' agAUCA--AGAAGGGUAAAGGGAc 5'
                  |:||  ||  ::  |||||||
   Ref:      5' ctTGGTGGTCAATTATTTTCCCTg 3'

   Energy:  -17.870001 kCal/Mol

Scores for this hit:
>hsa-miR-5584-5p  X:7514882|7516290 143.00      -17.87      2 21  381 404
      21    57.14%      71.43%


   Forward: Score: 142.000000  Q:2 to 17  R:1191 to 1214 Align Len (17)
(70.59%) (76.47%)

   Query:    3' agaucaAGAAG-GGU-AAAGGGAc 5'
                      || ||  :| |||||||
   Ref:      5' tttttgTCATCAGTATTTTCCCTg 3'

   Energy:  -16.450001 kCal/Mol

Scores for this hit:
>hsa-miR-5584-5p  X:7514882|7516290 142.00      -16.45      2 17  1191 1214
      17    70.59%      76.47%


   Forward: Score: 140.000000  Q:2 to 9  R:948 to 969 Align Len (7)
(100.00%) (100.00%)

   Query:    3' agaucaagaaggguAAAGGGAc 5'
                              |||||||
   Ref:      5' cctatcatcaatgcTTTCCCTg 3'

   Energy:  -14.560000 kCal/Mol

Scores for this hit:
>hsa-miR-5584-5p  X:7514882|7516290 140.00      -14.56      2 9   948 969
      7     100.00%     100.00%

Score for this Scan:
Seq1,Seq2,Tot Score,Tot Energy,Max Score,Max
Energy,Strand,Len1,Len2,Positions
>>hsa-miR-5584-5p X:7514882|7516290 2428.00     -306.49     166.00      -
23.95 1976  22    1409   774 623 744 836 1279 653 1223 1117 711 683 471 66
1252 381 1191 948
Complete
```

```
Read Sequence:hsa-miR-7113-5p (21 nt)
Read Sequence:X:7514882|7516290 (1409 nt)
=-=-=-=-=-=-=-=-=-=-=-=-=-=-=-=-=-=-=-=-=-=-=-=-=-=-=-=-=-=-=-=-=
Performing Scan: hsa-miR-7113-5p vs X:7514882|7516290
=-=-=-=-=-=-=-=-=-=-=-=-=-=-=-=-=-=-=-=-=-=-=-=-=-=-=-=-=-=-=-=-=

   Forward: Score: 162.000000  Q:2 to 20  R:70 to 91 Align Len (19)
(73.68%) (84.21%)

   Query:    3' gaGUGUGUGAC-AGAGGGACCu 5'
                  ||:  |||| |:|||||||
   Ref:      5' gtCATTGACTGCTTTCCCTGGa 3'

   Energy:  -28.100000 kCal/Mol

Scores for this hit:
>hsa-miR-7113-5p  X:7514882|7516290 162.00      -28.10      2 20  70 91 19
      73.68%      84.21%


   Forward: Score: 162.000000  Q:2 to 20  R:445 to 466 Align Len (19)
(73.68%) (84.21%)

   Query:    3' gaGUGUGUGACAG-AGGGACCu 5'
                  ||:  |||||: |||||||
   Ref:      5' gtCATCGACTGTTGTCCCTGGa 3'

   Energy:  -27.709999 kCal/Mol

Scores for this hit:
>hsa-miR-7113-5p  X:7514882|7516290 162.00      -27.71      2 20  445 466
      19    73.68%      84.21%


   Forward: Score: 162.000000  Q:2 to 20  R:534 to 555 Align Len (19)
(73.68%) (84.21%)

   Query:    3' gaGUGUGUGACA-GAGGGACCu 5'
                  ||:  ||||| :|||||||
   Ref:      5' gtCATTGACTGTGTTCCCTGGa 3'

   Energy:  -28.100000 kCal/Mol

Scores for this hit:
>hsa-miR-7113-5p  X:7514882|7516290 162.00      -28.10      2 20  534 555
      19    73.68%      84.21%


   Forward: Score: 162.000000  Q:2 to 20  R:1150 to 1171 Align Len (19)
(73.68%) (84.21%)

   Query:    3' gaGUGUGUGACA-GAGGGACCu 5'
                  || | :|||| :|||||||
   Ref:      5' gtCAGAGGCTGTGTTCCCTGGg 3'

   Energy:  -28.340000 kCal/Mol

Scores for this hit:
>hsa-miR-7113-5p  X:7514882|7516290 162.00      -28.34      2 20  1150 1171
      19    73.68%      84.21%
```

```
   Forward: Score: 161.000000  Q:2 to 20  R:474 to 496 Align Len (20)
(75.00%) (85.00%)

   Query:    3' gaGUGUG-UGAC-AGAGGGACCu 5'
                 :|| | |||| |:|||||||
   Ref:      5' ggTACTCAACTGCTTTCCCTGGa 3'

   Energy:  -28.370001 kCal/Mol

Scores for this hit:
>hsa-miR-7113-5p  X:7514882|7516290 161.00      -28.37      2 20  474 496
      20    75.00%      85.00%


   Forward: Score: 161.000000  Q:2 to 20  R:684 to 706 Align Len (20)
(75.00%) (85.00%)

   Query:    3' gaGUGUG-UGA-CAGAGGGACCu 5'
                 :|| | ||| ||:|||||||
   Ref:      5' ggTACTCAACTGGTTTCCCTGGa 3'

   Energy:  -28.370001 kCal/Mol

Scores for this hit:
>hsa-miR-7113-5p  X:7514882|7516290 161.00      -28.37      2 20  684 706
      20    75.00%      85.00%


   Forward: Score: 161.000000  Q:2 to 20  R:714 to 736 Align Len (20)
(75.00%) (85.00%)

   Query:    3' gaGUGUGU-GA-CAGAGGGACCu 5'
                 :|| || || ||:|||||||
   Ref:      5' ggTACTCAGCTGGTTTCCCTGGa 3'

   Energy:  -28.040001 kCal/Mol

Scores for this hit:
>hsa-miR-7113-5p  X:7514882|7516290 161.00      -28.04      2 20  714 736
      20    75.00%      85.00%


   Forward: Score: 161.000000  Q:2 to 18  R:746 to 766 Align Len (16)
(75.00%) (87.50%)

   Query:    3' gaguGUGUGACAGAGGGACCu 5'
                   ||  :|||:|||||||
   Ref:      5' tattCAACTTGTTTCCCTGGa 3'

   Energy:  -27.809999 kCal/Mol

Scores for this hit:
>hsa-miR-7113-5p  X:7514882|7516290 161.00      -27.81      2 18  746 766
      16    75.00%      87.50%


   Forward: Score: 157.000000  Q:2 to 18  R:1255 to 1275 Align Len (16)
(68.75%) (87.50%)

   Query:    3' gaguGUGUGACAGAGGGACCu 5'
```

```
                 |||::  |:|||||||
   Ref:      5' taatCACGTCTTTTCCCTGGa 3'

   Energy:  -26.290001 kCal/Mol

Scores for this hit:
>hsa-miR-7113-5p  X:7514882|7516290 157.00      -26.29      2 18  1255 1275
      16    68.75%      87.50%


   Forward: Score: 156.000000  Q:2 to 20  R:951 to 971 Align Len (19)
(73.68%) (84.21%)

   Query:    3' gaGUGUGUGAC-AGAGGGACCu 5'
                 ||: || || |:|||||||
   Ref:      5' atCAT-CAATGCTTTCCCTGGa 3'

   Energy:  -24.270000 kCal/Mol

Scores for this hit:
>hsa-miR-7113-5p  X:7514882|7516290 156.00      -24.27      2 20  951 971
      19    73.68%      84.21%


   Forward: Score: 155.000000  Q:2 to 20  R:840 to 858 Align Len (18)
(77.78%) (83.33%)

   Query:    3' gaGUGUGUGACAGAGGGACCu 5'
                 || || || |:|||||||
   Ref:      5' ttCA-ACTCT-TTTCCCTGGa 3'

   Energy:  -23.020000 kCal/Mol

Scores for this hit:
>hsa-miR-7113-5p  X:7514882|7516290 155.00      -23.02      2 20  840 858
      18    77.78%      83.33%


   Forward: Score: 154.000000  Q:2 to 20  R:1284 to 1305 Align Len (19)
(68.42%) (78.95%)

   Query:    3' gaGUGUGUGA-CAGAGGGACCu 5'
                 ||:  |||  |:|||||||
   Ref:      5' atCATCAACTATTTTCCCTGGa 3'

   Energy:  -23.100000 kCal/Mol

Scores for this hit:
>hsa-miR-7113-5p  X:7514882|7516290 154.00      -23.10      2 20  1284 1305
      19    68.42%      78.95%


   Forward: Score: 153.000000  Q:2 to 20  R:624 to 646 Align Len (20)
(70.00%) (80.00%)

   Query:    3' gaGUGUG-UGA-CAGAGGGACCu 5'
                 :|| | |||  |:|||||||
   Ref:      5' ggTACTCAACTCATTTCCCTGGa 3'

   Energy:  -24.490000 kCal/Mol
```

```
Scores for this hit:
>hsa-miR-7113-5p  X:7514882|7516290 153.00      -24.49      2 20  624 646
      20    70.00%      80.00%


   Forward: Score: 150.000000  Q:2 to 18  R:776 to 796 Align Len (17)
(76.47%) (82.35%)

   Query:    3' gaguGUGUGA-CAGAGGGACCu 5'
                    || |||  |:|||||||
   Ref:      5' tattCA-ACTCATTTCCCTGGa 3'

   Energy:  -21.910000 kCal/Mol

Scores for this hit:
>hsa-miR-7113-5p  X:7514882|7516290 150.00      -21.91      2 18  776 796
      17    76.47%      82.35%

   Forward: Score: 148.000000  Q:2 to 20  R:1196 to 1216 Align Len (19)
(68.42%) (78.95%)

   Query:    3' gaGUGUGUGA-CAGAGGGACCu 5'
                  ||: || |  |:|||||||
   Ref:      5' gtCAT-CAGTATTTTCCCTGGa 3'

   Energy:  -21.540001 kCal/Mol

Scores for this hit:
>hsa-miR-7113-5p  X:7514882|7516290 148.00      -21.54      2 20  1196 1216
      19    68.42%      78.95%

   Forward: Score: 146.000000  Q:2 to 20  R:1314 to 1335 Align Len (19)
(57.89%) (78.95%)

   Query:    3' gaGUGUGUGACA-GAGGGACCu 5'
                  :|:  |:| | :|||||||
   Ref:      5' gtTATTGATTATGTTCCCTGGa 3'

   Energy:  -19.480000 kCal/Mol

Scores for this hit:
>hsa-miR-7113-5p  X:7514882|7516290 146.00      -19.48      2 20  1314 1335
      19    57.89%      78.95%

Score for this Scan:
Seq1,Seq2,Tot Score,Tot Energy,Max Score,Max
Energy,Strand,Len1,Len2,Positions
>>hsa-miR-7113-5p X:7514882|7516290 2511.00     -408.94     162.00      -
28.37 2483  21    1409   70 445 534 1150 474 684 714 746 1255 951 840 1284
624 776 1196 1314
Complete
```

Supplementary Figures

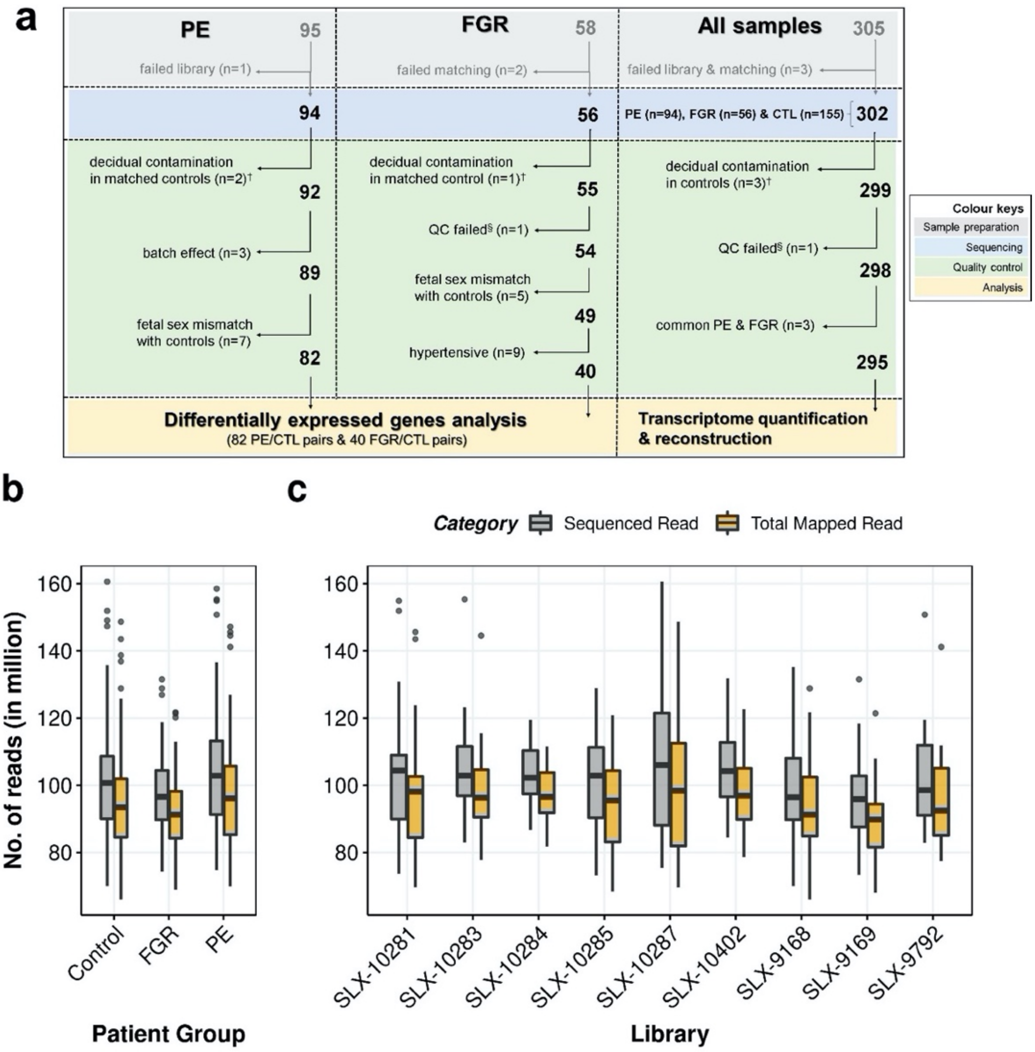

Supplementary Figure 1. Number of placental biopsies and sequencing reads used in the analyses. The number of placental biopsies is shown in (a) and the number of sequencing reads is shown by patient group in (b) and by sequencing library (i.e. batch) in (c). We sequenced both rRNA-depleted long RNAs and small RNA from 302 unique placenta biopsies, including 94 preeclampsia (PE), 56 fetal growth restriction (FGR) and 155 control samples (colored with a blue background in (a)). Note that three placental biopsies were classified as both PE and FGR. After assessing the sequencing quality, we used 244 samples for the differential gene expression analysis (82 case/control pairs in the PE cohort and 40 case/control pairs in the FGR cohort) and 295 samples for transcriptome quantification and reconstruction (91 PE samples, 52 FGR samples, and 152 controls). Note that three samples flagged as 'decidual contamination' (marked †) were control samples (i.e. healthy placenta) and they were excluded from the transcriptome quantification and reconstruction analyses, and the corresponding case-control pairs were excluded in differential gene expression analysis. One sample was flagged as 'QC failed' (marked §) as it showed low mapping ratio (~50%) in GRCh37. The 295 samples consist of 91 PE samples (3 common between PE and FGR removed from 94), 52 FGR samples (1 QC failed and 3 common between PE and FGR removed from 56) and 152 control samples (3 decidual contamination samples removed from 155). Supplementary Data 2 and 3 show sequencing statistics on total and small RNA-Seq datasets, respectively. In panels (b) and (c), the boxes show the median and the lower and upper quartiles. The whiskers extend from the lowest observed point still within 1.5 times IQR (the interquartile range) of the lower quartile, to the highest observed point still within 1.5 times IQR of the upper quartile. All points beyond the whiskers are plotted as outliers.

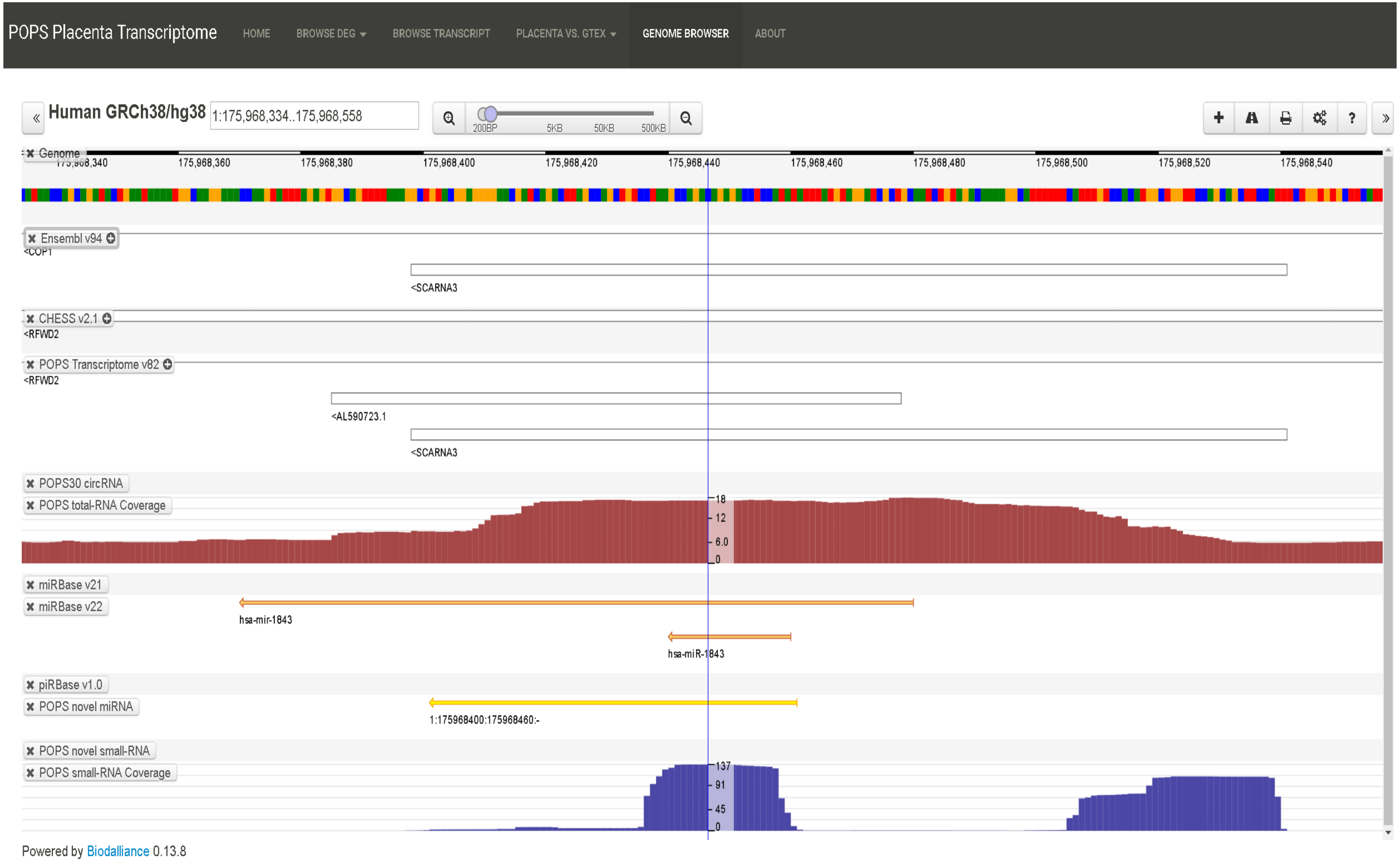
POPS Placenta Transcriptome
HOME
BROWSE DEG
BROWSE TRANSCRIPT
PLACENTA VS. GTEX
GENOME BROWSER
ABOUT
Human GRCh38/hg38
1:175,968,334..175,968,558
200BP
5KB
50KB
500KB
Genome
175,968,340
175,968,360
175,968,380
175,968,400
175,968,420
175,968,440
175,968,460
175,968,480
175,968,500
175,968,520
175,968,540
Ensembl v94
<COP1
<SCARNA3
CHESS v2.1
<RFWD2
POPS Transcriptome v82
<RFWD2
<AL590723.1
<SCARNA3
POPS30 circRNA
POPS total-RNA Coverage
18
12
6.0
0
miRBase v21
miRBase v22
hsa-mir-1843
hsa-miR-1843
piRBase v1.0
POPS novel miRNA
1:175968400:175968460:-
POPS novel small-RNA
POPS small-RNA Coverage
137
91
45
0
Powered by Biodalliance 0.13.8

Supplementary Figure 2. A screenshot from the POPS (Pregnancy Outcome Prediction Study) placenta transcriptome web site.

A genome browser is embedded within our website (https://www.obgyn.cam.ac.uk/placentome). This screenshot illustrates a novel placental miRNA and reconstructed placental transcripts together with publicly available transcript annotations such as Ensembl, CHESS and miRBase. There are 12 tracks as they appear from top to bottom: 1) Genome: genomic coordinates based on GRCh38 version of human genome reference, 2) Ensembl v94: gene and transcript annotations based on Ensembl v94, 3) CHESS v2.1: gene and transcript annotations based on CHESS v2.1, 4) POPS Transcriptome v82: reconstructed placenta transcriptome reported in this study, 5) POPS30 circRNA: placental circRNAs reported in this study, 6) POPS total-RNA coverage: total RNA-Seq data coverage from current study, 7) miRBase v21: micro-RNA annotation from miRBase v21, 8) miRBase v22: micro-RNA transcript annotation from miRBase v22, 9) piRBase v1: piwi-RNA annotation from piRBase v1, 10) POPS novel miRNA: novel placental miRNA reported in this study, 11) POPS novel small-RNA: novel small RNA reported in this study, and 12) POPS small-RNA Coverage: small RNA-Seq data coverage from current study. We report a novel placental miRNA (1:175968401-175968460:-) which was predicted by mirDeep2 using miRBase v21 annotation at the time of this study. More recent version of miRBase (v22) now includes a miRNA (has-mir-1843) where we predict as a miRNA. This genome browser is implemented by using Biodalliance (v0.13.8)[10].

a

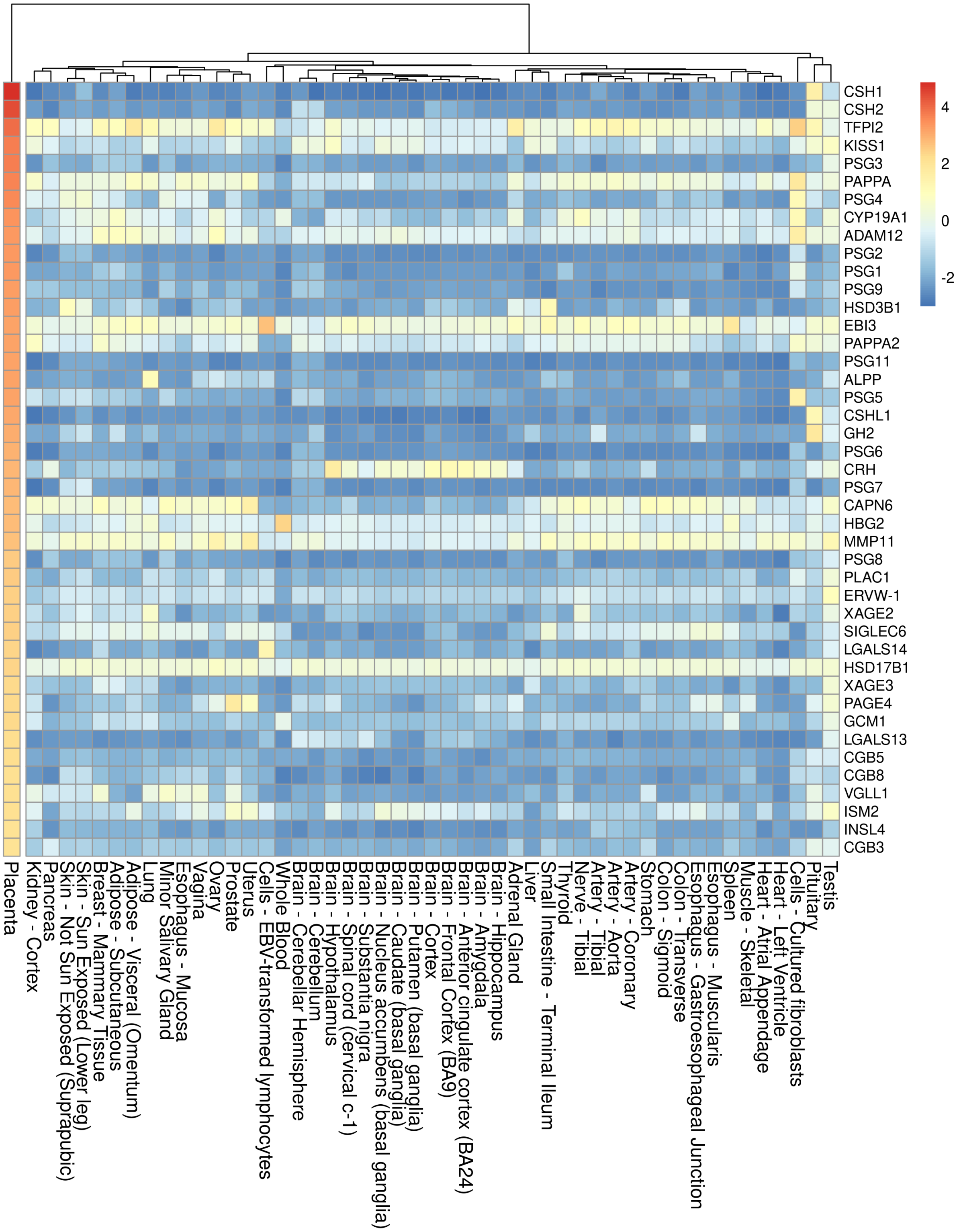
CSH1
CSH2
TFPI2
KISS1
PSG3
PAPPA
PSG4
CYP19A1
ADAM12
PSG2
PSG1
PSG9
HSD3B1
EBI3
PAPPA2
PSG11
ALPP
PSG5
CSHL1
GH2
PSG6
CRH
PSG7
CAPN6
HBG2
MMP11
PSG8
PLAC1
ERVW-1
XAGE2
SIGLEC6
LGALS14
HSD17B1
XAGE3
PAGE4
GCM1
LGALS13
CGB5
CGB8
VGLL1
ISM2
INSL4
CGB3
4
2
0
-2
Placenta
Kidney - Cortex
Pancreas
Skin - Sun Exposed (Lower leg)
Skin - Not Sun Exposed (Suprapubic)
Breast - Mammary Tissue
Adipose - Subcutaneous
Adipose - Visceral (Omentum)
Lung
Minor Salivary Gland
Esophagus - Mucosa
Vagina
Ovary
Prostate
Uterus
Cells - EBV-transformed lymphocytes
Whole Blood
Brain - Cerebellar Hemisphere
Brain - Cerebellum
Brain - Hypothalamus
Brain - Spinal cord (cervical c-1)
Brain - Substantia nigra
Brain - Nucleus accumbens (basal ganglia)
Brain - Caudate (basal ganglia)
Brain - Putamen (basal ganglia)
Brain - Cortex
Brain - Frontal Cortex (BA9)
Brain - Anterior cingulate cortex (BA24)
Brain - Amygdala
Brain - Hippocampus
Adrenal Gland
Liver
Small Intestine - Terminal Ileum
Thyroid
Nerve - Tibial
Artery - Tibial
Artery - Aorta
Artery - Coronary
Stomach
Colon - Sigmoid
Colon - Transverse
Esophagus - Gastroesophageal Junction
Esophagus - Muscularis
Spleen
Muscle - Skeletal
Heart - Atrial Appendage
Heart - Left Ventricle
Cells - Cultured fibroblasts
Pituitary
Testis

b

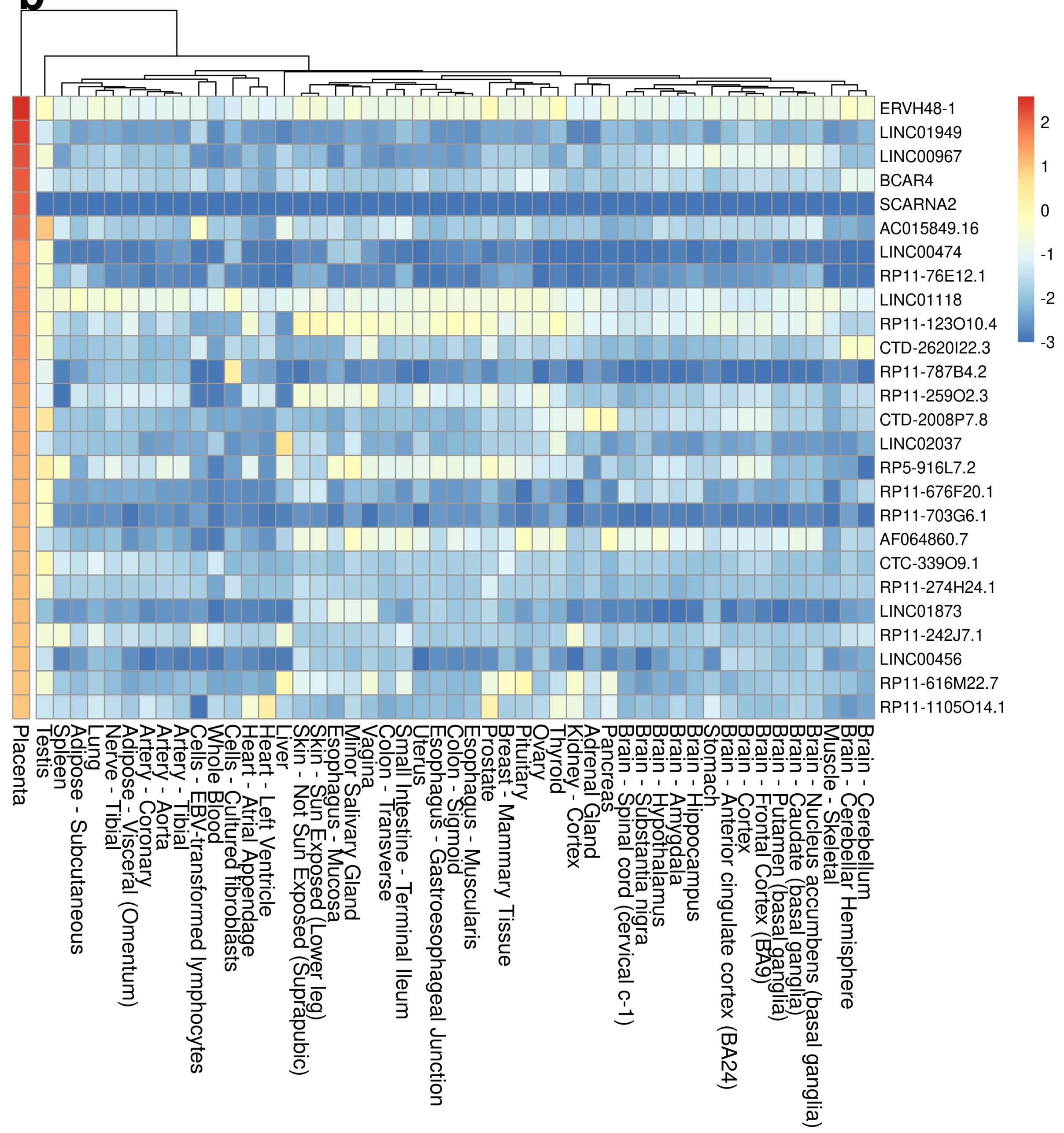
ERVH48-1
LINC01949
LINC00967
BCAR4
SCARNA2
AC015849.16
LINC00474
RP11-76E12.1
LINC01118
RP11-123O10.4
CTD-2620I22.3
RP11-787B4.2
RP11-259O2.3
CTD-2008P7.8
LINC02037
RP5-916L7.2
RP11-676F20.1
RP11-703G6.1
AF064860.7
CTC-339O9.1
RP11-274H24.1
LINC01873
RP11-242J7.1
LINC00456
RP11-616M22.7
RP11-1105O14.1
2
1
0
-1
-2
-3
Placenta
Testis
Spleen
Adipose - Subcutaneous
Lung
Nerve - Tibial
Adipose - Visceral (Omentum)
Artery - Coronary
Artery - Aorta
Artery - Tibial
Cells - EBV-transformed lymphocytes
Whole Blood
Cells - Cultured fibroblasts
Heart - Atrial Appendage
Heart - Left Ventricle
Liver
Skin - Not Sun Exposed (Suprapubic)
Skin - Sun Exposed (Lower leg)
Esophagus - Mucosa
Minor Salivary Gland
Vagina
Colon - Transverse
Small Intestine - Terminal Ileum
Uterus
Esophagus - Gastroesophageal Junction
Colon - Sigmoid
Esophagus - Muscularis
Prostate
Breast - Mammary Tissue
Pituitary
Ovary
Thyroid
Kidney - Cortex
Adrenal Gland
Pancreas
Brain - Spinal cord (cervical c-1)
Brain - Substantia nigra
Brain - Hypothalamus
Brain - Amygdala
Brain - Hippocampus
Stomach
Brain - Anterior cingulate cortex (BA24)
Brain - Cortex
Brain - Frontal Cortex (BA9)
Brain - Putamen (basal ganglia)
Brain - Caudate (basal ganglia)
Brain - Nucleus accumbens (basal ganglia)
Muscle - Skeletal
Brain - Cerebellar Hemisphere
Brain - Cerebellum

c

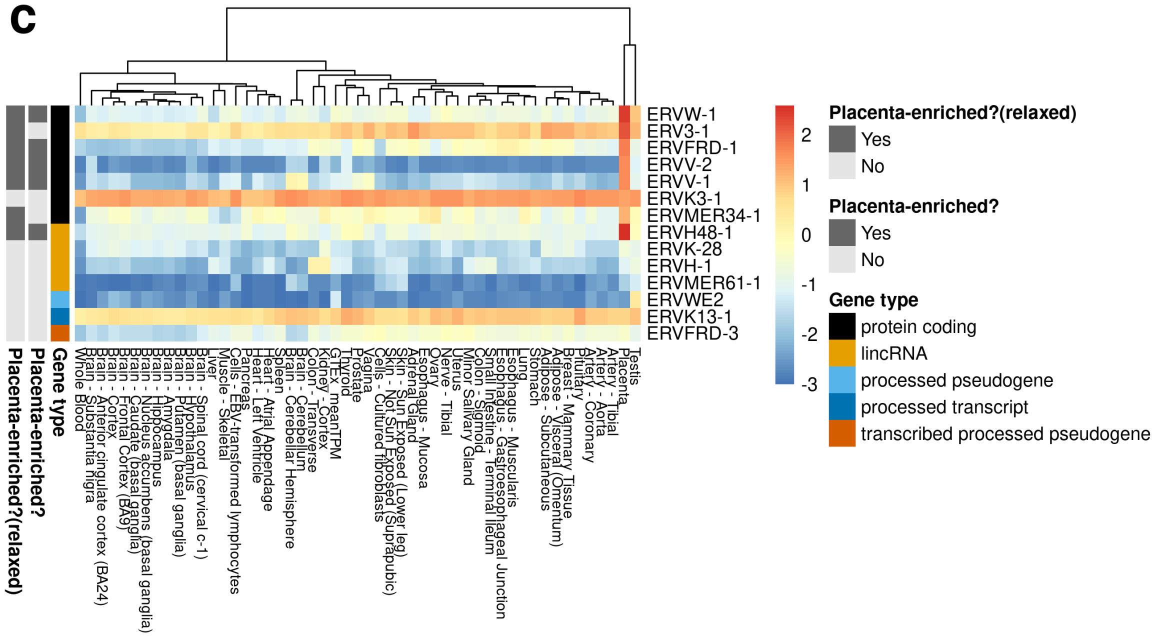

Supplementary Figure 3. Heatmaps showing the abundance level of genes enriched in the placenta and endogenous retrovirus genes

Each column represents a tissue and each row represents a placenta-enriched: (**a**) protein coding gene, (**b**) long non-coding gene and (**c**) an endogenous retrovirus genes. The columns (tissues) are hierarchically clustered by the similarity of the expression level of genes (row) which are ordered by their abundance level in the placenta. Of the 71 and 74 placenta-enriched protein coding and long non-coding genes (Supplementary Data 5 and 6), 43 and 26 genes, respectively, are displayed in (**a**) and (**b**). These were selected on the basis of their abundance (≥100 Transcript Per Million (TPM) for the protein coding genes and ≥10 TPM for the long non-coding genes). The TPMs of 14 ERV genes are shown in Supplementary Data 7. Note that non-polyadenylated transcripts were not considered in tissue-wide comparison. The color scale represents the relative abundance calculated as following: ($\log_{10}$(TPM+0.001)). These heatmaps are also available from https://www.obgyn.cam.ac.uk/placentome.

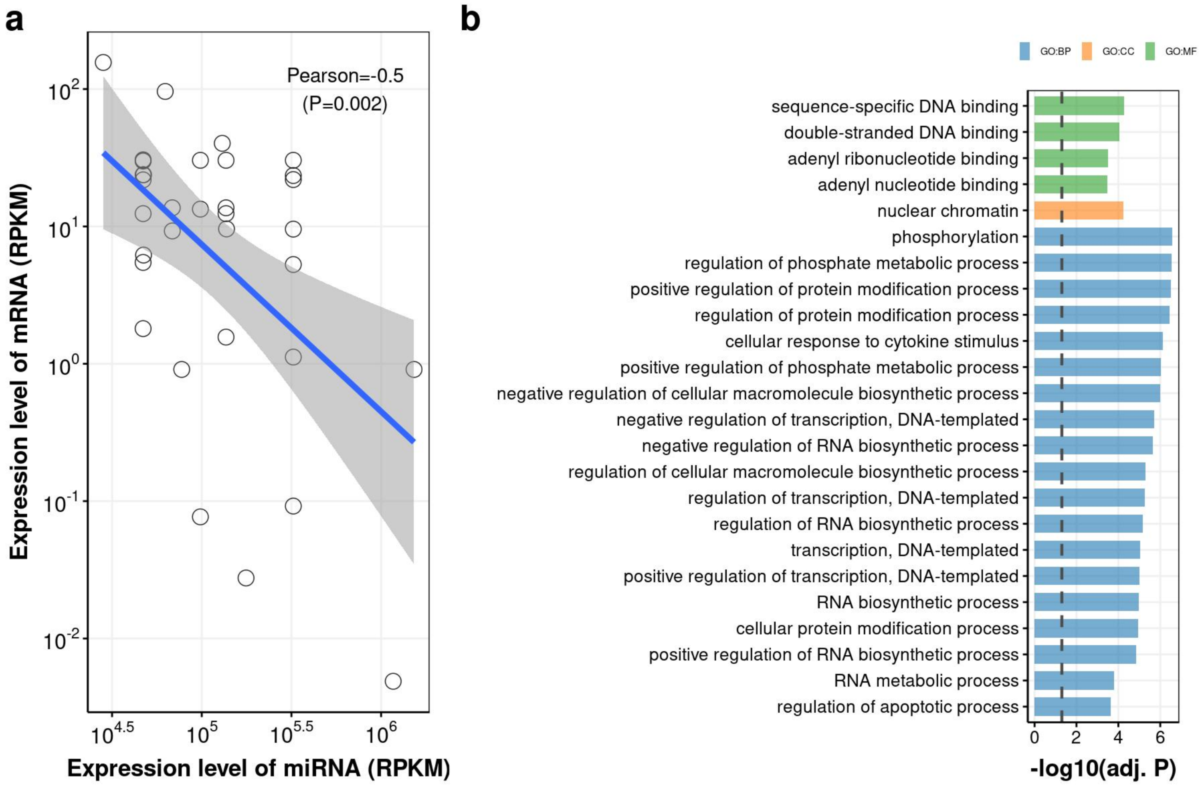

Supplementary Figure 4. Highly expressed miRNAs from C19MC and their binding targets

a) The correlation coefficient between the expression level of miRNAs (x-axis) and their binding targets (y-axis) is shown. Each dot represents the mean expression level across quantified samples. A fitted linear regression line is shown in blue with 95% confidence interval in grey. **b**) Significantly overrepresented Gene Ontology terms are shown for the binding targets. 33 highly expressed miRNAs in C19MC (i.e. top 5%) were investigated where a total of 14 miRNAs were reported to bind 27 target mRNAs based on miRTarBase (see Methods for details).

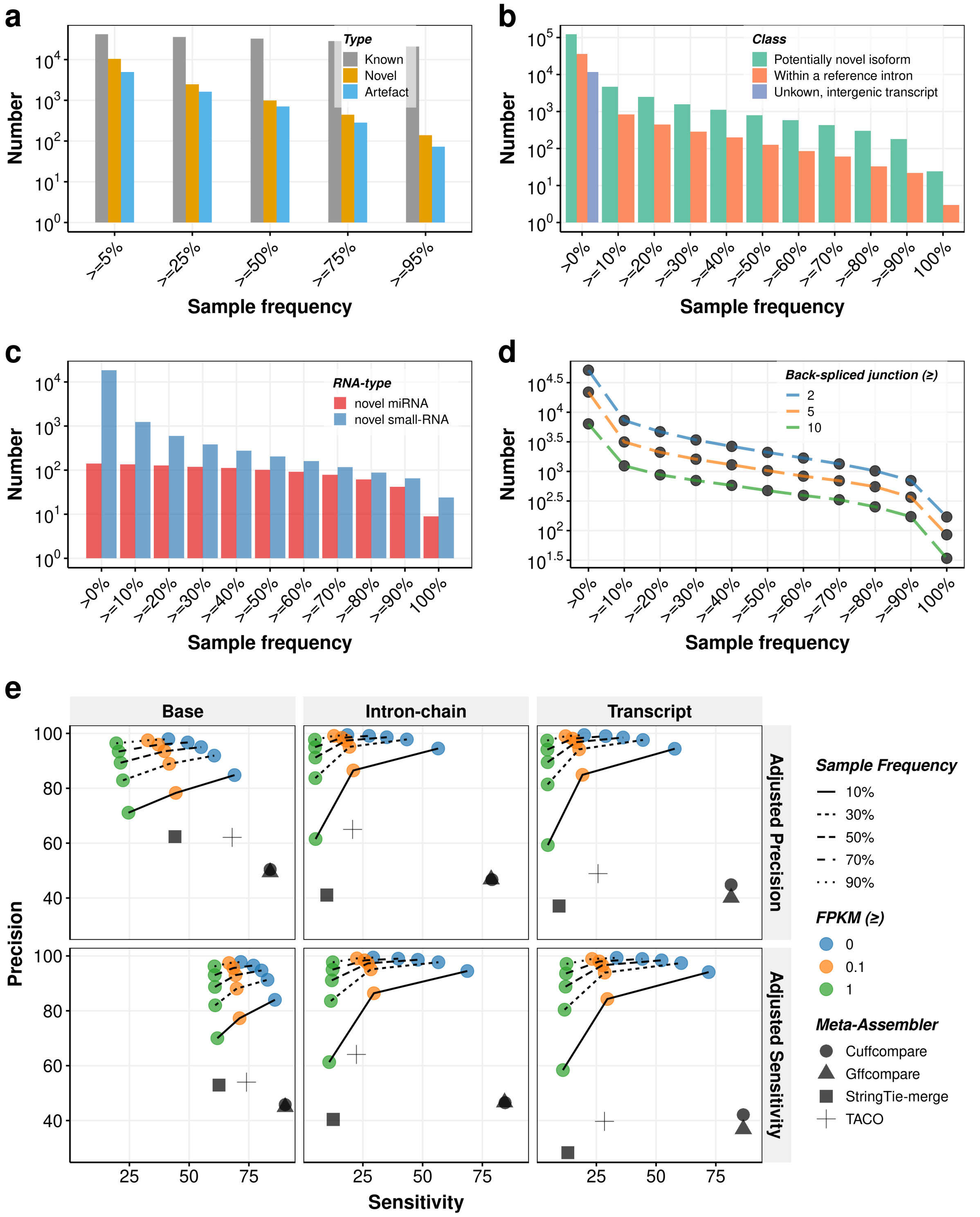

Supplementary Figure 5. Numbers of placental transcripts and assessment of reconstructed transcriptome

The numbers of placental transcripts are shown as following: (**a**) transcripts detected at least 0.1 RPKM are shown for the re-constructed placental transcriptome; (**b**) novel transcripts; (**c**) novel miRNA and novel small-RNA, and (**d**) circular RNAs. The number of transcripts in (**a**) and (**b**) are calculated from the 295 total RNA-Seq placental datasets using StringTie[11]. In (**a**), 'Artefact' is defined as one of the following classification codes from *cuffcompare*: 1) multiple classifications (code: .), 2) possible polymerase run-on (code: p), 3) possible pre-mRNA fragment, 4) intron on the opposite strand (code: s), and 5) repeat (code: r). The numbers of

novel miRNA and novel small-RNAs (**c**) are from the small RNA-Seq datasets. The circular RNAs (**d**) are predicted using CIRI2[12] using the 295 total RNA-Seq data sets. There is a total of 51,384 circRNAs present in at least one placental sample, but the number drops to 3,399, 700 and 170 at 30% (POPS30), 90% (POPS90), and 100% of the placental samples, respectively, after applying a sample frequency threshold. In (**e**), we compared our re-constructed placental transcriptome, assembled by Cuffcompare, with three alternative approaches that can also assemble multi-sample transcriptome (i.e. meta-assembler): Gffcompare, StringTie-merge and TACO. For benchmarking, we used the same input GTF files (n=295) which were initially assembled by StringTie. The initial Cuffcompare results (our approach) are shown as black circles and we applied various minimum transcript abundance (RPKM) thresholds (represented as blue, green and red circles) and sample frequencies (represented as solid and dotted lines). The assessment of alternative approaches using Gffcompare, StringTie-merge and TACO are shown as black triangles, black squares and grey crosses, respectively. Two benchmark measurements, precision (y-axis) and sensitivity (x-axis), were obtained by running Gffcompare with Ensembl as a reference transcript definition and adjusted as following: 1) ignoring input transcripts that are not overlapped by any transcript in the reference (shown as 'Adjusted Precision') and 2) ignoring reference transcripts that are not overlapped by any input transcript (shown as 'Adjusted Sensitivity'). Sensitivity and precision values were estimated at base, intron-chain, and transcript level, as defined from Gffcompare manual (https://ccb.jhu.edu/software/stringtie/gffcompare.shtml).

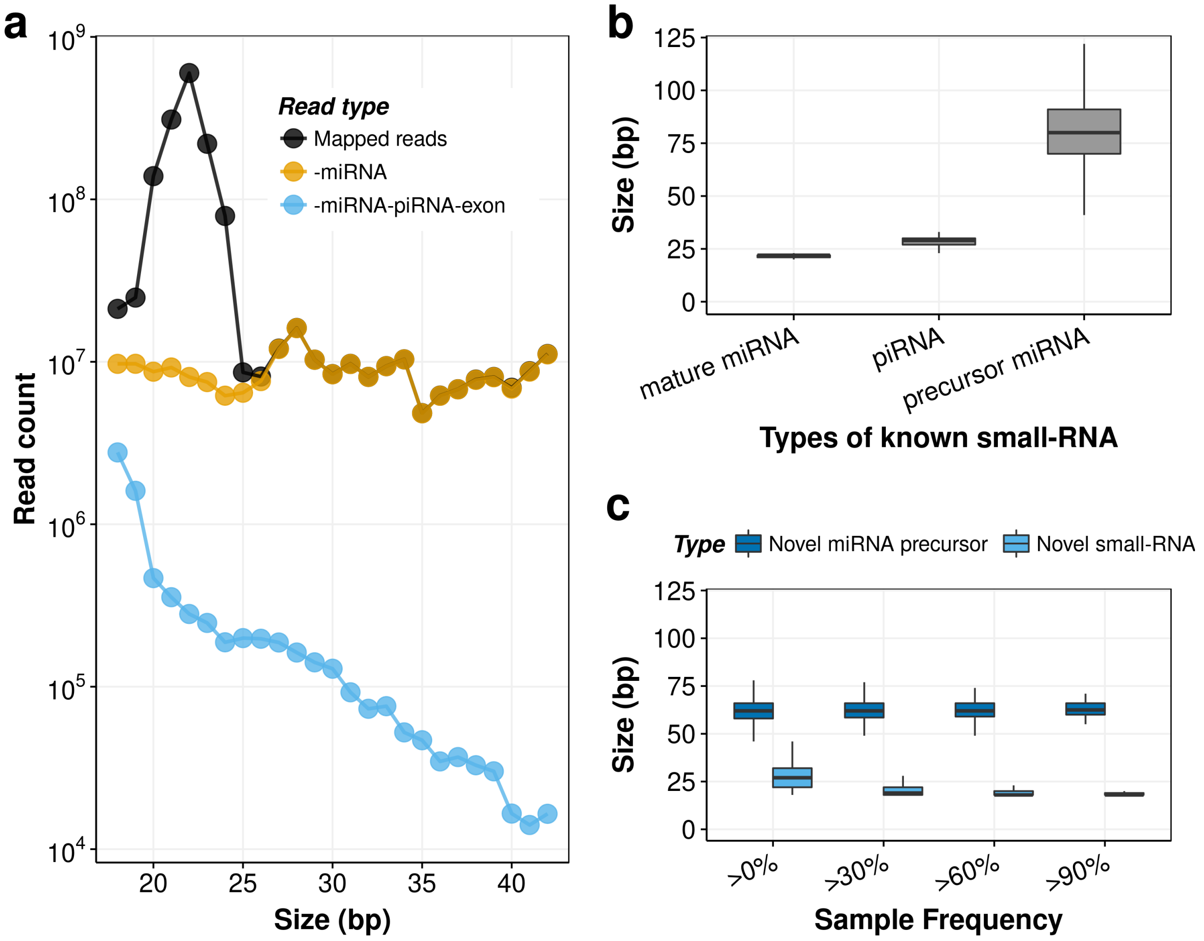

Supplementary Figure 6. Size distribution of placental small RNAs

a) The size distributions of short reads mapped to GRCh38 are shown by their mapping types defined as followings: 1) 'Mapped reads': reads of at least 10x coverage, 2) '-miRNA': 'Mapped reads' with reads overlapping with any mature miRNA region removed, 3) '-miRNA-piRNA-exon': 'Mapped reads' with reads overlapping either mature miRNA regions*, piRNAs* or exonic regions (see Supplemental Methods for details) removed. **b**) The size distributions of both mature (n=2,588) and precursor miRNAs (n=1,881; based on *miRBase*) and piRNAs (n=32,235; based on *piRbase*) are shown as box-and-whisker plots and their median sizes are as follow: 22 (mature miRNA), 29 (piRNA) and 80 (precursor miRNA). **c**) The size distributions of novel precursor miRNAs and novel small-RNAs are shown as box-and-wisher plots by their minimum sample frequencies on the x-axis. The median sizes are as follow: 27 (novel small-RNA) and 62 (novel miRNA) at >0%; 19 (novel small-RNA) and 62 (novel miRNA) at >30%; 18 (novel small-RNA) and 62 (novel miRNA) at >60%; 18 (novel small-RNA) and 63 (novel miRNA) at >90%. Novel miRNA and novel small-RNAs are shown in Supplementary Data 15 and 16, respectively. In panels (b) and (c), the boxes show the median and the lower and upper quartiles. The whiskers extend from the lowest observed point still within 1.5 times IQR (the interquartile range) of the lower quartile, to the highest observed point still within 1.5 times IQR of the upper quartile.

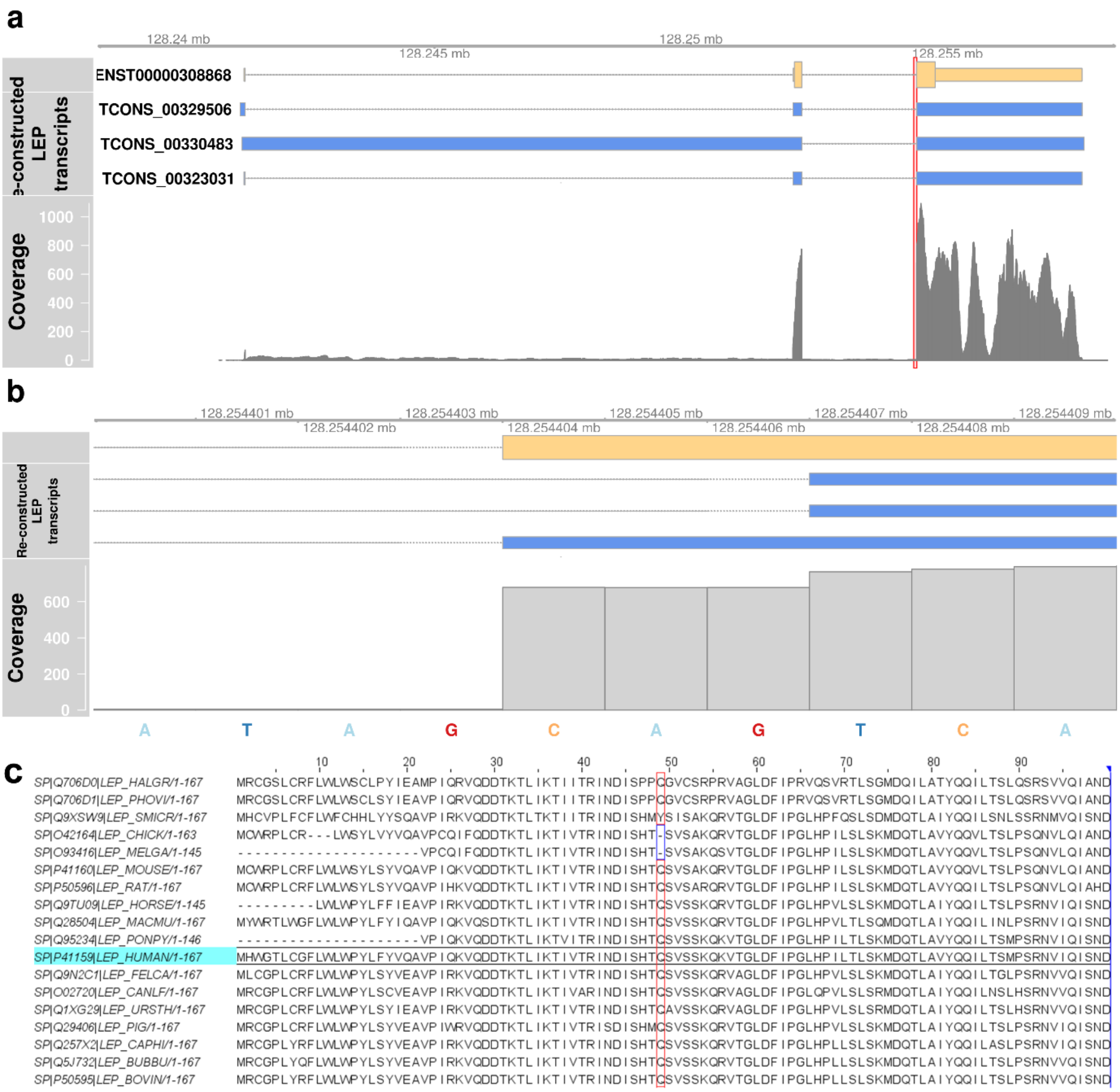

d

	Patient groups		Re-constructed LEP transcripts		
			TCONS_00329506	TCONS_00320483	TCONS_00323031
Number of sample	Control	152	16	13	131
	FGR	52	2	2	51
	PE	91	23	16	86
Total number of sample		295	41	31	268
P-value (Chi-squared test)			0.0011	0.0289	0.6713

Supplementary Figure 7. Three re-constructed LEP transcripts

The reference *LEP* transcript (ENST00000308868) and the three re-constructed transcripts (TCONS_00329506, TCONS_00320483 and TCONS_00323031) present in at least 10% of the cohort are shown (**a**) across the entire LEP locus; (**b**) the first 6 bases at the start region of the last exon. In **a**, the vertical red rectangle indicates the locus shown in **b**. The reference and re-constructed transcripts are colored in yellow and blue, respectively, with their exon regions in rectangular shapes linked by the introns indicated by the horizontal lines. Within the exonic regions of the reference transcript (yellow rectangles), the coding regions are represented as thicker rectangles than the UTRs. The normalized RNA-Seq coverage is shown as a gray colored histogram at the bottom of each panel with their scale ranging from 0 to 1093. The chromosome coordinates (GRCh38) are shown as a scale at the top of each

panel. Of the three re-constructed transcripts, two transcripts are potentially novel isoforms (TCONS_00329506 and TCONS_00320483) and the third (TCONS_00323031) corresponds to the reference transcript ENST00000308868. The order of transcripts in **b** is the same as in **a**. Multiple sequence alignment of LEP protein sequences is shown in **c**. The LEP protein sequences were obtained from 18 species available in UniProt where their sequences were flagged as 'reviewed' (i.e. available in SwissProt). The multiple sequence alignments were generated running CLUSTAL O (v.1.2.4) from UniProt web site. The species are shown by their taxonomical order. The human LEP is highlighted in cyan and the 49th alignment position is the first amino acid (Q) of the last exon, which is colored in a red box except LEP_CHICK (chicken) and LEP_MELGA (wild turkey) which do not have glutamine at the position (colored in blue box). Note that only the first 100 alignment positions are shown. In **d**, the number of samples supporting three re-constructed transcripts are shown by the patient groups (i.e. PE, FGR and Control). We calculated *P* value of observing the numbers of samples supporting a re-constructed transcript, given 295 samples used for transcriptome re-construction. For example, TCONS_00329506 was observed in 16 controls, 2 FGR and 23 PE samples and this is significantly different (P value=0.0011; chi-squared test) given the samples used (152 controls, 52 FGR and 91 PE).

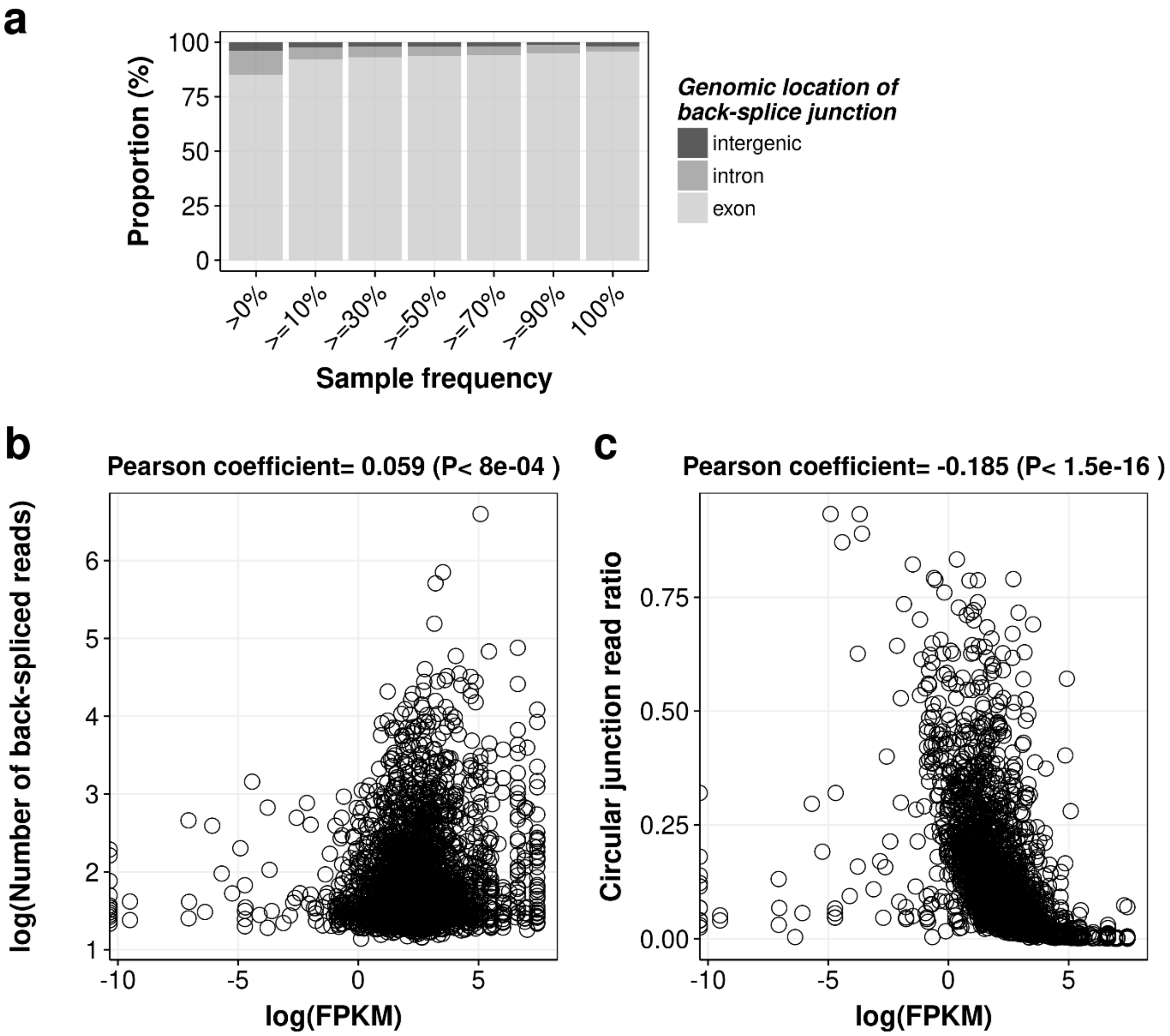

Supplementary Figure 8. Genomic locations of back-splice sites and correlation between the abundance level of hosting genes and the abundance of circRNAs

In (**a**) the genomic locations of back-splice junction sites are shown as proportions of intergenic, intron and exonic regions (y-axis) with their frequencies of samples present with circRNAs (x-axis) from any one sample (i.e >0%) to all 295 placenta samples (i.e. 100%). The expression level (RPKM) of hosting genes is plotted with the number of back-spliced reads (**b**) and the ratio of circular junction read (**c**), as proxies of the amount of circRNAs. The ratio of circular junction reads calculated by following formula: 2x(number of junction reads)/(2x(number of junction reads) + (number of non-junction read)). We used 3,279 circular RNAs for this plot and they were present in at least 30% of the rRNA depleted datasets and absent from the polyA+ datasets. The Pearson correlation coefficient was calculated with the original RPKM value (i.e. non-logarithm) using ‘*cor.test*’ function of R ‘*stats*’ package with default options (two-sided test).

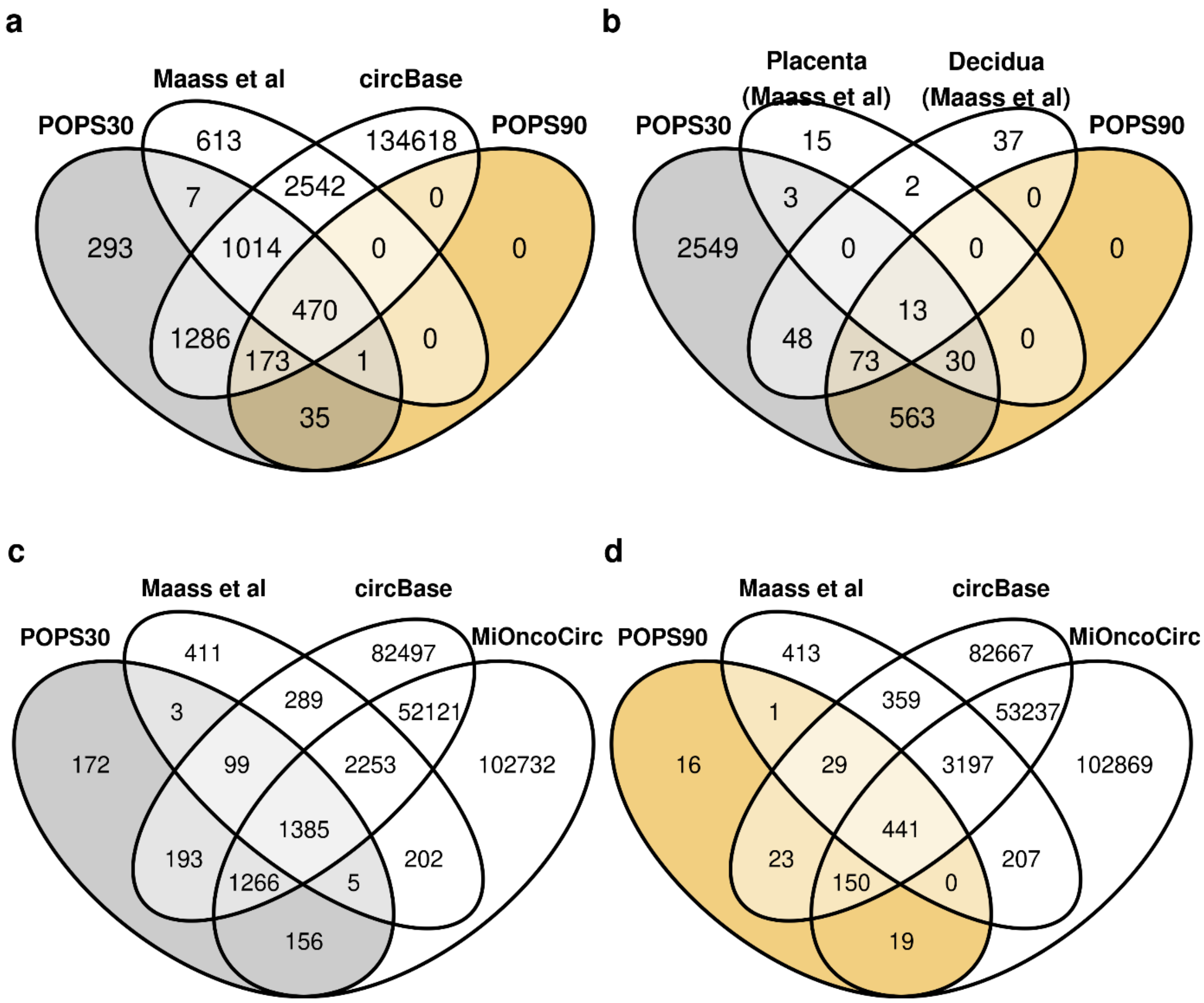

Supplementary Figure 9. Venn diagrams showing the number of circRNAs in our study and others.
In our study, 3,279 circRNAs present in 30% of cohort (POPS30) and 679 circRNAs present in 90% of cohort (POPS90) were compared with those reported by Maass *et al.*[13] and circBase[14] (**a**), and circRNAs in the placenta and decidua tissues reported by Maass *et al.* (**b**). POPS30 and POPS90 were compared with Maass *et al.*, circBase and MiOncoCirc[15] in (**c**) and (**d**), respectively.

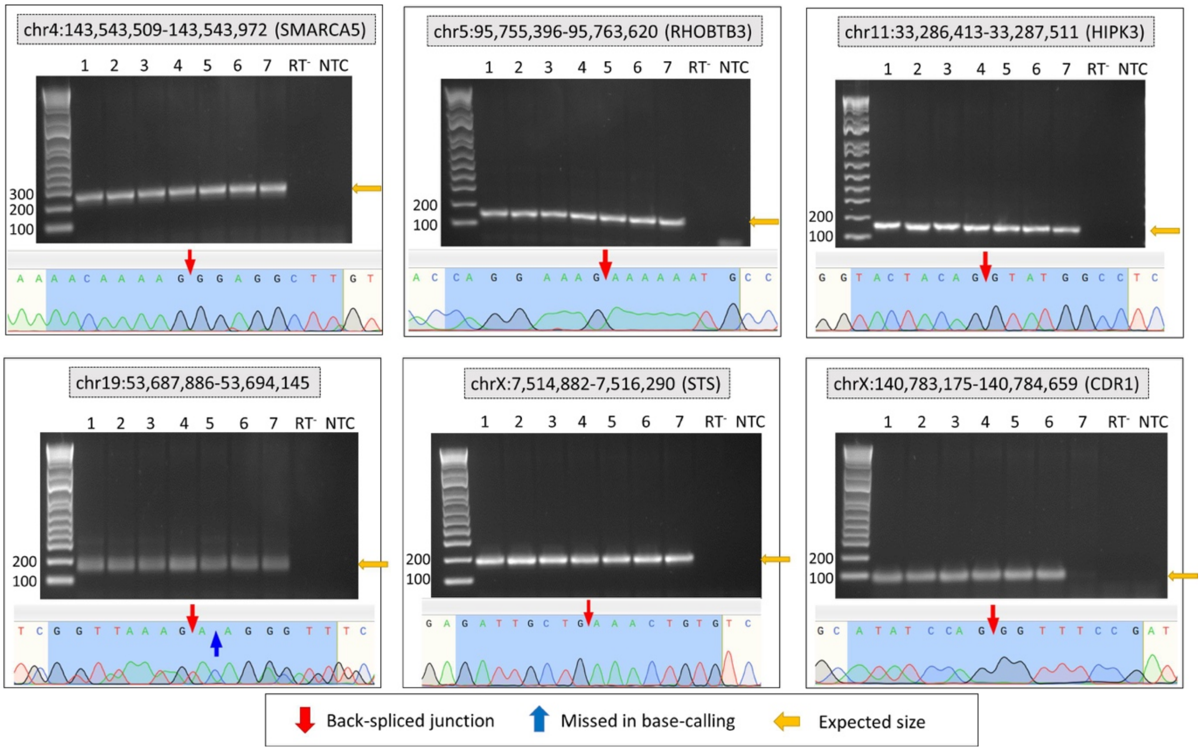

Supplementary Figure 10. Validation of predicted circRNAs.

The results of 6 validation assays are shown. Seven placenta samples were tested, and from the bioinformatic analysis described earlier, sample-7 was predicted not to be positive for circ*STS* and *CDR1as*. The expected size of each back-spliced PCR product is shown in yellow (also in Supplementary Data 21) and the size markers are shown in base pairs. The red arrows indicate the positions of predicted back-spliced junctions which were validated by Sanger sequencing (bottom of each panel). RT$^-$: no reverse transcriptase; NTC: no template control.

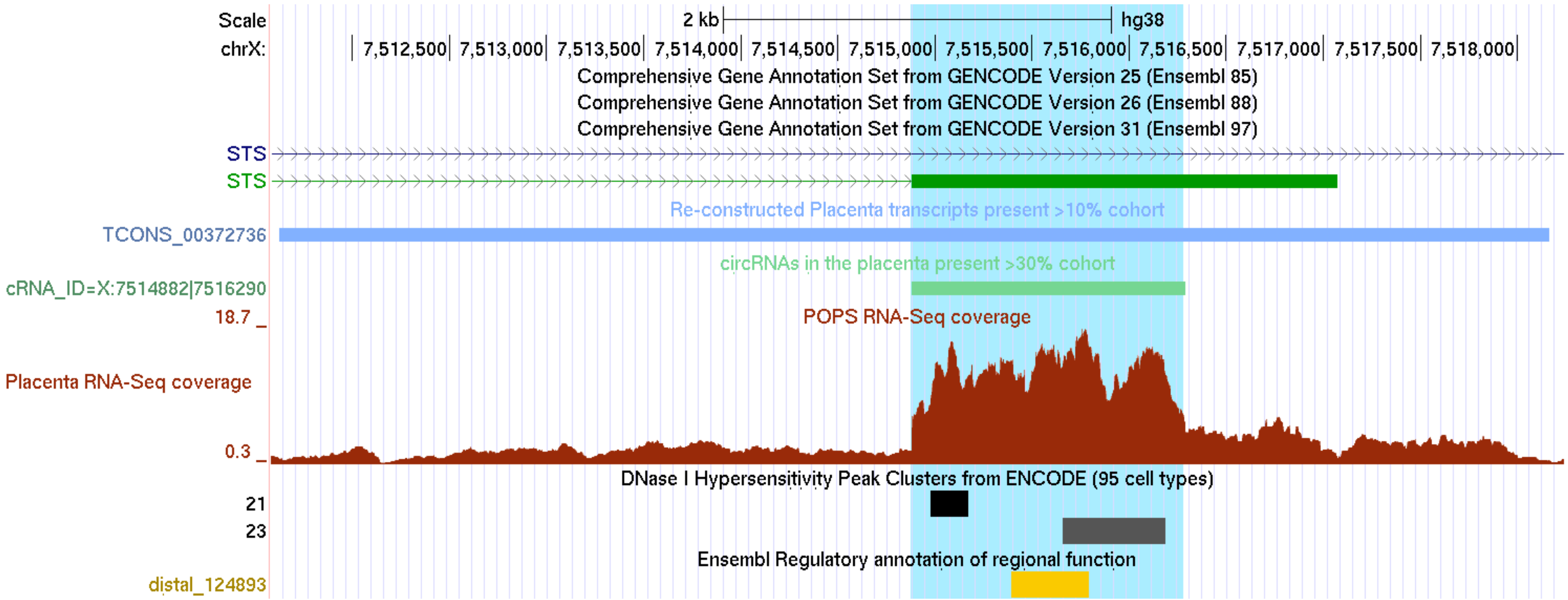

Supplementary Figure 11. Genome browser views of circSTS

The genomic location of circSTS (chrX:7,514,882-7,516,290) is shown from the UCSC genome browser. The circRNAs chrX:7,514,882-7,516,290 (X:7514882|7516290, colored in light green) was annotated as being within an intergenic region at the time of this study (based on Ensembl 90). However, using on the latest gene annotation (Ensembl 97) this circRNA shares the same exonic start position (chrX:7,514,882) as a lincRNA (colored in dark green), within the STS gene (steroid sulfatase). Our reconstructed placental transcriptome data suggested a novel single exon transcript (panel colored in light blue) present at modest abundance (1.02 RPKM, in 16% of placental samples). The Ensembl regulatory build annotated the region as an intergenic enhancer in Ensembl (colored in orange). The peaks in the RNA-Seq coverage graph (colored in maroon) correspond to the location of back-spice junction. In-house genome browser is available from https://www.obgyn.cam.ac.uk/placentome.

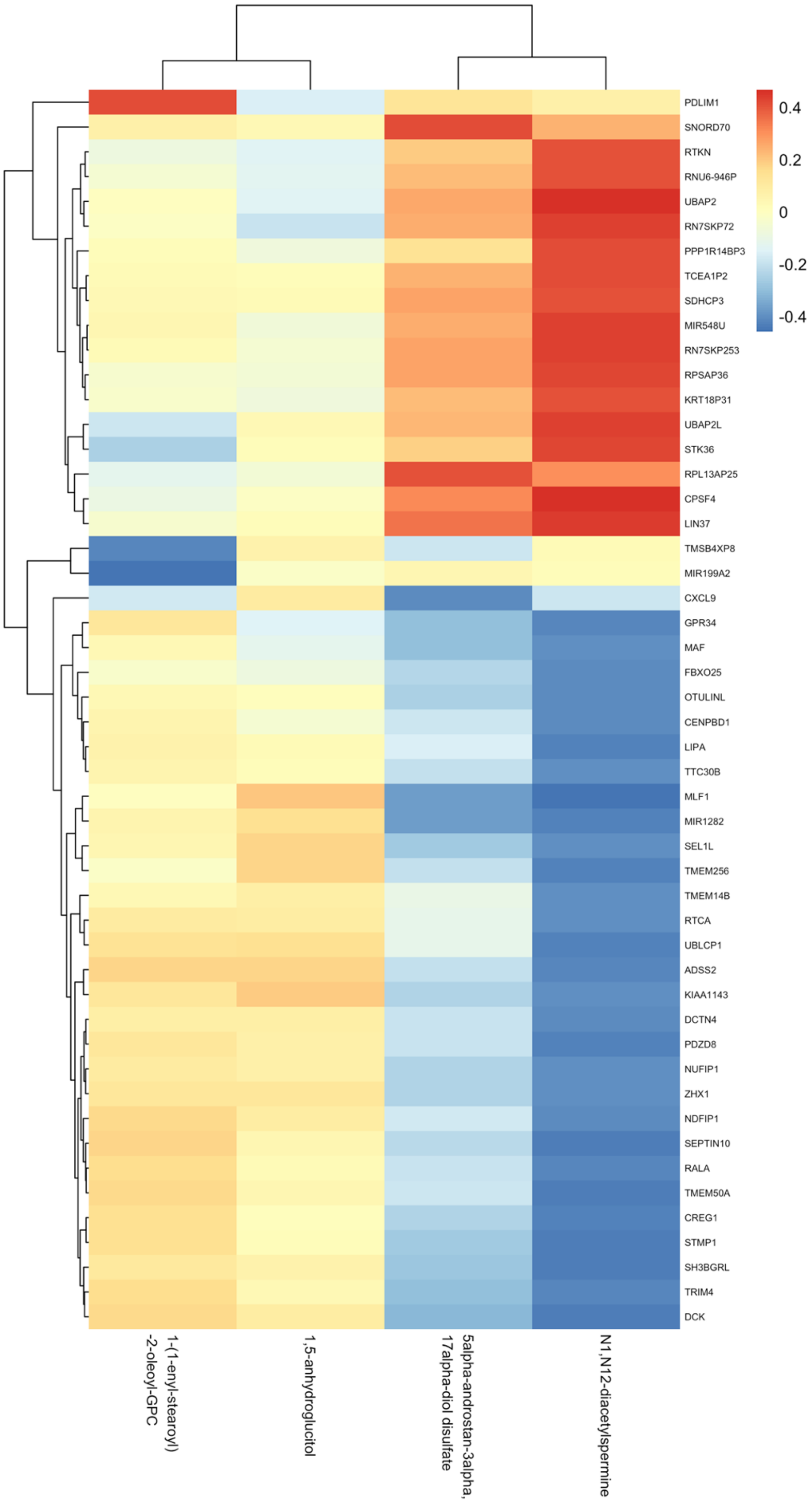

Supplementary Figure 12. Heatmap showing clustering of the correlations between 4 circulating metabolites and placental mRNAs

Correlations between the four previously identified metabolites that correlate with fetal size and placental transcripts were calculated. Details of the correlations and adjusted *P* values are provided in Supplementary Data 23.

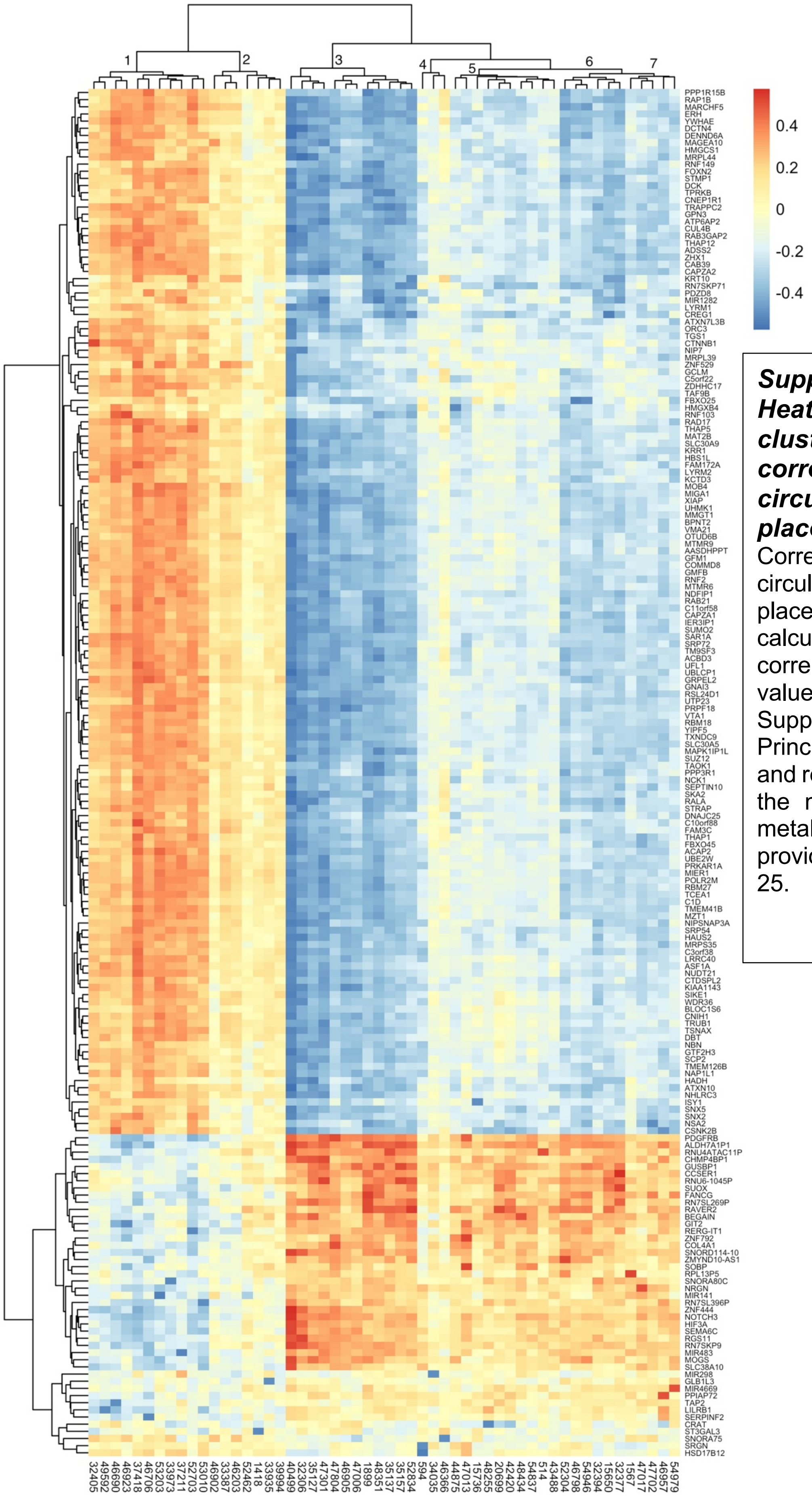

Supplementary Figure 13. Heatmap showing clustering of the correlations between circulating metabolites and placental mRNAs
Correlations between the circulating metabolites and placental transcripts were calculated. Details of the correlations and adjusted P values are provided in Supplementary Data 24. Principle clusters are labeled and referred to in the main text of the manuscript. Details of the metabolites (columns) are provided in Supplementary Data 25.

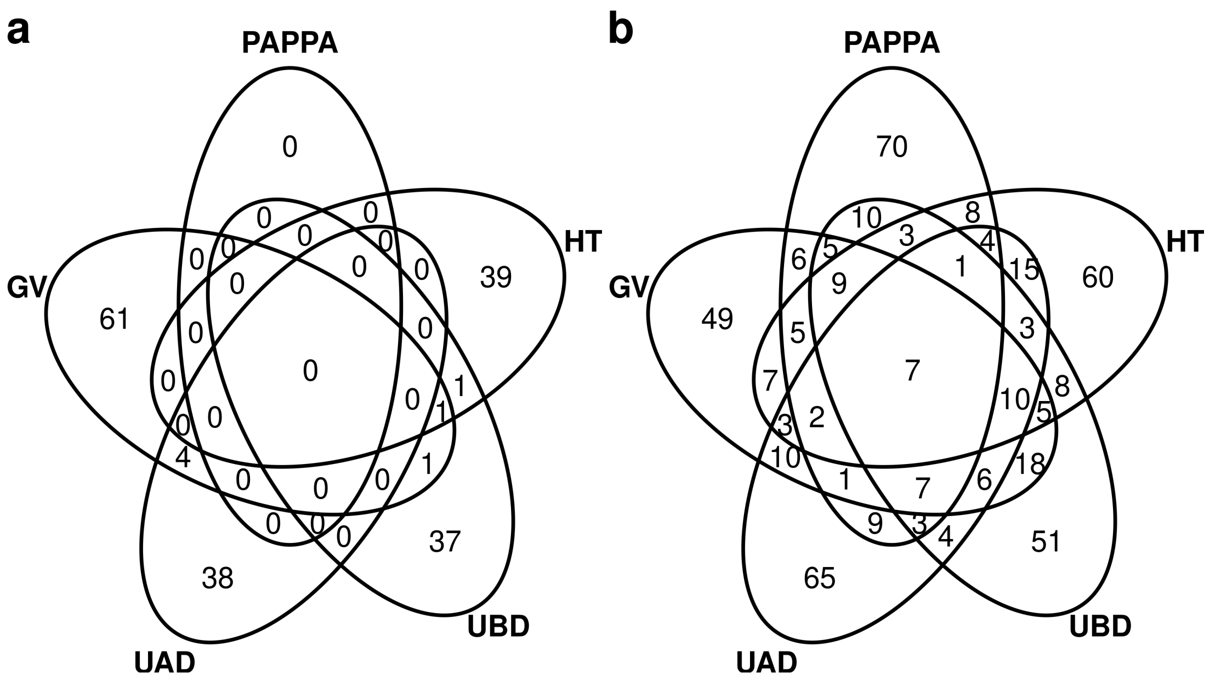

Supplementary Figure 14. Venn diagrams showing the number of differentially regulated transcripts in five FGR sub-categories.

The numbers of transcripts are shown based on the *P* value approach in (**a**) and the fold-change approach in (**b**). Two-sided *P* values were adjusted (Benjamini-Hochberg correction method with FDR ≤5%) based on the original *P* values reported from DESeq2. For the fold-change approach, 150 top ranked transcripts (i.e. top 3%) were selected per each FGR sub-category. HT (n=12): FGR infant whose mother experienced any hypertensive disorder. PAPPA (n=10): FGR infant from the normotensive mother who showed abnormal PAPP-A level. GV (n=11): FGR infant showing abnormal fetal growth velocity from the normotensive mother. UAD (n=6): FGR infant from the normotensive mother who showed abnormal uterine blood flow. UBD (n=9): FGR infant from the normotensive mother who showed abnormal umbilical blood flow. In **b**), transcripts from the following seven genes were common shared among the five sub-categories: *ANGPT2, COL17A1, FOS, HTRA4, LEP, PPL,* and *PROCR*.

References

1 Pertea, M. *et al.* CHESS: a new human gene catalog curated from thousands of large-scale RNA sequencing experiments reveals extensive transcriptional noise. *Genome Biol* **19**, 208, doi:10.1186/s13059-018-1590-2 (2018).

2 Pundir, S., Martin, M. J. & O'Donovan, C. UniProt Protein Knowledgebase. *Methods Mol Biol* **1558**, 41-55, doi:10.1007/978-1-4939-6783-4_2 (2017).

3 Zhang, Y. *et al.* Positional cloning of the mouse obese gene and its human homologue. *Nature* **372**, 425-432, doi:10.1038/372425a0 (1994).

4 Niknafs, Y. S., Pandian, B., Iyer, H. K., Chinnaiyan, A. M. & Iyer, M. K. TACO produces robust multisample transcriptome assemblies from RNA-seq. *Nat Methods* **14**, 68-70, doi:10.1038/nmeth.4078 (2017).

5 Trapnell, C. *et al.* Transcript assembly and quantification by RNA-Seq reveals unannotated transcripts and isoform switching during cell differentiation. *Nat Biotechnol* **28**, 511-515, doi:10.1038/nbt.1621 (2010).

6 Liu, F., Dong, H., Mei, Z. & Huang, T. Investigation of miRNA and mRNA Co-expression Network in Ependymoma. *Front Bioeng Biotechnol* **8**, 177, doi:10.3389/fbioe.2020.00177 (2020).

7 Langfelder, P. & Horvath, S. WGCNA: an R package for weighted correlation network analysis. *BMC Bioinformatics* **9**, 559, doi:10.1186/1471-2105-9-559 (2008).

8 Raudvere, U. *et al.* g:Profiler: a web server for functional enrichment analysis and conversions of gene lists (2019 update). *Nucleic Acids Res* **47**, W191-W198, doi:10.1093/nar/gkz369 (2019).

9 Reimand, J., Kull, M., Peterson, H., Hansen, J. & Vilo, J. g:Profiler--a web-based toolset for functional profiling of gene lists from large-scale experiments. *Nucleic Acids Res* **35**, W193-200, doi:10.1093/nar/gkm226 (2007).

10 Down, T. A., Piipari, M. & Hubbard, T. J. Dalliance: interactive genome viewing on the web. *Bioinformatics* **27**, 889-890, doi:10.1093/bioinformatics/btr020 (2011).

11 Pertea, M. *et al.* StringTie enables improved reconstruction of a transcriptome from RNA-seq reads. *Nat Biotechnol* **33**, 290-295, doi:10.1038/nbt.3122 (2015).

12 Gao, Y., Zhang, J. & Zhao, F. Circular RNA identification based on multiple seed matching. *Brief Bioinform*, doi:10.1093/bib/bbx014 (2017).

13 Maass, P. G. *et al.* A map of human circular RNAs in clinically relevant tissues. *J Mol Med (Berl)* **95**, 1179-1189, doi:10.1007/s00109-017-1582-9 (2017).

14 Glazar, P., Papavasileiou, P. & Rajewsky, N. circBase: a database for circular RNAs. *RNA* **20**, 1666-1670, doi:10.1261/rna.043687.113 (2014).

15 Vo, J. N. *et al.* The Landscape of Circular RNA in Cancer. *Cell* **176**, 869-881 e813, doi:10.1016/j.cell.2018.12.021 (2019).
