# Supplementary material for: The RNA landscape of the human placenta in health and disease
Source: Nat Commun. 2021 May 11;12:2639. doi: 10.1038/s41467-021-22695-y (PMC8113443; doi:10.1038/s41467-021-22695-y)
Supplement: Supplementary file 4 — Description of Additional Supplementary Files [file 41467_2021_22695_MOESM4_ESM.docx]

Description of additional supplementary information:

Title: Supplementary Data 1

Description: Previous studies of the human placenta transcriptome

Title: Supplementary Data 2

Description: Mapping statistics for total RNA-Seq experiments

Title: Supplementary Data 3

Description: Mapping statistics for small RNA-Seq experiments

Title: Supplementary Data 4

Description: Distribution of RPKM values by RNA type

Title: Supplementary Data 5

Description: Tissue enriched protein coding genes

Title: Supplementary Data 6

Description: Tissue enriched lincRNAs

Title: Supplementary Data 7

Description: Expression level of endogenous retrovirus genes across tissues

Title: Supplementary Data 8

Description: circRNAs predicted to be present in non oligo-dT libraries

Title: Supplementary Data 9

Description: circRNAs predicted to be present in oligo-dT libraries

Title: Supplementary Data 10

Description: Genes hosting at least 10 circRNAs in the placenta

Title: Supplementary Data 11

Description: Abundance of mature miRNA in the placenta

Title: Supplementary Data 12

Description: Highly expressed C19MC miRNAs and their binding targets

Title: Supplementary Data 13

Description: Abundance of piRNA in the placenta

Title: Supplementary Data 14

Description: Abundance of small non-coding RNA (sncRNA) in the placenta

Title: Supplementary Data 15

Description: Novel miRNA in the placenta

Title: Supplementary Data 16

Description: Novel small RNA in the placenta

Title: Supplementary Data 17

Description: Comparison of novel re-constructed placental transcripts with CHESS database

Title: Supplementary Data 18

Description: Mapped reads aligned at the start position of the third exon of leptin transcript

Title: Supplementary Data 19

Description: Leptin variants in other tissues

Title: Supplementary Data 20

Description: Genes hosting at least 5 unreported circRNAs

Title: Supplementary Data 21

Description: Primers used in RT-qPCR for circRNAs

Title: Supplementary Data 22

Description: Putative peptides from circRNAs

Title: Supplementary Data 23

Description: Correlations among 4 circulating metabolites and placental transcripts

Title: Supplementary Data 24

Description: Correlations among 1185 circulating metabolites and placental transcripts

Title: Supplementary Data 25

Description: Metabolon compound IDs

Title: Supplementary Data 26

Description: Gene ontology analysis of the 92 protein-coding transcripts which changed in the same direction in PE and FGR

Title: Supplementary Data 27

Description: List of differentially expressed genes by SGA sub-types Index Index of WGCNA data sets, (this information).

Title: Supplementary Data 28

Description: members Of Module _0 miRNAs and transcripts in module 0

Title: Supplementary Data 29

Description: members Of Module _1 miRNAs and transcripts in module 1

Title: Supplementary Data 30

Description: members Of Module _2 miRNAs and transcripts in module 2

Title: Supplementary Data 31

Description: members Of Module _3 miRNAs and transcripts in module 3

Title: Supplementary Data 32

Description: members Of Module _4 miRNAs and transcripts in module 4

Title: Supplementary Data 33

Description: members Of Module _5 miRNAs and transcripts in module 5

Title: Supplementary Data 34

Description: members Of Module _6 miRNAs and transcripts in module 6

Title: Supplementary Data 35

Description: members Of Module _7 miRNAs and transcripts in module 7

Title: Supplementary Data 36

Description: members Of Module _8 miRNAs and transcripts in module 8

Title: Supplementary Data 37

Description: members Of Module _9 miRNAs and transcripts in module 9

Title: Supplementary Data 38

Description: members Of Module _10 miRNAs and transcripts in module 10

Title: Supplementary Data 39

Description: members Of Module _11 miRNAs and transcripts in module 11

Title: Supplementary Data 40

Description: members Of Module _12 miRNAs and transcripts in module 12

Title: Supplementary Data 41

Description: members Of Module _13 miRNAs and transcripts in module 13

Title: Supplementary Data 42

Description: members Of Module _14 miRNAs and transcripts in module 14

Title: Supplementary Data 43

Description: members Of Module _15 miRNAs and transcripts in module 15

Title: Supplementary Data 44

Description: members Of Module _16 miRNAs and transcripts in module 16

Title: Supplementary Data 45

Description: members Of Module _17 miRNAs and transcripts in module 17

Title: Supplementary Data 46

Description: members Of Module _18 miRNAs and transcripts in module 18

Title: Supplementary Data 47

Description: members Of Module _19 miRNAs and transcripts in module 19

Title: Supplementary Data 48

Description: members Of Module _20 miRNAs and transcripts in module 20

Title: Supplementary Data 49

Description: members Of Module _21 miRNAs and transcripts in module 21

Title: Supplementary Data 50

Description: members Of Module _22 miRNAs and transcripts in module 22

Title: Supplementary Data 51

Description: eigengene Associati onTests_qVals qValues for the associations between module eigengenes and patient groups

Title: Supplementary Data 52

Description: enrichment Analysis Module_0 g: Profiler enrichment analysis for module 0

Title: Supplementary Data 53

Description: enrichment Analysis Module_1 g: Profiler enrichment analysis for module 1

Title: Supplementary Data 54

Description: enrichment Analysis Module_2 g: Profiler enrichment analysis for module 2

Title: Supplementary Data 55

Description: enrichment Analysis Module_3 g: Profiler enrichment analysis for module 3

Title: Supplementary Data 56

Description: enrichment Analysis Module_4 g: Profiler enrichment analysis for module 4

Title: Supplementary Data 57

Description: enrichment Analysis Module_5 g: Profiler enrichment analysis for module 5

Title: Supplementary Data 58

Description: enrichment Analysis Module_6 g: Profiler enrichment analysis for module 6

Title: Supplementary Data 59

Description: enrichment Analysis Module_7 g: Profiler enrichment analysis for module 7

Title: Supplementary Data 60

Description: enrichment Analysis Module_8 g: Profiler enrichment analysis for module 8

Title: Supplementary Data 61

Description: enrichment Analysis Module_9 g: Profiler enrichment analysis for module 9

Title: Supplementary Data 62

Description: enrichment Analysis Module_10 g: Profiler enrichment analysis for module 10

Title: Supplementary Data 63

Description: enrichment Analysis Module_11 g: Profiler enrichment analysis for module 11

Title: Supplementary Data 64

Description: enrichment Analysis Module_12 g: Profiler enrichment analysis for module 12

Title: Supplementary Data 65

Description: enrichment Analysis Module_13 g: Profiler enrichment analysis for module 13

Title: Supplementary Data 66

Description: enrichment Analysis Module_14 g: Profiler enrichment analysis for module 14

Title: Supplementary Data 67

Description: enrichment Analysis Module_15 g: Profiler enrichment analysis for module 15

Title: Supplementary Data 68

Description: enrichment Analysis Module_16 g: Profiler enrichment analysis for module 16

Title: Supplementary Data 69

Description: enrichment Analysis Module_17 g: Profiler enrichment analysis for module 17

Title: Supplementary Data 70

Description: enrichment Analysis Module_18 g: Profiler enrichment analysis for module 18

Title: Supplementary Data 71

Description: enrichment Analysis Module_19 g: Profiler enrichment analysis for module 19

Title: Supplementary Data 72

Description: enrichment Analysis Module_20 g: Profiler enrichment analysis for module 20

Title: Supplementary Data 73

Description: enrichment Analysis Module_21 g: Profiler enrichment analysis for module 21

Title: Supplementary Data 74

Description: enrichment Analysis Module_22 g: Profiler enrichment analysis for module 22
